# Optimized intravital three-photon imaging of intact mouse tibia links plasma cell motility to functional states

**DOI:** 10.1016/j.isci.2024.110985

**Published:** 2024-09-17

**Authors:** Asylkhan Rakhymzhan, Alexander F. Fiedler, Robert Günther, Scott R. Domingue, Laura Wooldridge, Ruth Leben, Yu Cao, Anne Bias, Jay Roodselaar, Ralf Köhler, Carolin Ulbricht, Judith Heidelin, Volker Andresen, Ingeborg Beckers, Astrid Haibel, Georg Duda, Anja E. Hauser, Raluca A. Niesner

**Affiliations:** 1 German Rheumatism Research Center – a Leibniz Institute, Biophysical Analytics, Berlin, Germany; 2 German Rheumatism Research Center – a Leibniz Institute, Immune Dynamics, Berlin, Germany; 3 Charité – Universitätsmedizin, Berlin, corporate member of Freie Universität Berlin and Humboldt-Universität zu Berlin, Clinics for Rheumatology and Clinical Immunology, Berlin, Germany; 4 Freie Universität Berlin, Dynamic and Functional in vivo Imaging, Berlin, Germany; 5 Thorlabs Inc., Lafayette, CO, USA; 6 Berlin University of Applied Sciences and Technology, Berlin, Germany; 7 Miltenyi Biotec GmbH, Bielefeld site, Bielefeld, Germany; 8 Charité – Universitätsmedizin, Berlin, corporate member of Freie Universität Berlin and Humboldt-Universität zu Berlin, Julius Wolff Institute, Berlin, Germany

**Keywords:** Small animal imaging, Optical imaging, Cell biology

## Abstract

Intravital deep bone marrow imaging is crucial to studying cellular dynamics and functions but remains challenging, and minimally invasive methods are needed. We employed a high pulse-energy 1650 nm laser to perform three-photon microscopy *in vivo*, reaching ≈400 μm depth in intact mouse tibia. Repetition rates of 3 and 4 MHz allowed us to analyze motility patterns of fast and rare cells within unperturbed marrow and to identify a bi-modal migratory behavior for plasma cells. Third harmonic generation (THG) was identified as a label-free marker for cellular organelles, particularly endoplasmic reticulum, indicating protein synthesis capacity. We found a strong THG signal, suggesting high antibody secretion, in one-third of plasma cells while the rest showed low signals. We discovered an inverse relationship between migratory behavior and THG signal, linking motility to functional plasma cell states. This method may enhance our understanding of marrow microenvironment effects on cellular functions.

## Highlights

• High pulse energy 1650 nm radiation is required to access the marrow of intact tibia
• Laser repetition rates of 3, and 4 MHz enable fast deep-marrow imaging in mouse tibia
• Third harmonics generation indicates protein synthesis ability in bone marrow cells
• Plasma cell migration and third harmonics generation signal are inversely linked

## Graphical abstract

**Figure:**
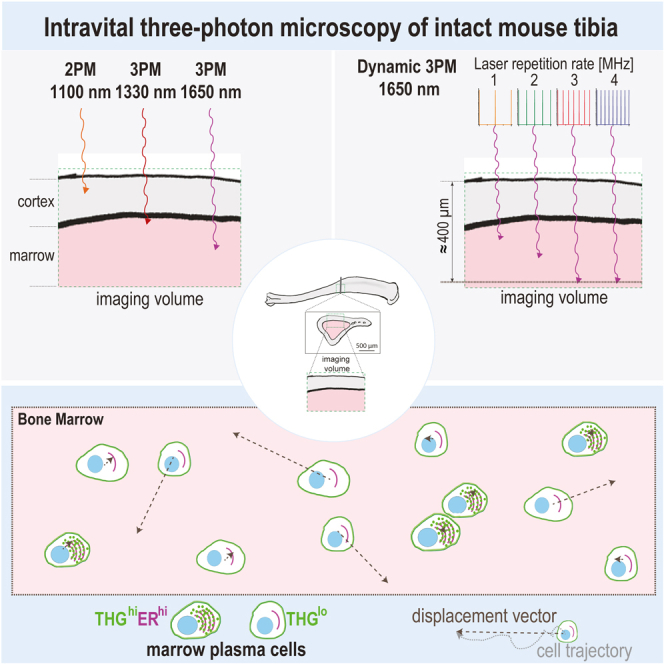

## Introduction

Intravital two-photon microscopy (2p.m.) in flat^1^ and long bones of mice^2^^,^^3^ allows us to study cellular migration, communication, and functions in bone marrow. As a primary lymphoid organ, the bone marrow is the birthplace of immune cells. This includes the generation of B cells, however, it also constitutes the final destination for terminally differentiated plasma cells, emerging from B cells activated in the periphery. Marrow plasma cells ensure long-term immunological memory through antibody secretion.^4^ *In vivo* 2p.m. has helped us to better understand plasma cell dynamics and interactions in the bone marrow microenvironment, on various time scales,^5^^,^^6^^,^^7^ which is essential for understanding mechanisms of their long-term survival *in situ.**^8^* Besides, *in vivo* bone marrow imaging in both flat and long bones has brought us insights into the development and dynamics of hematopoietic stem cells,^9^ osteoclast migration,^10^^,^^11^ as well as into tumor cell dormancy^12^ and metastasis dissemination.^13^^,^^14^ In long bones, *in vivo* 2p.m. has informed us about dynamic changes within mesenchymal and vascular compartments, in bone and marrow tissue,^15^^,^^16^ revealing the broad utility of this technology.

Recently, distinct molecular fingerprints and functions of the marrow tissue in the skull have been demonstrated,^17^ different from those of the marrow tissue in long bones. This finding emphasizes the need for dedicated *in vivo* imaging technologies for each bone type and marrow compartment.

Although optical imaging using near-infrared 2p.m. (700–1350 nm, ≈100 MHz repetition rate, pulse energy <5 nJ) in lymph nodes,^18^^,^^19^ spleen^20^ and hematopoietic islets in intact flat bones, e.g., calvarium,^21^ is broadly applied, scattering and absorption of radiation next to (scattering-related and unrelated) wave-front distortions impair the image quality with increasing tissue depth. *In vivo* near-infrared 2p.m. in the marrow cavity of long bones through intact bone cortex has been successfully performed,^10^^,^^11^ however, only in mice with thin bone cortex, such as young or γ-irradiated mice, and at shallow tissue depths.^22^ *In vivo* 2p.m. in long bones of adult mice, with thick bone cortex, has been previously performed either by mechanically thinning the overlaying bone tissue,^5^^,^^15^^,^^23^ or by inserting fixed micro-endoscopic probes in the femur.^24^ These approaches, are applicable in all mice independent of their age, and require invasive bone surgery, which initially causes an immune reaction and, by that, a transient perturbation of the tissue environment at the imaging site.^24^ Bone has the highest refractive index span among mammalian tissues (from *n* ≈ 1.33 up to ≈1.62).^25^^,^^26^^,^^27^ For comparison, the refractive index in soft lymphoid tissues ranges between ≈1.33 and ≈1.4.^26^^,^^27^^,^^28^ Therefore due to strong scattering effects in calcified bone, perturbation-free *in vivo* imaging of the marrow cavity through intact thick bone cortex in long bones cannot be achieved using near-infrared 2p.m.

In response to the challenges in near-infrared 2p.m., three-photon microscopy (3p.m.) using high pulse energy infrared radiation has been developed, to allow access to deeper tissue layers in various organs.^29^^,^^30^^,^^31^^,^^32^ Technological improvements such as the design of 3p.m. with Bessel beam illumination,^33^^,^^34^ multicolor 3p.m.,^35^ adaptive-optics enhanced 3p.m.,^36^^,^^37^^,^^38^ or miniaturized 3p.m. in freely moving mice^39^ helped to perform imaging at unprecedented tissue depths in the brain cortex and hippocampus. Moreover, 3p.m. allowed for structural and functional murine brain imaging through the intact skull (up to 100 μm thick),^32^ which requires wave-front distortion correction using customized adaptive optics.^36^ Besides, deep-tissue *in vivo* intra-tumoral 3p.m. imaging in skin and *ex vivo* imaging of ectopic ossicles with ≈70 μm thick cortical bone have been demonstrated.^40^ Next to fluorescence detection, 3p.m. enables the detection of third harmonics generation (THG), an elastic three-photon scattering process of coherent radiation, which provides label-free information about periodically organized molecular structures and about refractive index heterogeneity, e.g., in lipid bi-layers of cell or organelle membranes. In mouse tissue, THG has been associated with both cellular and extracellular structures^41^ and has been used to study myelination in the brain cortex,^42^ the geometry of osteocytes in the bone matrix^43^ or blood flow,^29^ relying on the THG signal of erythrocytes. Besides, oxygenation has recently been demonstrated to be indicated by THG and sum frequency generation, when using two spatiotemporally synchronized laser pulse trains.^44^ In leukocytes, a strong THG signal was shown to correlate with high cellular granularity.^45^^,^^46^

Addressing the need to monitor immune cell migration in deep regions of lymphoid organs,^47^ a customized 3p.m. setup has been designed to enable *in vivo* imaging throughout murine naive lymph nodes and in spleens. Hence, lymphocyte dynamics could be analyzed down to ≈600 μm depth in these secondary lymphoid organs.^29^ Although being a promising method for dynamic *in vivo* deep-marrow imaging in intact long bones of adult mice, high pulse energy infrared 3p.m. has neither been used in nor specifically adapted to this type of application.

Laser systems are adequate as excitation sources^31^^,^^35^ for successful deep-tissue 3p.m. must.

(i) emit at a radiation wavelength in one of the infrared spectral windows: 1300–1400 nm and 1600–1700 nm, respectively, for reduced signal loss in tissue,
(ii) have a high pulse energy and low repetition rate to secure moderate, biocompatible average power, and
(iii) feature temporally narrow pulses (<100 fs), to provide high photon flux density at the sample for efficient three-photon excitation.

Based on pulse chirping to support the generation of high pulse energy between 100 nJ and a few μJ, different configurations of optical parametric amplifiers (OPA) have been used as excitation sources for three-photon imaging in living tissue.^29^^,^^31^^,^^35^ Typically, OPA lasers are tunable between 1200 and 1700 nm at repetition rates from 0.33 to 1 MHz (rarely 2 MHz). The low repetition rate represents a disadvantage for *in vivo* 3p.m., as it limits the image acquisition speed over fields of view spanning several hundreds of μm. Soliton lasers have been successfully used as excitation sources to increase imaging depth in the mouse brain cortex by *in vivo* 2p.m., owing to their tunable repetition rates up to 10 MHz.^48^^,^^49^ The high repetition rates are favorable for increased image acquisition speed. However, their moderate pulse energy (<50 nJ) is potentially too low to efficiently induce three-photon events in tissue.

Here, we developed and integrated an OPA prototype in a customized multi-photon microscope system, to enable dynamic *in vivo* three-photon imaging in the marrow cavity of the intact tibia in adult mice. The high pulse energy radiation at 1650 nm enabled us to image marrow tissue at subcellular resolution through up to 200 μm thick bone cortex, reaching imaging depths of ≈400 μm in the tibial marrow. By tuning the OPA repetition rate up to 4 MHz, we were able to conduct a time-lapse 3p.m. over large fields of view (400 × 400 μm^2^) in the deep marrow cavity of intact unperturbed tibia *in vivo*. In this way, we visualized blood flow in the bone marrow vasculature and analyzed the motility even in rare cell subsets, such as plasma cells. Taking advantage of label-free THG signals, we found that the abundance of organelles corresponding to plasma cell functionality is tightly linked to the migration behavior of plasma cells *in vivo*.

## Results

### Microscope design for dynamic *in vivo* deep-tissue imaging in long bones

*In vivo* imaging of intact long bones requires the excitation laser beam to surpass two types of tissue layers: the hard cortical bone and the bone marrow, a soft lymphoid tissue. Laser power attenuation in both tissue types is caused by the scattering and absorption of radiation, in a wavelength-dependent manner.^28^^,^^50^ In the infrared spectral range, the absorption of radiation displays local minima at 1300 nm and 1650 nm, being lower in bone than in soft lymphoid tissue, due to the high water content of the latter.^27^^,^^28^^,^^51^ Notably, the absorption is stronger at 1650 nm than at 1300 nm for both tissue types. In contrast, the elastic scattering of radiation is stronger in bone than in lymphoid tissue at a given wavelength, and it steadily decreases with increasing wavelength.^27^ Taken together, the signal attenuation in bone tissue is lower at 1650 nm (8.7 cm^−1^) compared to 1350 nm (9.9 cm^−1^), and generally higher than in lymphoid tissues (5.4 cm^−1^ at 1330 nm and 7.1 cm^−1^ at 1650 nm). Calculations were made based on a previously published model of elastic radiation scattering^28^ and absorption spectra^27^ for both tissue types. Hence, we expected a superior performance of 1650 nm compared to 1350 nm excitation for *in vivo* imaging throughout the thick (>100 μm) tibia cortex, but the opposite holds true for the underlying soft marrow tissue.

**Figure 1:**
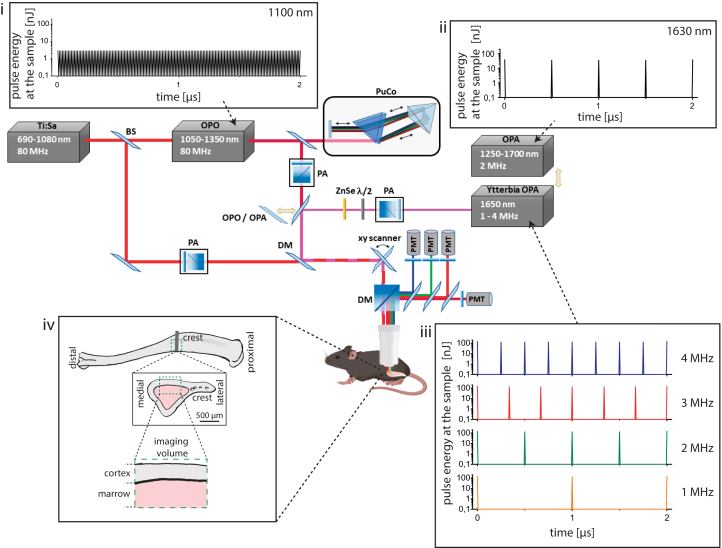
Customized setup design for dynamic deep-tissue imaging within intact long bones *in vivo* The schematics depict the main components of the customized multi-photon microscope. State-of-the-art two-photon imaging is performed using low pulse energy (<2 nJ) lasers, at 80 MHz, i.e., Ti:Sa and optical parametric oscillator (OPO, (i). The Ti:Sa beam is separated by a beam splitter (BS) into two optical pathways, one being used to pump the OPO, and the other being coupled into the microscope. Thus, the microscope covers a broad excitation range, i.e., 690–1080 nm (Ti:Sa) and 1050–1350 nm (OPO). Two types of optical parametric amplifiers (OPA), delivering high pulse energy radiation, are used as three-photon excitation sources. The Ytterbia OPA, a new OPA prototype, emits at a fixed wavelength (1650 nm, bandwidth 60 nm) and variable repetition rate between 1 and 4 MHz and delivers up to 130 nJ pulse energy at the sample (iii). The state-of-the-art OPA is tunable between 1250 and 1700 nm, at 2 MHz, and delivers up to 40 nJ pulse energy at the sample (ii). Power control of laser pulses was performed using power attenuation (PA) units consisting of a half-wave plate and a polarizing cube beam splitter. Due to the slightly positive group-velocity dispersion of our microscope, we built in a single-prism pulse compressor (PuCo) in the OPO beam path and a ZnSe window in the Ytterbia OPA beam path. A flip mirror (FM) is used to accurately switch between OPA and OPO irradiation regimes. The Ti:Sa and OPO beam paths are overlapped before being coupled into the microscope, using a dichroic mirror (DM). For imaging, laser beams are scanned over the sample using an x/y galvanometric scanner. Fluorescence, higher-harmonics generation signals, and excitation beams are separated using dichroic mirrors (DM), high-pass filters, and band-pass interference filters. Up to four photomultiplier tubes (PMT) are used for signal detection. The pulse trains of OPO and the two OPA systems are shown in (i–iii). Box (iv) shows that imaging was performed in the medial region (crest) of the mouse tibia. Imaging requires the laser beam to surpass first the bone cortex and then the bone marrow, i.e., a soft lymphoid tissue, tissue compartments having distinct absorption and scattering properties.

To retrieve adequate experimental parameters for fast dynamic deep-tissue tibia imaging over large 3D volumes, we adapted and characterized a state-of-the-art two-photon microscope setup to enable efficient two- and three-photon imaging in a broad infrared wavelength range (Figure 1; Figure S1; Table S1), as described in detail in star methods. Besides lasers typically used for two-photon microscopy (i.e., Ti:Sa and an optical parametric oscillator (OPO) operating at 80 MHz repetition rate in the range 700–1080 nm and 1050–1350 nm, respectively), we implemented a special optical parametric amplifier (OPA) as the excitation source. This OPA features a fixed wavelength (1650 nm, spectral bandwidth Δλ = 60 nm) and variable repetition rates (1.01–3.98 MHz), at fixed pulse energy and 65 fs pulse width (Figure S1; Table S1). This laser is termed hereafter Ytterbia OPA. For comparison, we used a state-of-the-art OPA laser, tunable in the range 1200 nm–1700 nm, at a fixed repetition rate of 2 MHz and <60 fs temporal pulse width.

In contrast to low-pulse-energy Ti:Sa and OPO systems (Figure 1I), the OPA lasers deliver high-pulse-energy radiation to the sample (Figures 1ii and 1iii), enabling efficient three-photon excitation and harmonics generation (Figures and Tables). Thus, they are adequate for three-photon imaging in the mouse tibia (Figure 1iv). The pulse energy values for selected wavelengths of the four lasers are provided in Table S1.

### Three-photon excitation by high pulse energy 1650 nm radiation is required for high-resolution *in vivo* imaging through the thick tibia cortex

Using our setup, we investigated the impact of excitation wavelength and pulse energy on the attenuation of radiation in bone and marrow tissue and, thus, on the imaging depth of *in vivo* multi-photon microscopy in long bones.

For this purpose, we performed *in vivo* 3D tibia imaging in adult Cdh5:tdTomato/Histone:GFP reporter mice (hereafter Cdh5:tdTom) at 1650 nm (Ytterbia OPA), at 1330 nm (state-of-the-art OPA) and at 1100 nm (OPO). TdTomato fluorescence was detected using all excitation wavelengths, being induced at 1650 nm by non-resonant (simultaneous) three-photon excitation, at 1330 nm by resonant (sequential) three-photon excitation, i.e., two-photon excitation followed by one-photon excitation, and at 1100 nm by non-resonant two-photon excitation (Figure S2). GFP fluorescence was detected only at 1330 nm, upon non-resonant three-photon excitation (Figure S2). In Cdh5:tdTom reporter mice, tdTomato is expressed in the membrane, GFP in the nuclei of endothelial cells, forming the blood vessel walls. 3D images were acquired over large volumes, i.e., up to 442 × 442 μm^2^ (517x517 pixels), in the medial area of the tibia (Figure 1iv) and up to 500 μm tissue depth, with step sizes of 2 or 4 μm.

**Figure 2:**
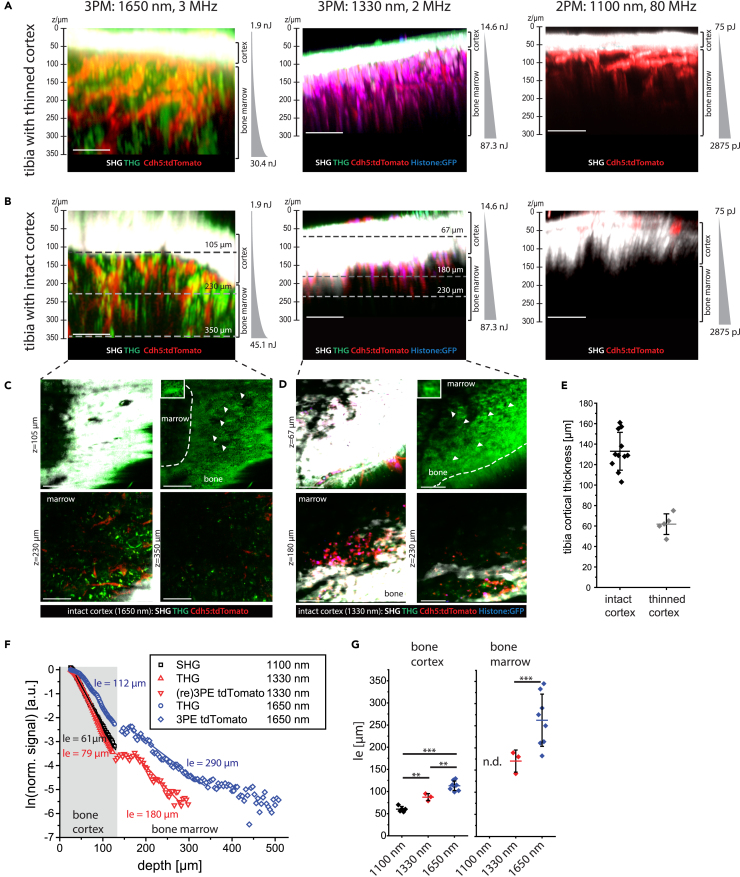
Minimizing attenuation of radiation by wavelength and pulse energy optimization for *in vivo* deep-marrow imaging in the intact mouse tibia, through >100 μm thick cortical bone (A) Axial xz 3D image projections acquired by three- (3p.m.) or two-photon microscopy (2p.m.) in tibia bones of Cdh5:tdTomato/Histone:GFP (Cdh5:tdTom) mice, through mechanically thinned bone cortex, <100 μm thick. tdTomato and GFP in endothelial cells (vasculature) are shown in red and blue. Second harmonics generation (SHG) and third harmonics generation (THG) are shown in white and green. 3p.m. was performed either at 1650 nm, 3 MHz using the Ytterbia OPA (left) or at 1330 nm, 2 MHz using the tunable OPA (middle). 2p.m. was performed at 1100 nm, 80 MHz using the OPO (right). All excitation schemes enable bone marrow imaging, with imaging depths >350 μm at 1650 nm, ≈300 μm at 1330 nm, and ≈200 μm at 1100 nm. (B) Axial xz 3D image projections acquired by 3p.m. or 2p.m. in intact tibia bones of the same mouse strain, through >100 μm bone tissue. 3p.m. and 2p.m. was performed as indicated in (A), showing that bone marrow imaging through thick bone is possible only by 3p.m. Imaging depths >350 μm are achieved at 1650 nm, but only ≈230 μm (endosteal areas) at 1330 nm. 2 p.m. at 1100 nm, 80 MHz enable only signal detection in the bone cortex, not in the marrow. (A and B) Indicated pulse energy and z-adaptation of power were chosen to prevent tissue damage. (C) xy projections corresponding to the tissue layers indicated by dashed lines in (B), left panel (3p.m., 1650 nm). SHG and THG signals in the bone cortex (105 μm depth) are shown in the upper panels. Arrowheads indicate THG signal in single lacunae (right), with an enlarged lacuna of an osteocyte within the tibia cortex as inset. Blood vessels (tdTomato) and THG are shown in endosteal areas (230 μm depth) and in deep marrow (350 μm depth). (D) xy projections corresponding to the tissue layers indicated by dashed lines in (B), middle panel (3p.m., 1330 nm). Similar signals as in (C) are detected in the bone cortex (67 μm depth) and in endosteal areas (180 μm, 230 μm depth), but not in deep marrow. (A–D) Scale bar = 100 μm. (E) Thickness of tibia cortex in the analyzed mice, either with mechanically thinned (*N* = 5 mice) or with intact cortex (*N* = 11 mice), determined relying on THG at 1330 nm and 1650 nm, and on SHG at 1100 nm. Mean values with s.d. are displayed. (F) Effective attenuation length *l*_*e*_ dependence on imaging depth z in the intact mouse tibia (>100 μm thick cortex). (G) *l*_*e*_ distribution in bone tissue and marrow (*N* = 5 mice at 1100 nm; *N* = 3 mice at 1330 nm; *N* = 8 mice at 1650 nm, mean values with s.d. are displayed). n.d. – not detected. Statistical analysis was performed using two-way ANOVA with Bonferroni post-test or t-test, significance: ∗*p* > 0.05, ∗∗*p* > 0.01, ∗∗∗*p* > 0.001.

As we expected stronger attenuation of radiation with increasing cortical bone thickness, we compared 3D images acquired in mice with intact, >100 μm thick tibia cortex (in average 136 μm, range 77 μm) and in mice with mechanically thinned cortex, <100 μm thick (in average 62 μm, range 28 μm) (Figures 2A–2D and S3). We did not perform depth correction of the 3D images, accounting for the refractive index in the specific tissue types,^52^ as its wavelength dependence is not known. Relying on data in other biological tissues,^53^ we expect a lower refractive index at larger excitation wavelengths, i.e., smaller depth correction.

Imaging of both fluorescence and higher harmonics generation in the bone cortex and bone marrow of thinned tibia in Cdh5:tdTom mice was successfully performed using all excitation schemes (Figure 2A). Through intact bone cortex thicker than 100 μm (Figure 2B), imaging the tibia marrow was possible only upon excitation at 1650 nm (Figure 2C) or at 1330 nm (Figure 2D), with larger imaging depth at 1650 nm than at 1330 nm. Excitation at 1100 nm did not permit imaging through intact cortical bone thicker than 100 μm. The cortical thickness in each analyzed mouse (Figure 2E) was determined based on the second harmonics generation (SHG) signal originating from collagen fibers in bone tissue, at 1650 nm, 1330 nm, and 1100 nm. The results were validated using the generation of the third harmonic (THG) signal originating from lacunae harboring osteocytes (Figure S9), their connecting canaliculi and collagen fibers in the bone cortex, at 1650 nm and 1330 nm.

Confirming these observations, imaging of marrow vasculature in the explanted tibia of over 100 week old Cdh5:tdTom female mice was possible at both 1100 and 1650 nm (Figure S4), as in old female mice the bone cortex is naturally thinner.^54^^,^^55^

To quantify the effect of excitation wavelength on the performance of deep-tissue tibia imaging, we assessed the effective attenuation length *l*_*e*_ in the bone cortex and bone marrow of Cdh5:tdTom mice with the intact tibia at 1650 nm, 1330, and 1100 nm (Figure 2F). According to Equations 29, 30, 31, 32, and 33 in Data S1, *l*_*e*_ is a standardized measure for the attenuation of radiation in tissue, defined as the tissue depth, at which the laser power decreases to 1/e of its value at the tissue surface (z = 0 μm).

At both 1650 nm and 1330 nm, the *l*_*e*_ values in the bone cortex are lower than in bone marrow (Figure 2G), in agreement with a larger refractive index variation and a stronger scattering in calcified bone as compared to soft hematopoietic tissues.^26^^,^^28^ The *l*_*e*_ values determined for bone cortex are larger at 1650 nm excitation (113 ± 11 μm, s.d.) when compared to 1330 nm (87 ± 8 μm, s.d.) and to 1100 nm (60 ± 6 μm, s.d.), in line with reduced scattering and lower signal attenuation at longer wavelengths,^29^ as indicated in the previous section. The same holds true for the *l*_*e*_ values measured in bone marrow, which were larger at 1650 nm (263 ± 59 μm, s.d.) as compared to 1330 nm (170 ± 25 μm, s.d.). In line with lower signal attenuation in lymphoid tissues compared to bone cortex, the *l*_*e*_ values in bone marrow are larger than in bone tissue for both 1330 nm and 1650 nm. As imaging of tibia marrow through intact, thick bone cortex was not possible at 1100 nm, corresponding *l*_*e*_ values cannot be determined. Notably, the interindividual variation of *l*_*e*_ values in bone tissue is lower (value range 30 μm at 1650 nm) than in bone marrow (value range 163 μm at 1650 nm), consistent with a higher zonal heterogeneity of tissue in the marrow as compared to bone cortex.

We expected that efficient three-photon excitation and third harmonics generation in deep-tissue tibia imaging require high pulse energy radiation. By comparing 3D images acquired in the intact tibia of a Cdh5:tdTom mouse *in vivo*, upon excitation at 1330 nm and average laser power 30 mW, at 80 MHz repletion rate and 2 MHz repetition rate, respectively, we showed that only excitation at high pulse energy (15 nJ at 2 MHz) but not at low pulse energy (0.38 nJ at 80 MHz) enables deep-marrow 3p.m. imaging in the intact tibia (Figure S4).

**Figure 3:**
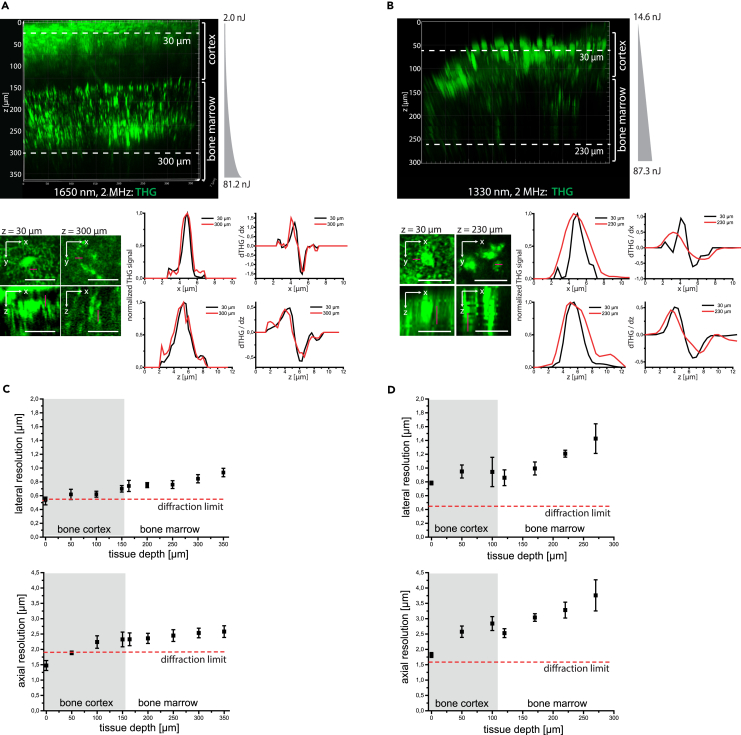
Three-photon imaging at 1650 nm enables sub-cellular resolution throughout the cortical bone and bone marrow in the intact tibia (A) 3D reconstruction (442 × 442 × 362 μm³, 1036 × 1036 × 362 voxel) of THG in the intact tibia, at 1650 nm, 2 MHz (upper panel). Representative xy and xz projections of THG in 30 μm depth in bone cortex and in 300 μm depth in bone marrow (bottom image array), tissue layers indicated in the upper panel. Scale bar = 30 μm. Representative intensity profiles of THG signal (left, corresponding to the magenta lines in the xy and xz projections) and their first derivatives (right), measured in 30 μm (black profiles) and 300 μm tissue depth (red profiles). (B) 3D reconstruction (442 × 442 × 300 μm³, 517 × 517 × 150 voxel) of THG in the intact tibia, at 1330 nm, 2 MHz (upper panel). Representative xy and xz projections of THG in 30 μm depth in the bone cortex, and in 230 μm depth in the endosteal area, as indicated in the upper panel. Scale bar = 30 μm. Similar to a THG intensity profiles (left, corresponding to the magenta lines in the xy and xz projections) and their first derivatives (right) are shown in 30 μm (black profiles) and 230 μm depth (red profiles). (A and B) Pulse energy and z-adaptation of power are indicated. (C) Depth dependence of lateral and axial resolution determined in the intact tibia upon excitation at 1650 nm, based on Gaussian approximation of THG intensity profiles and their first derivatives. For each tissue depth, at least 5 x- and 5 z-profiles were averaged (error bars show s.d.). (D) Depth dependence of lateral and axial resolution determined in the same manner as described for (C), at 1330 nm. (C and D) Axial and lateral resolution deteriorates with increasing imaging depth at both 1650 nm and 1330 nm excitation, with less degradation at 1650 nm. Thus, 3 p.m. at 1650 nm preserves subcellular resolution in the marrow cavity, with lateral resolution values better than 1 μm and axial resolution values of ≈2.5 μm, in 300 μm depth. The diffraction limit of the microscope was calculated based on the vectorial approximation,^56^ confirmed by 3p.m. of fluorescent nanospheres, at both 1650 nm and 1330 nm (Table S1), and displayed as dashed red lines. Mean values with s.d. for *n* = 5 measured structures are displayed for each data point in the graphs in (C) and (D).

Next to inducing the attenuation of radiation, tissue scattering leads to the degradation of spatial resolution with increasing imaging depth, as compared to diffraction-limited resolution (dashed red lines in Figures 3C and 3D). We assessed the depth-dependent degradation of spatial resolution in intact murine tibia (cortical thickness >100 μm) by analyzing xy- and z-profiles of THG signals upon excitation at 1650 nm (Figure 3A, magenta line profiles in upper and lower close-up images, respectively; Video S1) and 1330 nm (Figure 3B), respectively. Therefore, we approximated the xy- and z-profiles of the THG signal by Gaussian functions in canaliculi within the bone cortex (black profiles), and in bright, granular structures present within cells in the bone marrow (red profiles, left graphs in both Figures 3A and 3B). These results were confirmed by analyzing the first derivative of THG xy- and z-profiles, crossing edges of tissue structures both in the bone cortex (black profiles) and marrow (red profiles), i.e., ∂THG/∂x and ∂THG/∂z, respectively (right graphs in Figures 3A and 3B). The depth-dependent degradation of spatial resolution was more accentuated at 1330 nm than at 1650 nm excitation, in both bone cortex and bone marrow (Figures 3C and 3D). In the endosteal region, we measured 0.6 ± 0.1 μm lateral (upper panel Figure 3C) and 2.2 ± 0.2 μm axial resolution (lower panel Figure 3C) at 1650 nm, and 0.9 ± 0.2 μm (upper panel Figure 3D) and 2.8 ± 0.2 μm (lower panel Figure 3D) at 1330 nm. In the bone marrow, the lateral (upper panels Figures 3C and 3D) and axial resolution (lower panels Figures 3C and 3D) amounted to 0.9 ± 0.1 μm and 2.6 ± 0.2 μm at 1650 nm (in 300 μm tissue depth), and to 1.4 ± 0.2 μm and 3.8 ± 0.5 μm at 1330 nm (in 270 μm tissue depth). Thus, we concluded that high pulse energy laser radiation of 1650 nm wavelength is required for *in vivo* deep-tissue tibia imaging through intact bone cortices thicker than 100 μm.

Video S1. z-stack acquired in the intact tibia explanted from a C57/Bl6 mouse by three-photon-imaging at 1650 nm, 2 MHz rate repetition rate, related to Figure 3 The 3D image shows THG signal in a tissue volume of 442 × 442 × 362 μm³, 1036 × 1036 × 362 voxel, covering both cortical bone and bone marrow. The z-stack was used to determine the depth dependent spatial resolution in bone cortex and marrow, in the intact tibia (Fig. 3). Scale bar = 100μm

### Repetition rate optimization of 1650 nm radiation facilitates fast *in vivo* deep-marrow imaging in intact tibia

**Figure 4:**
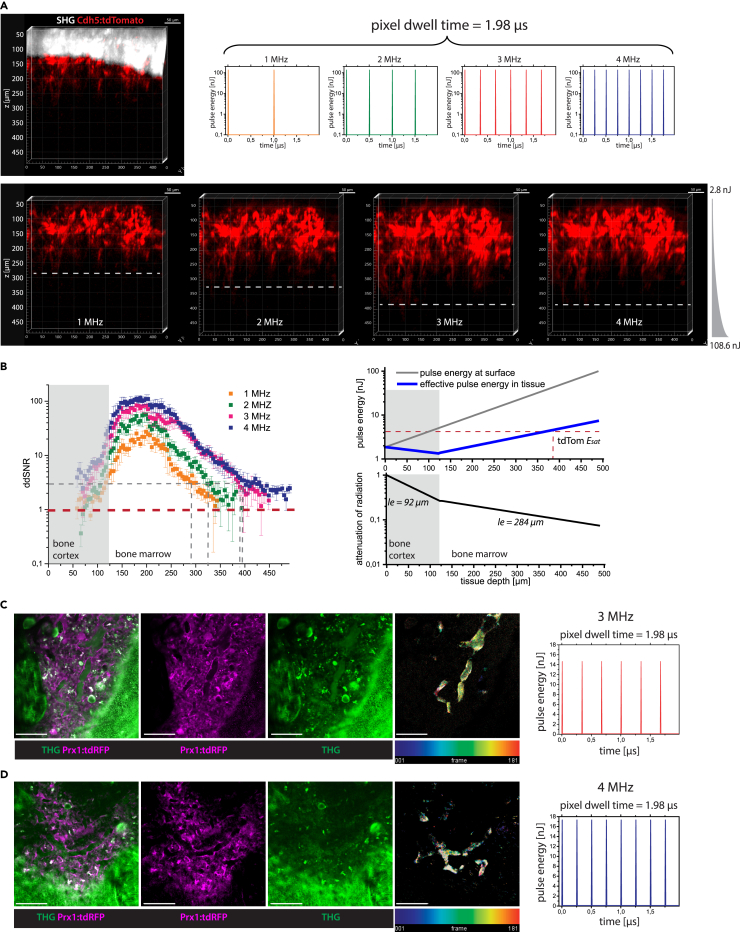
Laser repetition rate optimization for fast image acquisition in deep tissue layers of intact tibia by three-photon excitation at 1650 nm *in vivo* (A) 3D reconstructions (400 × 400 × 500 μm³, 518 × 518 × 125 voxel) of tdTomato (red) and SHG (white) in the intact tibia of a Cdh5:tdTom mouse at 1650 nm, with 1, 2, 3, and 4 MHz. Deeper imaging was achieved at higher repetition rates, as indicated by the dashed lines. The pulse trains at each repetition rate for 1.98 μs pixel dwell time are shown in the graphs. (B) *Left panel*: Depth dependent SNR determined for tdTomato fluorescence at 1 MHz (orange), 2 MHz (green), 3 MHz (red), and 4 MHz (blue). The absolute detection limit is given by SNR = 1 (red line). SNR = 3 is needed for reliable 3D object segmentation (gray line), being reached in 300 μm depth at 1 MHz, 340 μm at 2 MHz, and ≈400 μm at both 3 and 4 MHz. *Right panel*: Depth dependent applied pulse energy at the tibia surface (gray line) and effective pulse energy in tissue (blue line), upper graph. The effective pulse energy is the product of the pulse energy at the tibia surface and of the normalized attenuation of radiation in tissue (bottom graph). (C) First xy image (400 × 400 μm^2^, 518x518 pixel) of a time-lapse 2D stack acquired by *in vivo* 3p.m. in the tibia of a Prx1:tdRFP mouse at 1650 nm, 3 MHz (pulse energy 16 nJ, in 126 μm tissue depth, pulse train shown in graph). (D) First xy image of a similar time-lapse 2D stack as in (C) acquired at 4 MHz (pulse energy 14 nJ, in 120 μm depth, pulse train shown in graph). (C and D) tdRFP fluorescence (stroma compartment) is shown in magenta and THG in green. Among other tissue components, erythrocytes show a THG signal, enabling to visualization of blood flow in a label-free manner. Videos were acquired over 3 min, every second. To generate the time color-coded image (right images), we calculated the difference between every two consecutive THG images, color-coded the resulting images according to the acquisition time-point, and summed them up. In this way, only regions with changing structures, such as blood flow, are highlighted. 3 p.m. at both 3 and 4 MHz enables blood flow visualization over large fields of view in the tibia marrow. As at the same pulse energy, the average laser power at 3 MHz is lower than at 4 MHz, imaging at 3 MHz is less prone to induce tissue photodamage. Scale bar = 100 μm.

The average number of laser pulses per pixel rises with elevated laser repetition rate (graphs in Figure 4A), leading to a linear increase of detected signal (fluorescence, SHG, or THG), if pulse energy and pixel dwell time are kept constant. Conversely, for the same signal quality, higher repetition rates allow faster time-lapse imaging.

We took advantage of the variable repetition rate up to 4 MHz and high pulse energy, similar at all repetition rates (Table S1), to investigate the effect of higher repetition rates and, thus, of stronger signal, on imaging depth and image acquisition speed in the intact tibia *in vivo*. Fast imaging over large fields of view is key to track immune cells in the bone marrow, as it accounts for both cellular motility behavior and cell frequencies in the tissue. For instance, marrow B cells can migrate as far as 40 μm within 10 min.^57^

First, we performed *in vivo* 3D imaging of an intact tibia in a Cdh5:tdTom mouse, with 130 μm thick bone cortex, at 1650 nm and variable repetition rates, keeping pixel dwell time at a value of 1.98 μs, with 2x frame averaging (Figure 4A; Video S2). For all four repetition rates, we used the same exponential z-adaptation of pulse energy (2.76 nJ at z = 50 μm below the cortex surface, and 108.64 nJ at z = 488 μm tissue depth). For reconstructing these 3D images we performed depth correction assuming a refractive index of 1.55 for the bone cortex^25^^,^^26^ and of 1.38 for the bone marrow.^53^ We detected tdTomato fluorescence of endothelia in deeper layers of the tibia marrow, at 3 and 4 MHz repetition rate but not at 1 and 2 MHz, consistent with an increased number of laser pulses arriving at the sample within the same pixel dwell time (Figure 4A).

Video S2. z-stack acquired in the intact tibia of a Cdh5:tdTomato x Histone:GFP mouse by in vivo three-photon imaging at 1650 nm excitation, with 1 MHz, 2 MHz, 3 MHz or 4 MHz repetition rate, related to Figure 4 3D images show tdTomato fluorescence in the membranes of endothelial cells forming the blood vessel walls, within a tissue volume of 400 × 400 × 500 μm³, 751 × 751 × 126 voxel, the top layer laying in 70 μm tissue depth in the bone cortex. The data were used to determine the depth dependent signal-to-noise-ratio in the bone marrow (Fig. 4a). Scale bar = 100μm

To quantify this observation, we determined the depth-dependent signal-to-noise ratio (ddSNR) of the tdTomato fluorescence signal for all repetition rates (Figure 4B, left panel). SNR (Equation 36, Data S1) is defined as the ratio between the difference of measured fluorescence signal and mean background value and the width of the background count distribution, i.e., the noise. When the SNR value equals 1, the signal cannot be distinguished from noise, and therefore the maximum imaging depth is reached. We found that the ddSNR of tdTomato fluorescence decreased in the same manner for all laser repetition rates. As the SNR value scales up linearly with the repetition rate, for higher repetition rates the ddSNR reached the value of 1 in deeper marrow layers and, thus, the maximum imaging depth increased (SNR = 1 is marked as a dashed red line in Figure 4B). From our experience, reliable object segmentation, necessary for cell tracking, characterization, and classification, requires SNR values of at least 3 (dashed gray lines in Figure 4B), in agreement with previous reports.^40^ An SNR value of 3 was reached in ≈390 μm depth both at 3 MHz and 4 MHz repetition rate, deeper than in 280 μm at 1 MHz and in 325 μm at 2 MHz.

The imaging depth at 1 and 2 MHz repetition rate can be increased by increasing the pixel dwell time but impairing the image acquisition speed. Another strategy is to use a steeper z-adaptation of pulse energy at lower laser repetition rates (1 or 2 MHz), at the same incremental average laser power, i.e., the same exposure to thermal damage. However, as the applied increase of pulse energy with depth (Figure 4B, right panel) was chosen in such a way that the saturation energy *E*_*sat*_ of tdTomato (4.03 nJ, quantification described in Data S1) was reached in 388 μm depth, a steeper increase of pulse energy does not lead to fluorescence signal increase, but to highly non-linear photobleaching and, possibly, to non-linear phototoxicity. According to the z-adaption of power used in our experiment, the pulse energy applied to image marrow layers in 400 μm depth was 39.8 nJ (average laser power 119 mW at 3 MHz and 158 mW at 4 MHz).

Whilst excitation at 3 and 4 MHz repetition rates permitted similar maximum imaging depths in the intact tibia, the average power at 4 MHz is higher at the same pulse energy, which may lead to thermal and photodamage of tissue. Hence, we expected a repetition rate of 3 MHz to be more advantageous for *in vivo* time-lapse tibia imaging experiments, given it would allow for the visualization of fast biological processes, such as blood flow.

We performed *in vivo* time-lapse imaging of 400 × 400 μm^2^ areas (518x518 pixels) in the tibia of Prx1:tdRFP fate mapping mice at 1650 nm, at both 3 and 4 MHz, every second over 3 min, i.e., with 1 Hz acquisition rate and pixel dwell time 1.98 μs (Figures 4C and 4D; Videos S3 and S4). In Prx1:tdRFP fate mapping mice, mesenchymal stromal cells and their progeny express tdRFP. The pulse energy and average power values used for image acquisition, together with the location of the imaged tissue layer in the tibia are provided in Table S2. TdRFP fluorescence appeared throughout the marrow tissue, sparing vessel lumina, whereas THG signal was detected in both parenchyma and the vasculature (Figures 4C and 4D). Inside blood vessels, THG signal stemming from erythrocytes allowed us to visualize blood flow at distinct flow rates in different vessel types (Videos S3 and S4), in line with previous reports.^58^ By reducing the size of the field of view (200 × 200 μm^2^, 257x257 pixels, pixel dwell time 4 μs), we achieved even faster imaging at 2 Hz acquisition rate, i.e., a frame every 0.5 s, at both 3 and 4 MHz (last part in Videos S3 and S4).

Video S3. In vivo 2D time lapse imaging (400 × 400 μm2, 518x518 pixel) in the tibia marrow of a Prx1:tdRFP mouse performed at 1650 nm, 3 MHz, over 3 min, every second (Fig. 4c), followed by time lapse 2D imaging of a close up (150 × 150 μm2, 257x257 pixel) in the same region, over 1 min, every 0.5 s, related to Figure 4 Laser pulse energy, average laser power, and imaging area location in the tibia are indicated in Table II. tdRFP fluorescence (magenta) highlights the stromal compartment in the bone marrow and osteocytes in cortical bone, and THG signal (green) reveals tissue architecture in both bone cortex and marrow. THG signal of erythrocytes highlights blood flow within the vascular system in a label-free manner. Scale bar = 50 μm

Video S4. *In vivo* 2D time lapse imaging (400 × 400 μm2, 518x518 pixel) in the tibia marrow of a Prx1:tdRFP mouse performed at 1650 nm, 4 MHz, over 3 min, every second (Fig. 4days), followed by time lapse 2D imaging of a close up (150 × 150 μm2, 257x257 pixel) in the same region, over 40 s, every 0.5 s, related to Figure 4 Laser pulse energy, average laser power, and imaging area location in the tibia are indicated in Table II. tdRFP fluorescence is shown in magenta, THG signal is in green. Blood flow within the vascular system is visualized by the THG signal of erythrocytes. Scale bar = 50 μm

Concluding, we found similar performance at both 3 and 4 MHz repetition rates for fast *in vivo* marrow imaging. In the following experiments, we decided to employ excitation at 1650 nm and 3 MHz repetition rate, not exceeding 120 mW average power (39.8 nJ pulse energy), conditions we found necessary for imaging down to ≈400 μm depth in the marrow cavity of intact tibia. Next, we investigated whether these experimental conditions led to thermal or photodamage of the tissue or resulted in strong fluorophore photobleaching.

### Negligible tissue photodamage and tdRFP photobleaching during *in vivo* time-lapse tibia imaging at 1650 nm with 3MHz

Tissue photodamage induced by pJ to nJ pulse energy radiation, in the near-infrared and infrared range, is a complex process thought to originate from two main components: the bulk heating effect of infrared radiation throughout the exposed tissue, which scales linearly with the average laser power,^59^ and non-linear photodamage effects at the focal plane, induced by the high density of pulse energy.^51^ Due to the attenuation of radiation, the sample surface is most endangered by overheating, as it is exposed to a higher laser power than the underlying tissue.

To rule out overheating of the bone tissue during *in vivo* time-lapse deep-marrow imaging of intact tibia in mice, we set the average laser power of 1650 nm radiation, at 3 MHz repetition rate to 120 mW and performed repeated imaging of 400 × 400 × 30 μm³ volumes in the tibia marrow over 2 h, every 30 s, in tissue depths of 300–350 μm. We found no changes of either THG or SHG signals in the cortical bone layers above the imaged site in the tibia marrow, before and after *in vivo* time-lapse imaging (Figure S5). Furthermore, no signs of tissue damage were detected at the bone surface of 3p.m. imaged tibias using Nanofocus-CT, scanning electron microscopy and Movat’s pentachrome staining, compared to similar surface areas of non-irradiated contralateral bones (Figure S5). Thus, we concluded that 120 mW average laser power at 1650 nm and 3 MHz radiation does not thermally affect the bone tissue and is biocompatible for time-lapse *in vivo* imaging of intact tibia.

**Figure 5:**
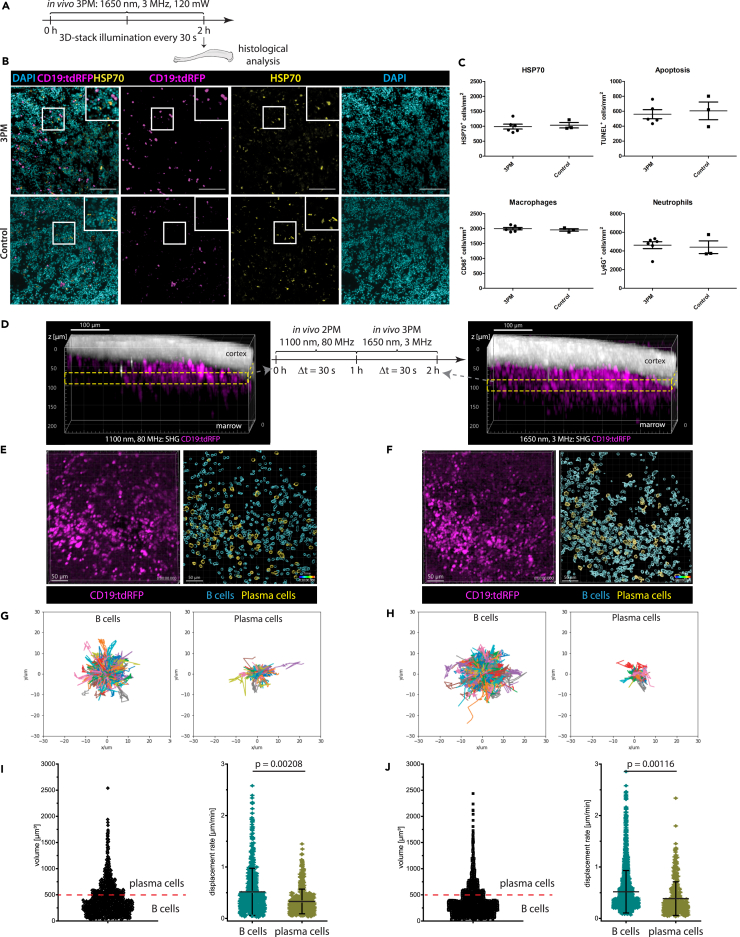
Multimodal tissue analysis reveals no signs of photodamage by *in vivo* time-lapse three-photon imaging at 1650 nm in mouse tibia (A) Experimental design for immunofluorescence histological analysis of marrow tissue after intravital 3 p.m. at 1650 nm, 3 MHz in the tibia of CD19:tdRFP mice. 3D imaging was performed over 2 h, every 30 s, at 40 nJ (120 mW). (B) Representative immunofluorescence overlays of B lineage cells (tdRFP, magenta), heat shock protein (HSP70, yellow) and nuclear staining (DAPI, cyan) in tibia marrow tissue irradiated by 3p.m. and not irradiated (control). Scale bar = 100 μm. (C) Photodamage quantification by immunofluorescence analysis: frequencies of HSP70^+^ cells, apoptotic cells (TUNEL^+^), macrophages (CD68^+^), and neutrophil granulocytes (Lys6G^+^) at 3p.m. irradiated marrow tissue (*n* = 6 mice) is similar to controls (*N* = 3 mice). This indicates no signs of tissue photodamage by 3p.m. (D) Experimental design of *in vivo* imaging to compare the effect of 3p.m. to state-of-the-art 2p.m. on the motility of marrow B lineage cells. The same marrow tissue site in the tibia of CD19:tdRFP mice, with thinned cortex, was imaged by time-lapse 2 p.m. at 1100 nm, 80 MHz, followed by time-lapse 3 p.m. at 1650 nm, 3 MHz. Yellow rectangles indicate the repeatedly imaged volume acquired by 2p.m. (left) and 3p.m. (right). B lineage cells (tdRFP) are shown in magenta and SHG in white. Scale bar = 100 μm. (E and F) Representative 3D images (400 × 400 × 30 μm³, 518 × 518 × 11 voxel) of marrow B lineage cells (tdRFP) acquired by time-lapse 2p.m. (E) and 3p.m. (F) (left images). Corresponding results of tdRFP^+^ cell segmentation (right images). tdRFP^+^ cells with a cellular volume between 65 and 500 μm³ are defined as B cells (cyan), and those with a volume between 500 and 4189 μm³ as plasma cells (yellow). Scale bar = 50 μm. (G and H) Rose plots representing the cell tracks of B cells (left; *n* = 1121 cells for 2p.m. and *n* = 2006 cells for 3p.m., in the same mouse) and plasma cells (right; *n* = 56 cells for 2p.m. and *n* = 136 cells for 3p.m.) over 30 min (2p.m. in G, 3p.m. in H). (I and J) Cell volume distribution of segmented tdRFP^+^ cells from the time-lapse data (left). Volume threshold of 500 μm³ (cell diameter 10 μm) is indicated by the red line. Mean displacement rate distributions of B cells and plasma cells, respectively (right). 2p.m. data are shown in (I), 3p.m. data in (J). (D–J) As the cell motility behavior of both marrow B cells and plasma cells is similar when analyzed by 2p.m. and by 3p.m., we conclude that 3 p.m. at 1650 nm, 3 MHz is reliable for assessing cell dynamics *in vivo*. Statistical analysis was performed using t-test, *p* values indicated, mean values are displayed, with s.d. ranges for displacement rate and mean velocity.

Next, using immunofluorescence analysis, we investigated possible damage at the imaged tissue site (300-350 μm total tissue depth) in the marrow of intact tibia bones induced by time-lapse *in vivo* imaging in CD19:tdRFP fate mapping mice, at 1650 nm, 3MHz (120 mW average power, 39.8 nJ pulse energy). In CD19:tdRFP fate mapping mice, tdRFP is expressed in B lineage cells. Time-lapse imaging was performed over 2 h, every 30 s. Thereafter the imaged bones were explanted and prepared for immunofluorescence analysis, using antibodies indicative of photodamage (Figure 5A). The non-irradiated contralateral tibia bones were used as controls.

No increase in the number of cells expressing heat shock protein 70 (HSP70),^31^ a marker of photodamage, was observed at marrow tissue sites irradiated at 1650 nm compared to non-irradiated tibia marrow (Figures 5B and 5C). Additionally, there was neither evidence of increased apoptosis analyzed by TUNEL staining, nor elevated numbers of neutrophils (Ly6G), or macrophages (CD68), which are known to respond in a rapid manner to (laser) damage, by migrating into the respective tissue regions^60^ (Figures 5C and S6). In contrast, clear signs of tissue photodamage using the same markers were detected by immunofluorescence analysis of spleens repeatedly exposed *in vivo* to excessive irradiation at 850 nm, 80 MHz, average power >300 mW (data not shown). A similar control experiment was not possible in intact tibia, as 850 nm radiation do not penetrate through thick bone cortex. However, repetitive *in vivo* 3p.m. imaging of intact tibia in Prx1:tdRFP mice every 0.5 s, at 1650 nm, 4 MHz and 512 mW (pulse energy 128 nJ, at tissue surface), induced microplasma-like damage to the tibia marrow, but not to the bone cortex (Video S5). Time-lapse imaging shown in Video S5 was acquired after 600 illumination steps, acquired every 0.5 s and performed through 106 μm cortex and 40 μm bone marrow. Hence the effective average power at the imaged site was 98 mW (pulse energy 24 nJ).

Video S5. *In vivo* 2D time lapse imaging (150 × 150 μm2, 257x257 pixel) in the tibia marrow of a Prx1:tdRFP mouse performed at 1650 nm, 4 MHz, following 600 irradiation steps (2 Hz), acquired every 0.5 s, related to Figure 4 Laser pulse energy was 128 nJ, average laser power 512 mW at the intact tibia surface. The imaging area was located beneath 100 μm cortical bone and 40 μm bone marrow, the effective pulse energy and average power being 24.5 nJ and 98 mW tdRFP fluorescence is shown in magenta, THG signal is in green. Photodamaged areas, presumably due to microplasma formation, appear in both tdRFP and THG channels (white), resulting in air bubbles appearing in the THG channel (green). Scale bar = 50 μm

To confirm that repeated exposure to 1650 nm, 3 MHz laser radiation does not induce tissue photodamage, we performed *in vivo* time-lapse 2 p.m. at 1100 nm, 80 MHz in the thinned tibia of CD19:tdRFP mice first, followed by *in vivo* time-lapse 3 p.m. at 1650 nm, 3 MHz at the same marrow site (Figure 5D). For both excitation schemes, time-lapse imaging of marrow tdRFP^+^ B lineage cells was performed over 60 min, every 30 s (Video S6), and their motility patterns were analyzed. To that end, noise was removed in the acquired 3D images using a trained Noise2Void algorithm,^61^ tdRFP^+^ cells were segmented (Figures 5E and 5F) and tracked over time (Figures 5G and 5H), as detailed in STAR Methods. Pulse energy, average power, and location of the imaged site in the medial region of tibia are summarized in Table S3 for both excitation schemes. The tdRFP^+^ B lineage cell population in the bone marrow comprises both B cells and plasma cells, as tdRFP expression is preserved also after the down-regulation of CD19 expression at later stages of B lymphocyte differentiation. We distinguished between B cells and plasma cells among the marrow tdRFP^+^ cells relying on their cell volume. We assumed a cell diameter of 5–10 μm for B cells (V < 500 μm³) and of 10–20 μm for plasma cells (V > 500 μm³) (Figures 5I and 5J), as previously reported.^8^^,^^24^ Confirming this assumption, we found tdRFP and GFP colocalization in 96% of marrow tdRFP^+^ cells with V > 500 μm³ in CD19:tdRFP x Blimp1:GFP mice (Figure S8). In Blimp1:GFP mice Blimp1^+^ plasma blasts and plasma cells express GFP. The motility characteristics of marrow tdRFP^+^ cells determined by 2p.m. and by 3p.m. were similar, with a mean velocity of 3.64 ± 0.76 μm/min (s.d.) and of 3.77 ± 0.79 μm/min (s.d.), respectively.

Video S6. *In vivo* 3D time lapse imaging (400 × 400 × 30 μm2, 518 × 518 × 11 voxel) in the tibia marrow of a CD19:tdRFP mouse performed by 2 p.m. at 1100 nm, 80 MHz, over 60 min, every 30 s, followed by 3 p.m. at 1650 nm, 3 MHz, again over 60 min, every 30 s (Fig. 5), related to Figure 5 The tibia cortex was mechanically thinned to allow 2p.m. imaging in the bone marrow. Laser pulse energy, average laser power and imaging area location in tibia are indicated in Table III. tdRFP fluorescence (magenta) highlights B lineage cells in the bone marrow. Scale bar = 100 μm

In previous reports, we and others demonstrated that B cells are more motile in the bone marrow than plasma cells. For both excitation schemes (1100 nm and 1650 nm excitation), we confirmed this finding, as highlighted by B cell and plasma cell tracks acquired over 30 min (rose plots in Figures 5G and 5H). We found mean displacement rates of 0.52 ± 0.23 μm/min (s.d.) for B cells and 0.36 ± 0.12 μm/min (s.d.) for plasma cells by 2p.m., and of 0.55 ± 0.20 μm/min (s.d.) for B cells and 0.38 ± 0.16 μm/min (s.d.) for plasma cells by 3p.m. (Figures 5I and 5J). We confirmed these findings in two replicate experiments (Table S3). The mean displacement rate values are in agreement with previous reports.^8^^,^^24^^,^^62^ Lower B lineage cell displacement rates were measured if time-lapse imaging was performed every 120 s as compared to every 30 s, as at lower sampling rates fast cells (typically B cells) are missed by the tracking algorithm.

Next, we analyzed the tdRFP photobleaching caused by *in vivo* 3 p.m. at 1650 nm, 3MHz compared to 2 p.m. at 1100 nm, 80 MHz (Figure S7). We found similar photobleaching rates of tdRFP fluorescence for both excitation schemes (*k*_*photobl*_ = 2.4 · 10^−3^ min^−1^ at 1100 nm, 25 mW and *k*_*photobl*_ = 1.3 · 10^−3^ min^−1^ at 1650 nm, 37 mW, Figure S7, at an 3D image acquisition rate of 2 min^−1^).

Owing to undetectable tissue photodamage and negligible fluorophore photobleaching, we concluded that the proposed 3p.m. method which uses 1650 nm, 3 MHz laser radiation at up to 40 nJ pulse energy is suitable to study cellular dynamics in the deep marrow of intact long bones *in vivo*, over large imaging volumes. This ability is particularly relevant when analyzing rare cell populations, such as the marrow plasma cells, and fast-moving cells, such as B cells.

### Cell motility and label-free third harmonic generation signal describe heterogeneity among marrow plasma cells *in vivo*

**Figure 6:**
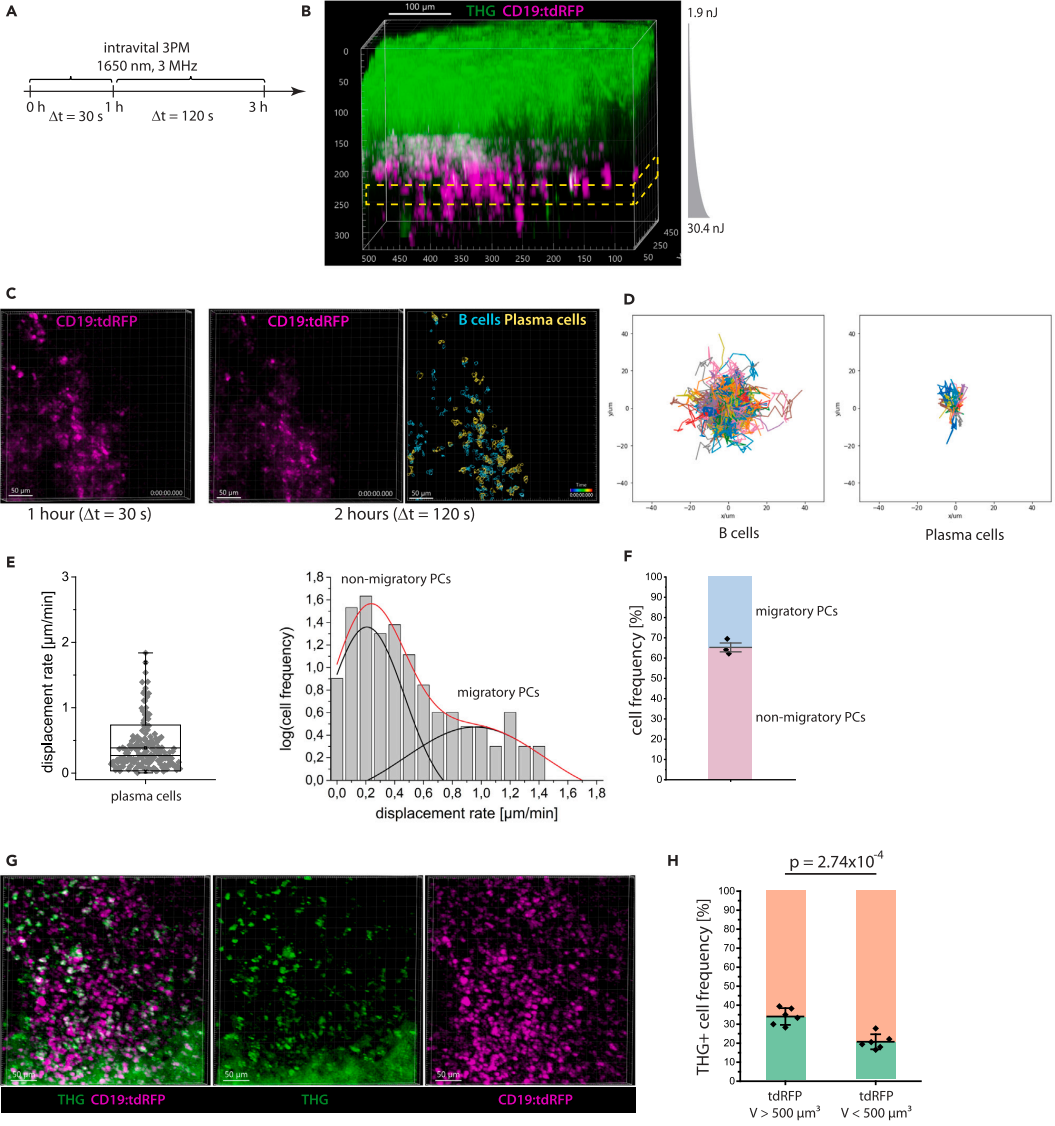
Heterogeneity within the plasma cell population in the tibia marrow is defined by both their cellular migration patterns and THG signal (A) Experimental design of *in vivo* time-lapse 3 p.m. at 1650 nm, 3 MHz in the intact tibia of CD19:tdRFP mice to study the migratory behavior of marrow plasma cells. A first acquisition period of 1 h, every 30 s is followed by acquisition over 2 h, every 120 s. (B) 3D image of intact tibia acquired by 3 p.m. at 1650 nm, 3 MHz (exponential pulse-energy z-adaptation: 1.9 to 30.4 nJ). THG is shown in green, B lineage cells (tdRFP) in magenta. The yellow rectangle indicates the repeatedly imaged volume (≈300 μm depth). Scale bar = 100 μm. (C) Representative 3D images of B lineage cells (400 × 400 × 30 μm³, 518 × 518 × 11 voxel) acquired during the first (left) and second imaging period (middle). Result of B lineage cell segmentation based on the data acquired during the second imaging period (2 h, right). B cells (cyan) and plasma cells (yellow) were defined by volume. Scale bar = 50 μm. (D) Rose plots representing cell tracks of B cells (left; *n* = 765 cells) and plasma cells (right; *n* = 64 cells). (E) Mean displacement rate distribution of marrow plasma cells assessed from the cell tracks measured over the entire 3 h imaging period in a single mouse (left). Logarithmic representation of cell frequency histogram with respect to the mean displacement rate (right), fitted by a double Gauss-peak distribution, indicating two distinctly motile plasma cell subsets in the tibia marrow. The black lines represent the Gaussian fitting functions, the red line represents their sum. (F) Relative cell frequencies of migratory and non-migratory marrow plasma cells in *N* = 3 CD19:tdRFP mice (mean values with s.d. range are displayed). (G) Merged (left) and single channel (middle, right) 3D images (400 × 400 × 100 μm³, 518 × 518 × 51 voxel) of tdRFP fluorescence (magenta) in B lineage cells and THG (green) in the intact tibia of a CD19:tdRFP mouse, by *in vivo* 3 p.m. at 1650 nm, 3 MHz, 21 nJ pulse energy, showing heterogeneous THG signal distribution among B lineage cells. Scale bar = 50 μm. (H) Percentage of THG^+^ cell numbers within the B cell and plasma cell population, respectively (*N* = 6 mice, mean values with s.d. range are displayed). Whereas THG signal is enriched in marrow plasma cells as compared to B cells, only 1/3 of the detected plasma cells display the signal. Statistical analysis was performed using t-test, *p* value indicated.

To analyze the dynamics of marrow plasma cells *in vivo*, we performed time-lapse deep-marrow 3p.m. (400 × 400 × 30 μm³, 518 × 518 × 11 voxels) in the intact tibia of CD19:tdRFP mice over a total of 3 h, at 1650 nm, 3 MHz excitation (Figures 6A and 6B; average power and pulse energy in Table S3). Within the first hour, we acquired a 3D image every 30 s, and in the following 2 h every 120 s (Figures 6A–6C; Video S7). Again, we found B cells to show larger displacement than plasma cells over a period of 2 h (rose plots in Figure 6D). Notably, the mean displacement rate distribution of marrow plasma cells, i.e., tdRFP^+^ B lineage cells with a cell volume between 500 and 4189 μm³, could be best approximated by a bi-modal Gaussian function (Figure 6E). This indicates two plasma cell subsets with distinct migratory behavior, 37.8% of the marrow plasma cells having a higher mean displacement rate of 0.96 ± 0.38 μm/min than 62.2% with a mean displacement rate of 0.21 ± 0.12 μm/min (*n* = 176 tracked plasma cells within an imaging volume). We could confirm our results in three independent experiments (Figures 6F and S8; Video S8). Our findings are in line with a report by Benet et al.,^5^ which showed both highly motile and non-migratory plasma cells by means of 2p.m. in the thinned tibia of Blimp-1 reporter mice.

Video S7. *In vivo* 3D time lapse imaging (400 × 400 × 30 μm2, 518 × 518 × 11 voxel) in the tibia marrow of a CD19:tdRFP mouse performed by 3 p.m. at 1650 nm, 3 MHz, over 60 min, every 30 s, followed by 120 min, every 120 s (Fig. 6), related to Figure 6 Laser pulse energy, average laser power, and imaging area location in the intact tibia, with 200 μm thick cortical bone, are indicated in Table III. tdRFP fluorescence (magenta) highlights B lineage cells in the bone marrow. Scale bar = 100 μm

Video S8. *In vivo* 3D time lapse imaging (400 × 400 × 30 μm2, 518 × 518 × 11 voxel) in the tibia marrow of a CD19:tdRFP mouse performed by 3 p.m. at 1650 nm, 4 MHz, over 120 min, every 120 s (Suppl. Fig. 8), related to Figure 6 Laser pulse energy, average laser power, and imaging area location in the intact tibia are indicated in Table III. tdRFP fluorescence (magenta) highlights B lineage cells in the bone marrow. Scale bar = 100 μm

By additionally analyzing the label-free THG signal in the acquired *in vivo* time-lapse 3D images within the tibia marrow of CD19:tdRFP mice (Figure 6G), we found enriched THG signal in plasma cells as compared to B cells (Figure 6H). For quantification, we segmented tdRFP^+^ cells and distinguished between B cells and plasma cells based on their volume (65μm³<V < 500 μm³ and 500μm³<V < 4189 μm³, respectively), as previously described^8^ and we analyzed the presence of a strong THG signal, defined as highest signal appearing in 1% of voxels, within the segmented cells. Despite an enrichment of THG signals in marrow plasma cells, only a minority (34.4 ± 3.3%) of these cells contained strong THG signals (Figure 6H).

Next, we addressed whether the heterogeneous distribution of THG signal in marrow plasma cells is related to the biological function of these cells and if it correlates with their motility patterns.

### Third harmonic generation^hi^ signal in marrow plasma cells reveals a link between endoplasmic reticulum abundance and cellular motility

**Figure 7:**
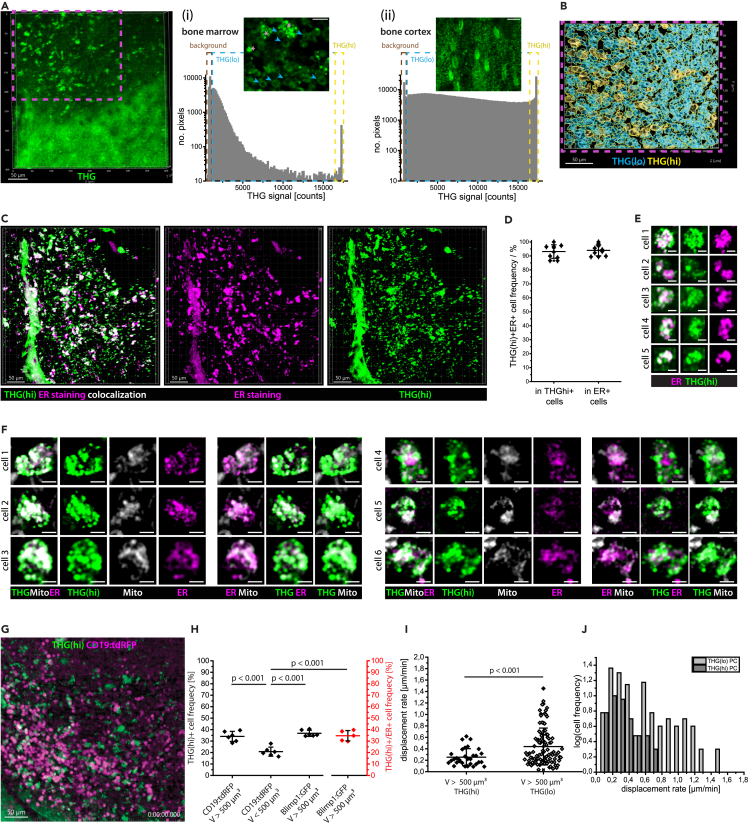
THG^hi^ signal defines two marrow plasma cell subsets with distinct functional capacity and migratory behavior (A) Representative 3D image (400 × 400 × 100 μm³, 518 × 518 × 11 voxel) of THG signal (green) in tibia cortex and marrow acquired by *in vivo* 3 p.m. at 1650 nm, 3 MHz. Scale bar = 50 μm. (i) Pixel distribution of THG signal in the tibia marrow: background (brown rectangle), THG^lo^ (cyan rectangle), and THG^hi^ (yellow rectangle). 2D image of THG signal in cells in the bone marrow, showing THG^lo^ signal in cell membranes (cyan arrowheads) and granular intracellular THG^hi^ signal (rose stars). Scale bar = 30 μm. (ii) Pixel distribution of THG signal in the tibia cortex, with the same color-coding for background, THG^lo^, and THG^hi^ pixels as in the bone marrow. 2D image of THG signals in the bone tissue, showing lacunae and connecting canaliculi. Scale bar = 30 μm. (B) Cell segmentation distinguishing between THG^lo^ (cyan) and THG^hi^ (yellow) cells (magenta rectangle in A). (C) Merged (left) and single channel (middle and right) 3D image (442 × 442 × 102 μm³, 1036 × 1036 × 52 voxel) of THG^hi^ (green) and endoplasmic reticulum (ER, magenta) in the bone marrow of an explanted C57/Bl6 mouse tibia (3 p.m. at 1650 nm). Scale bar = 50 μm. (D) Percentage of ER^+^ cells in the THG^hi^ cell population and of THG^hi^ cells in the ER^+^ cell population (*N* = 10 mice, each data point represents a mouse), showing a strong correlation of THG^hi^ signal and ER staining at the single cell level in the tibia marrow. Mean values with s.d. range are displayed. (E) Representative close-up images of THG^hi^ER^+^ cells, showing non-identical overlap of THG^hi^ signal and ER staining. Scale bar = 5 μm. (F) Representative close-up images of THG^hi^ cells in the tibia marrow, showing THG^hi^ signal originates both from ER (ER Tracker Red) and mitochondria (MitoTracker Deep Red). Scale bare = 5 μm. (G) Representative 3D image from a 60 min time-lapse *in vivo* 3p.m. video at 1650 nm, 3 MHz in the tibia of a CD19:tdRFP mouse (400 × 400 × 30 μm³, 518 × 518 × 11 voxel), every 30s. Scale bar = 50 μm. (H) Percentage of THG^hi^ cells in the CD19:tdRFP marrow cell populations with V > 500 μm³ (plasma cells) and with V < 500 μm³ (B cells), and in the Blimp1:GFP marrow cell population with V > 500 μm³ (plasma cells). The percentage of ER^+^THG^hi^ cells in the Blimp1:GFP ER^+^ cell population with V > 500 μm³ (plasma cells) is shown in red (mean values with s.d. range are displayed for *N* = 5 to 6 mice per case (each data points in the graph represents the results for a mouse) and >300 analyzed cells per mouse). (I) Distributions of mean displacement rates in the THG^hi^ and THG^lo^ plasma cell subset, respectively, showing that THG^hi^ plasma cells are less motile than their THG^lo^ counterparts (mean values with s.d. range are displayed). (J) Histograms of the mean displacement rates of THG^hi^ and THG^lo^ marrow plasma cells (data shown in I), highlight a bimodal distribution for THG^lo^ cells, in contrast to THG^hi^ cells. Statistical analysis was performed using two-way ANOVA with Bonferroni post-test or two-tail t-test, with *p* values indicated.

In line with previous reports,^41^^,^^45^ we observed a heterogeneous intensity distribution of THG both in tibia cortex and marrow by *in vivo* 3p.m. upon 1650 nm excitation. By analyzing the THG intensity both in bone cortex and bone marrow, we were able to distinguish between THG^lo^ and THG^hi^ signals (Figure 7A, left panel). In the tibia marrow, the THG^lo^ signal mainly originated from cell membranes (lipid bilayers), whereas THG^hi^ stemmed from granules inside cells, presumably the lipid bilayers of cell organelles (Figure 7Ai). This observation is in line with a previous study showing a higher granularity associated with strong THG signal in leukocytes.^45^ The THG^hi^ signal in bone cortex was associated mainly with lacunae, harboring osteocytes^63^ (Figures 7A(ii) and S9). Thin canaliculi connecting the lacunae showed a THG^lo^ signal. In the tibia marrow, we were able to use both THG^hi^ and THG^lo^ signals to segment single cells (cyan outlines for THG^lo^ cells, yellow outlines for THG^hi^ cells in Figure 7B). Time-lapse 3D imaging of these THG signals enabled monitoring of whole-tissue dynamics at cellular resolution, in a label-free manner (Video S9) and, thus, represents a promising strategy to assess tissue strain as well as shear stress in bone and bone marrow.

Video S9. *In vivo* 3D time lapse imaging (400 × 400 × 30 μm2, 518 × 518 × 11 voxel) of label-free THG signal in the intact mouse tibia performed by 3 p.m. at 1650 nm, 4 MHz, over 120 min, every 120 s, related to Figure 7 Laser pulse energy, average laser power, and imaging area location in the intact tibia are indicated in Table III. THG signals (green) show tissue architecture and strain in the bone cortex and bone marrow. During the video, the focus dived from the bone cortex, into the bone marrow, to highlight strain dynamics in tissues of different stiffness. Scale bar = 100 μm

As we assumed that a highly granular THG^hi^ signal inside cells stems from organelles, rich in lipid bilayers, we investigated whether the endoplasmic reticulum (ER) is abundant in THG^hi^ cells of the bone marrow. Therefore, we labeled the ER in explanted tibia bones of C57/Bl6J mice with ER-Tracker red (BODIPY glibenclamide) and performed 3D imaging in the bone marrow at 1650 nm (Figure 7C). In these 3D images, we segmented THG^hi^ cellular structures with a volume between 65 and 4189 μm³ and counted cells showing abundant ER tracker fluorescence. Additionally, we segmented ER^+^ cells in the same cell volume range and counted those cells displaying the THG^hi^ signal. The detection limit of abundant ER tracker fluorescence signal was defined as the highest background count measured within cortical bone areas, in which no ER is expected to be present (Figure S9). We found that 93.1 ± 4.9% of segmented THG^hi^ cells in the bone marrow display ER tracker fluorescence, and 94.0 ± 3.9% of the segmented ER^+^ cells display THG^hi^ signal (Figure 7D). Thus, we concluded that THG^hi^ signal is indicative for abundant ER in bone marrow cells, needed for protein biosynthesis. We expect that THG^lo^ cells also contain ER, however, at a lesser extent than THG^hi^ cells.

Notably, we found that ER tracker and THG^hi^ signal only partially co-localize in the ER^+^ THG^hi^ double-positive cells (Figure 7E). This observation indicates that THG^hi^ signals originate also from membrane-rich organelles other than ER, such as mitochondria (Figure 7F). We may not detect the THG^hi^ signal of certain ER or mitochondrial structures due to discrepancies between laser polarization and orientation of the lipid bilayers in the ER membrane.

We confirmed the validity of our finding that only ≈1/3 of the plasma cells show THG^hi^ signal in the tibia marrow of CD19:tdRFP mice (Figure 7G), by analyzing explanted tibia bones of Blimp1:GFP mice (Figure S9). Besides plasma blasts and plasma cells, Blimp-1 is expressed in other cell types. The expression level in other cell types is however much lower than in plasma blasts and plasma cells, and their cell volumes are typically lower than those of plasma cells. Hence, we were able to unequivocally identify plasma cells and plasma blasts in the tibia marrow of Blimp1:GFP mice by three-photon 3D imaging at 1330 nm (Figure S9). We segmented GFP^+^ cells having a volume between 500 and 4189 μm³ and analyzed the presence of THG^hi^ signal in these cells. Similar to the data acquired in CD19:tdRFP mice, we found that 36.7 ± 2.7% of the GFP^+^ plasma cells are THG^hi^.

In some of the explanted tibia bones from the Blimp-1:GFP mice we additionally labeled the endoplasmic reticulum using ER tracker and performed 3D three-photon imaging at 1330 nm (Figure S9). Using the previously published SIMI algorithm for spectral unmixing^59^^,^^64^ followed by cell segmentation, we found that GFP^+^ THG^hi^ ER^+^ cells represent 34.6 ± 4.5% of all GFP^+^ cells with a volume between 500 and 4189 μm³ in the tibia marrow. Neither GFP^+^ THG^hi^ cells without ER tracker signal nor GFP^+^ ER^+^ cells without THG^hi^ signal were detected. Thus, the THG^hi^ signal is associated with abundant ER also in marrow plasma cells, indicating that THG^hi^ plasma cells may have a higher capacity of antibody production, as compared to THG^lo^ plasma cells. Along this line, we found that B cells stimulated by LPS over 48 h, known to secrete high amounts of antibodies, show THG^hi^ signals. Upon treatment with 100 nmol/L rapamycin over 12 h, known to induce a significant reduction of antibody secretion,^65^ these cells lose their THG^hi^ signal (Figure S10). Reduction in IgM secretion upon rapamycin treatment was confirmed by enzyme-linked immunosorbent assay (ELISA) of the cell supernatant (Figure S10). Thus, the THG^hi^ signal indeed resolves between two functionally distinct plasma cell subsets in the bone marrow (Figure 7H).

How the functional state of marrow plasma cells indicated by the THG^hi^ signal relates to plasma cell motility can only be addressed by dynamic *in vivo* imaging experiments in the deep marrow of long bones. Our cell tracking analysis of plasma cells in the tibia marrow of CD19:tdRFP mice *in vivo*, at 1650 nm, 3MHz excitation (Figure 7G and Video S10), showed that plasma cells colocalizing with THG^hi^ signal have a lower mean displacement rate (0.25 ± 0.07 μm/min, *n* = 31 cells) than THG^lo^ plasma cells (0.44 ± 0.16 μm/min, *n* = 100 cells), in Figure 7I (results validated in *N* = 3 mice). This result indicates that THG^hi^ plasma cells are sessile (monomodal mean displacement rate distribution), whereas THG^lo^ plasma cells show a bi-modal mean displacement rate distribution, comprising both sessile and migratory cells (Figure 7J).

Video S10. *In vivo* 3D time lapse imaging (400 × 400 × 30 μm2, 518 × 518 × 11 voxel) in the tibia marrow of a CD19:tdRFP mouse performed by 3 p.m. at 1650 nm, 3 MHz, over 60 min, every 30 s (Fig. 7), related to Figure 7 Laser pulse energy, average laser power, and imaging area location in the intact tibia are indicated in Table III. tdRFP fluorescence (magenta) highlights B lineage cells in the bone marrow and the THG^hi^ signal (green) indicates cells in the bone marrow with abundant endoplasmic reticulum. Scale bar = 100 μm

## Discussion

### Development of dynamic *in vivo* three-photon imaging in intact tibia

As the birthplace of most hematopoietic cells,^9^^,^^66^ responsible for immunological memory maintenance,^5^^,^^6^^,^^67^ and being involved in tumor dormancy and metastatic recurrence,^14^ the bone marrow microenvironment has been intensively investigated. To give insight into the sequence of events contributing to these long-term processes spatiotemporal studies are of particular importance. Intravital two-photon microscopy (2p.m.) allowed us to analyze motility patterns of immune cells and their interactions with the environment, in murine marrow of flat bones,^21^ of intact long bones of young or irradiated mice,^10^^,^^11^ after surgical thinning of the bone cortex in adult mice,^5^^,^^15^^,^^68^ or by inserting micro-endoscopic lenses into the marrow cavity.^24^^,^^69^ However, minimally invasive technologies for long bone imaging are still needed.

As there is increasing evidence for functional differences in the immune compartment between the hematopoietic islands in the skull, a flat bone, and the marrow of long bones,^17^ dedicated *in vivo* imaging methods are required for the various bone types. The interactions of immune cells with the stromal and vascular compartments have been shown to impact on immune cell functions in the bone marrow.^4^^,^^8^^,^^9^ The need for *in vivo* imaging methods in long bones is supported by the fact that the high mechanical load specific for this bone type, as opposed to flat bones, is expected to have a strong impact on osteo-immune interactions.^70^ As in long bones, endosteal areas and deep marrow were found to differ with respect to microenvironmental conditions,^4^^,^^24^^,^^71^ developing technologies which allow dynamic imaging in the deep marrow cavity is key to understand immune cell functions.

Scattering and absorption of near-infrared radiation in two-photon microscopy limit highly resolved, direct optical access to areas located deep within the marrow cavity of intact long bones, through >100 μm thick cortical bone. The straightforward answer to this challenge is the use of long wavelength, infrared radiation at high pulse energy, with narrow fs-pulses, to favor higher order non-linear processes, such as three-photon excitation.^31^^,^^35^ State-of-the-art optical parametric amplifiers (OPA) have been previously used as excitation sources for three-photon microscopy (3p.m.), to image various organs and tissues, *in vivo* and *ex vivo*,^29^^,^^30^^,^^32^^,^^35^^,^^40^^,^^44^ even in freely moving mice.^39^ Notably, 3p.m. and even 4 p.m. at 1650 nm (1 MHz) was successfully performed in ossicles *ex vivo*, with approx. 70 μm bone matrix.^39^ However, *in vivo* 3p.m. of deep marrow cavity in intact long bones of adult mice, with bone representing the most scattering tissue in living organisms,^26^ has not yet been performed, and adequate 3p.m. setup parameters have not yet been defined for this application. Besides, a main challenge of existing OPA systems for 3p.m. is imposed by their low repetition rate (0.3–2 MHz^29^), leading to slow image acquisition over large fields of view, and, by that, limiting the capacity to investigate dynamic biological processes in the bone marrow, such as immune cell migration. For instance, T cells, the fastest cell population in secondary lymphoid organs, migrate at speeds up to 15 μm/min,^72^ marrow B cells travel up to 40 μm within 10 min.

In this study, we demonstrate fast dynamic *in vivo* three-photon imaging of 400 × 400 μm^2^ tissue areas in the bone marrow of intact mouse tibia, using an OPA prototype as 3p.m. excitation source, which emits high pulse energy (>400 nJ) radiation at 1650 nm and repetition rates in the range 1–4 MHz. The generation of 3 and 4 MHz under the specified conditions is a unique feature of this OPA prototype. We succeeded to monitor heterogeneous blood flow dynamics and to analyze B lymphocyte motility in the marrow cavity of tibia, through up to 200 μm thick cortical bone, when using 3 and 4 MHz repetition rates, at 20–40 nJ pulse energy at the sample. In benchmarking experiments comparing our 3p.m. technology to existing 2p.m. and 3p.m. methods, we demonstrated that these laser parameters are key to achieving (i) highly resolved imaging as deep as ≈400 μm in the marrow cavity of intact mouse tibia, (ii) imaging in a time-lapse manner, without any evidence of tissue photodamage neither in bone cortex nor in bone marrow. 400 μm imaging depth in intact tibia corresponds to ≈1.5-fold of the effective attenuation length *l*_*e*_ of radiation in this organ. As imaging depths as high as 10x *l*_*e*_ have been theoretically predicted,^73^ we expect that further technological improvements, e.g., adaptive wave front correction, already demonstrated for brain cortex imaging through 100 μm skull bone,^36^^,^^38^ will enable imaging throughout the entire volume of long bones in the future. A long excitation wavelength (1650 nm) complements and supports such a correction of wave front distortions in long bones, as it enables more laser pulse energy for efficient signal generation arrives at the imaging site deep within tissue, and that emitted (longer wavelength) fluorescence and higher harmonics signals, typically in the visible spectral range, reach the detector. This is particularly relevant in intact long bones, as both incoming and outgoing radiation is diminished by scattering through the thick bone cortex. In the same line, the drawback of 1650 nm radiation able to efficiently excite red fluorescent proteins and dyes, but not broadly used fluorophores, such as GFP, seems to be marginally relevant for intact long bone imaging, as short wavelength fluorescence will hardly succeed to surpass the overlaying bone and marrow tissue layers. A solution to further reduce scattering is the use of even longer excitation wavelengths and of fluorescent proteins and dyes emitting in the infrared range,^74^^,^^75^ which call for further laser and microscope optics development,^76^ generation of infrared fluorescent proteins and of reporter mice based on those.

Reduced scattering at higher excitation wavelengths and the cubic dependence of laser photon flux density for non-resonant three-photon excitation and THG^77^ allowed us to achieve the highest spatial resolution upon excitation at 1650 nm, in the deep marrow cavity of intact tibias, despite the expectation of higher diffraction-limited spatial resolution (i.e., at tissue surfaces).^78^ The subcellular spatial resolution of 3 p.m. at 1650 nm in deep tissue is underlined by high SNR values. To further reduce noise and, by that, to improve SNR for better cell segmentation and cell tracking in our data, we trained and successfully applied an existing deep-learning algorithm, i.e., Noise2Void,^61^ on time lapse 3D fluorescence images of tdRFP^+^ B lineage cells in the tibia marrow. Experimental SNR improvement can be achieved by time-gated detection, as applied at 3p.m. of the brain cortex.^36^ This represents a reliable strategy, as even for higher OPA repetition rates (3 and 4 MHz), noise detection temporally surpasses the detection of fluorescence and harmonics generation signals by several orders of magnitudes. However, for the detection of phosphorescence, e.g., when using oxygen-sensitive probes^79^ to monitor tissue oxygenation *in vivo,*^80^ time-gating is no longer favorable, as phosphorescence lifetimes are in the same range as the time window between two consecutive laser pulses of OPA systems.

The downside of using high pulse-energy long-wavelength radiation for *in vivo* imaging is the inherent danger of damaging the tissue. By performing immunofluorescence analysis, we have shown that non-resonant three-photon excitation at 1650 nm, while potentially expected to overheat biological samples, is not associated with tissue thermal or photodamage in tibia, up to an average power of 120 mW. We verified this finding by monitoring intact blood flow *in vivo*, in the tibia marrow at 1650 nm, 3 and 4 MHz. Along the same line, we found that marrow B lymphocyte mean velocity and displacement rate values measured by 3 p.m. at 1650 nm were similar to those measured by 2 p.m. at 1100 nm, in the same tissue area. No signs of damage were detected at the bone surface and in the bone cortex above the imaged marrow sites.

We assume that resistance to photodamage at such high laser powers is related to the fact that the soft marrow tissue is protected by bone cortex, the last being less prone to photodamage, presumably due to low water content. In other organs, the average power limit of photodamage at similar laser wavelengths was found to be lower, i.e., 100 mW in brain cortex^32^ or 80 mW in lymph node.^29^

Finally, photobleaching of tdRFP, the red fluorescent protein used for time-lapse *in vivo* imaging, was negligible confirming the reliability of the described 3p.m. method.

### 3p.m. and label-free third harmonics generation *in vivo* imaging provides new insights into marrow biology and links functional capacity to the migratory behavior of single plasma cells

*In vivo* label-free THG imaging using our 3p.m. method highlights the 3D tissue architecture of both bone and marrow compartments in the intact tibia, with high fidelity, at subcellular resolution. Performed in a time-lapse manner, we could show that THG imaging enables monitoring of whole-tissue dynamics and may represent a powerful tool to monitor mechanical cues, such as shear stress in vessels and tissue strain, both in stiff and soft tissues. Prospectively, the THG signal will be able to inform about the 3D force field acting on single immune cells *in vivo*, with an impact on their functions.^70^

Moreover, the in-depth analysis of the THG signal in the tibia marrow allowed us to reliably associate the THG^hi^ signal in cells with a high organelle content distinguishing these cells from THG^lo^ cells, in which the THG signal mainly originates from the cell membrane, in line with previous reports on isolated leukocytes.^45^ As we found THG^hi^ signal to be unequivocally associated with abundant endoplasmic reticulum (ER) in all marrow cells, this label-free, the ubiquitous signal has the potential to indicate cellular functional states associated with protein biosynthesis in any cell type, if co-registered with cell type specific labeling, e.g., in fluorescent reporter mice. Additionally, as we found that the THG^hi^ signal in marrow cells originates not only from abundant ER but also from mitochondria, we expect that the co-registration of all these signals might be useful to further classify the phenotypes and differentiation stages of marrow plasma cells, using dedicated deep-learning algorithms, as previously shown for leukocytes in bronchoalveolar lavage.^46^

Analysis of *in vivo* 3p.m. data acquired in the unperturbed tibia marrow of B lineage reporter mice allowed us to show that the THG^hi^ signal is enriched in plasma cells as compared to B cells. However, only ≈1/3 of marrow plasma cells were THG^hi^, shown also to have abundant ER. As it has been previously shown that ER is required to produce large amounts of antibodies, we conclude that the THG^hi^ signal defines heterogeneity of functional capacity among marrow plasma cells *in vivo*. Along this line, B cells massively expand their ER when differentiating into plasma cells, to increase their antibody producing function.^81^

Our 3p.m. method enables intravital time-lapse imaging of currently inaccessible marrow regions in intact tibia of B lineage reporter mice, over large fields-of-view. Hence, we could analyze the migration behavior of marrow plasma cells over up to 3 h, in a statistically reliable manner, as we could monitor >100 plasma cells per animal. In line with previous reports,^5^ we found two plasma cell subsets with distinct motility patterns, i.e., a non-migratory and a highly motile subset, characterized by mean displacement rates of ≈0.2 μm/min and ≈1 μm/min, respectively.

By co-registering THG signal with plasma cell labeling in the time-lapse 3p.m. data, we found that THG^hi^ plasma cells are non-migratory, with a mean displacement rate of ≈0.25 μm/min, whereas highly motile cells are THG^lo^ plasma cells, with a mean displacement rate of ≈0.45 μm/min. These data suggest that the capacity of plasma cells to produce large amounts of antibodies is inversely linked to their migratory behavior. Plasma cells residing in their survival niches are non-migratory,^5^^,^^8^ and are able to produce large amounts of antibodies, as in these niches they find suitable microenvironmental conditions supporting their metabolic demands.^80^ In contrast, migratory plasma cells, including plasma blasts as precursors of sessile plasma cells,^82^ presumably searching for an appropriate microenvironment, may have a limited capacity for protein biosynthesis, as previous reports showed varying antibody secretion by plasma cells dependent on extrinsic factors in tissue.^83^

Taken together, in this study, we introduced a laser with 3 and 4 MHz repetition rates of high-pulse-energy 1650 nm radiation and used it as the excitation source for dynamic *in vivo* three-photon imaging of intact mouse tibia. In this way, we were able to access the deep marrow cavity in a minimally invasive manner and, together with in-depth analysis of ubiquitous label-free THG signals and cell type specific fluorescence, we identified links between cellular motility patterns and functional capacity related to protein synthesis, opening unprecedented opportunities to understand bone biology *in vivo*.

### Limitations of the study

While the 3p.m. method for deep-marrow cavity imaging in intact long bones introduced by us retains great potential for extensive analysis of bone biology, few limiting issues need to be tackled in the future. Radiation at 1650 nm is not easily compatible with the excitation of GFP and its variants, typically used for fluorescent reporter mice. Therefore, four-photon excitation (4p.m.) would provide a solution. However, while reported,^40^ 4p.m. implies much higher photon flux densities and, by that, much higher pulse energies, potentially increasing the risk of tissue photodamage.

Scattering of radiation at 1650 nm in bone tissue still leads to power loss, limiting the imaging depth in intact bones, and calling for even longer excitation wavelengths, above 2000 nm. While it is known that typically male mice have thicker long bone cortex than females,^84^ we didn’t systematically analyze this aspect. On the other side, water absorption due to rovibronic transitions, especially in soft tissues such as the bone marrow, becomes relevant for power loss above 900 nm. Thus, for optimum 3P imaging of long bones, the appropriate excitation wavelength, which balances scattering and absorption, still needs to be found. Moreover, the impact of wavefront distortions caused by the tissue itself rises in 3p.m. with increased signal detection in deep tissue layers, calling for the implementation of wavefront correction using adaptive optics. As wavefront sensing in tissue is challenging, especially senseless wavefront correction methods are needed. In the present study we did not perform wavefront correction, as existing technologies^85^ need to be adapted to long bone imaging.

The photon flux density at the focal point, which determines the imaging depth in tissue, depends not only on the point spread function and laser pulse energy but also on the laser pulse shape and width. Femtosecond pulses are dramatically broadened in highly dispersive materials, such as biological tissues,^86^ requiring pulse compression for dispersion correction adapted to each tissue type and depth. Due to complex, still pending developments, we have not implemented such adapted dispersion correction in our system yet.

Several photophysical processes were observed by 3p.m. takes place on the scale of femtoseconds to a few nanoseconds, e.g., fluorescence and higher-harmonics generation processes. The low laser repetition rates, are needed to generate high pulse energy at 3p.m., lead to time periods between consecutive pulses of hundreds of nanoseconds to microseconds. Thus, for the above mentioned photophysical processes, the detectors collect noise, but no signal, over long periods of time. Therefore, detector time-gating is desirable,^32^ but it was not used in this study. As we plan to detect with our 3p.m. systems also photophysical processes on the microseconds scale, e.g., phosphorescence, we need to use dynamically adaptable time-gating to fit the timescales of all photophysical processes, which still need to be developed and implemented.

## Resource availability

### Lead contact

Further information and requests for resources and information should be directed to and will be fulfilled by Raluca Niesner (raluca.niesner@fu-berlin.de).

### Materials availability

This study generated a combined two- and three-photon laser-scanning microscope by implementing a two-stage optical parametric amplifier prototype with variable laser repetition rate in a commercial two-photon laser scanning microscope.

### Data and code availability

• The data that support the findings (microscopy images in.tiff format) of this study are available on the data repository Zenodo. The DOI is listed in the key resources table.
• The customized code for SIMI analysis, rose plot generation and the trained N2V algorithm are publicly available on GitHub. The links are listed in the key resources table (KRT).
• Any additional information required to reanalyze the data reported in this article is available on request by contacting Raluca Niesner (raluca.niesner@fu-berlin.de).

## Acknowledgments

The research leading to these results has received funding from the 10.13039/501100001659Deutsche Forschungsgemeinschaft, Germany under grant CRC 1444, P14 to R.A.N. and A.E.H. and P09, P13 to G.D., under grant FOR 5560, P02 to R.A.N (NI1167/9-1) and A.E.H (HA5354/13-1), under grant HA5354/12-1 to A.E.H. and under grant 317850156 to A.H. Additionally, funding from the Einstein Research Foundation, Berlin under grant ESB-A-2019-559 to A.E.H. and R.A.N. A.B. receives funding from the PhD program of the Berlin University for Applied Sciences and Technology. The authors thank R. Uecker, P. Mex and G. Korus for excellent technical assistance. The authors thank D. Walgurski (Helmholtz-Zentrum Berlin) for support in performing electron microscopy studies.

## Author contributions

A.R. built, optimized, and characterized the 2p.m./3p.m. imaging system. A.R., A.F.F, R.G., R.A.N., R.L., and Y.C performed the intravital experiments and imaging experiments on explanted tissues. J.H. and V.A. helped to optimize the imaging system and S.D. and L.W. developed the Ytterbium-based OPA laser together with A.R and R.A.N. A.E.H. and C.U. provided expertise in immune dynamics, plasma cell biology and regarding all animal experiments. Y.C. provided expertise regarding ER staining in long bones. J.R. and A.E.H. designed and performed the immunofluorescence experiments concerning the possible tissue photodamage by 3p.m. R.K. trained the N2V deep-learning algorithm. R.K. and A.F.F. performed image denoising. G.D. provided key expertise concerning the quantification of bone surface damage at 3p.m. A.B. and A.H. performed the Nanofocus-CT experiments. A.B. and I.B. performed the scanning electron microscopy experiments. A.R., A.F.F., A.E.H., and R.A.N. analyzed data, processed the results, and prepared the figures. A.R., A.F.F., A.E.H., and R.A.N. wrote the article. A.E.H. and R.A.N. supervised the study. All authors edited the article.

## Declaration of interests

S. Domingue and L. Wooldridge are with Thorlabs LASER Division CO, US. J. Heidelin and V. Andresen are with Miltenyi Biotec GmbH, Bergisch Gladbach, Germany.

## STAR★Methods

### Key resources table

**Table:** 

REAGENT or RESOURCE	SOURCE	IDENTIFIER
**Antibodies**		

ER Tracker^TM^ Red	Thermo Fisher Scientific	E34250
MitoTracker^TM^ Deep Red FM	Thermo Fisher Scientific	M46753
Ly6G (PE-conjugated)	Biolegend	164503; RRID: AB_2904311
CD68 (AF647-conjugated, FA-11)	Biolegend	137001; RRID: AB_2044003
CD45R (B220) (FITC-conjugated, anti-mouse REAfinity)	Miltenyi Biotec	130-110-845; RRID: AB_2658273
CD3 antibody (FITC-conjugated)	Biolegend	100203; RRID: AB_312660
HSP70 (unconjugated)	Cell signaling	4872; RRID: AB_2279841
anti-rabbit (AF647-conjugated)	Thermo Fisher Scientific	A31573; RRID: AB_2536183
anti-digoxigenin-FITC	Abcam	ab420; RRID: AB_304362

**Chemicals, peptides, and recombinant proteins**		

lipopolysaccharide	Sigma-Aldrich	L2630
DAPI	Roche	10236276001
super cryoembedding medium (SCEM)	Section-Lab Co. Ltd.	R040606
FluoSpheres™ Carboxylate-Modified Microspheres	Thermo Fisher	F8810
Fluoromount mounting medium	Thermo Fisher Scientific	00-4958-02
O.C.T.™ Compound	Science Services	SA62550-01
Movat’s pentachrome staining	Abcam	ab245884
electron microscopy grade 2% paraformaldehyde	Electron Microscopy Sciences	50-980-493

**Critical commercial assays**		

murine B cell isolation kit	Miltenyi Biotec	130-090-862
ApopTag Fluorescein *in situ* apoptosis detection kit	Sigma-Aldrich	S7110

**Deposited data**		

Imaging data – tibia 2023/2024	Zenodo	https://doi.org/10.5281/zenodo.8383833
Imaging data – tibia 2022	Zenodo	https://doi.org/10.5281/zenodo.7464124

**Experimental models: organisms/strains**		

Mouse strain: CD19:tdRFP:Cd19tm1(cre) ki/wt Gt(ROSA)26Sortm1Hjf dfl/dfl	Crossed at DRFZ	MGI No: 1931143 (CD19), 3696099 (tdRFP)
Mouse strain: Prx1:tdRFP: Tg(Prrx1-cre)1Cjt tg/u Gt(ROSA)26Sortm1Hjf dfl/dfl	Crossed at DRFZ	MGI No: 2450929 (Prx1), 3696099 (tdRFP)
Mouse strain: Cdh5:tdTomato x Histone:GFP (Cdh5:tdTom): Tg(Cdh5-mTdt/H2B-EGFP)1RhA tg/u	Obtained from Prof. R. Adams and Dr. M. G. Bixel	MGI No: 3848982
Mouse strain: Blimp1:GFP: Prdm1tm1Nutt ki/wt	Crossed at DRFZ	MGI No: 3510704
Mouse strain: Blimp1:GFP x CD19:tdRFP: Prdm1tm1Nutt ki/wt Cd19tm1(cre) ki/wt Gt(ROSA)26Sortm1Hjf dfl/dfl	Crossed at DRFZ for this study	MGI No: 3510704 (Blimp1), 1931143 (CD19), 3696099 (tdRFP)
Mouse strain: C57/Bl6J	Jackson Labs	RRID:IMSR_JAX:000664

**Software and algorithms**		

SIMI analysis	GitHub	https://github.com/niesner-ra/SIMI
rose plot generation	GitHub	https://github.com/niesner-ra/Tracks
trained N2V algorithm	GitHub	https://github.com/niesner-ra/N2V_3PM
Origin Pro	OriginLab	https://www.originlab.com/
GraphPad Prism 7.0c	GraphPad Software Inc.	https://www.graphpad.com/scientific-software/
2.4.0-RTM	GE Sensing & Inspection Technologies	https://www.bakerhughes.com/waygate-technologies
Python version 3.9	Python	https://www.python.org/
FIJI	FIJI	https://fiji.sc/
Imaris version 9.7.2	Oxford Instruments	https://imaris.oxinst.com/

### Experimental model and study participant details

In this study, experimental mice (*Mus musculus*) and murine cells were used.

#### Mice

All animal experiments were conducted in accordance with the ARRIVE guidelines and approved by Landesamt für Gesundheit und Soziales, Berlin, Germany in accordance with institutional, state and federal guidelines (G0048/21). Adult female and male mice (12–24 weeks old) were randomly assigned to experimental groups, as it is known that male mice have thicker cortical bone than females. All selected mice exhibited healthy behavior and weight and were housed in a conventional pathogen-free SPF barrier facility with enriched and suitable cage spaces with drinking water and standard chow diet *ad libitum* as recommended by the authorities.

For *in vivo* experiments we imaged the right tibiae using (*n* = 12) CD19:tdRFP B lymphocyte fate mapping mice,^11^, (*n* = 2) Prx1:tdRFP mesenchymal stromal cells fate mapping mice^87^ and (*n* = 16) Cdh5:tdTomato x Histone:GFP (Cdh5:tdTom) fate mapping mice^69^^,^^88^ (in which tdTomato is expressed in the membrane and GFP in the nuclei of endothelial cells) (kind gift of Prof. R. Adams, Dr. M.G. Bixel). For all comparative studies, we included at least 3 individuals per group, which were chosen arbitrarily, including both males and females.

We imaged *ex vivo* explanted tibiae from (*n* = 3) aged Cdh5:tdTom mice (one 106 weeks male and two 127 weeks old females), (*n* = 6) Blimp1:GFP mice, (*n* = 1) Blimp1:GFP x CD19:tdRFP mice and (*n* = 15) C57/Bl6J (Jackson Laboratories) mice. All specific genotypes are described in the key resource table.

#### Murine cells

For *in vitro* experiments, primary splenocytes from spleens of CD19 fluorescent mice were isolated using the Miltenyi murine B cell isolation kit (Miltenyi Biotec, Bergisch Gladbach, Germany) via magnetic-assisted cell sorting (MACS) and cultured in cell culture flasks in incubator with 37°C and 5% CO2. The cell purity was assessed by flow cytometry and the cell cultures were tested to ensure no mycoplasma contamination.

#### Human subjects

No human subjects or samples thereof were used in the present study.

### Method details

#### Setup for multi-photon laser-scanning microscopy

##### Excitation sources

The excitation sources for combined two- and three-photon microscope are two optical parametric amplifiers (OPA), using chirped laser pulses to support high pulse energies: (1) OPA design with tunable repetition rate (Ytterbia OPA), Thorlabs Inc. Lafayette CO, USA, with a Ytterbium-fiber based pump laser, 1030 nm, 20 W and (2) tunable AVUS OPA (APE GmbH, Berlin, Germany) pumped by Aeropulse FS20 (NKT Photonics, Birkerod, Denmark). Pulse compression was achieved using pre-chirp ZnSe plates (for Ytterbia OPA) and a fixed design of a two-prism pulse compressor (for AVUS OPA). The AVUS OPA systems operates in the wavelength range from 1200 nm to 2500 nm (idler), at fixed repetition rate of 2 MHz. The AVUS provides an output average power at 1330 nm of 700 mW (350 nJ per pulse at 2 MHz repetition rate) and at 1630 nm of 500 mW (250 nJ per pulse at 2 MHz repetition rate). We measured the pulse duration both directly at the laser output and under the objective, by second-order interferometric autocorrelation using CARPE (coating for spectral range 1200 nm–1700 nm, APE GmbH, Berlin, Germany). The combination of λ/2 waveplate and beam-splitter cube polarizer is used to control laser power. The excitation sources for state-of-the-art two-photon microscopy are a mode-locked Titanium-Sapphire laser (Ti:Sa, 690-1080nm, 80 MHz Chameleon Ultra II, Coherent, Glasgow, UK) tuned at 930 nm and an optical parametric oscillator (OPO, 1050–1350 nm, 80 MHz, APE GmbH, Berlin, Germany, pumped by the Ti:Sa) tuned either at 1100 nm or at 1330 nm. A high-accuracy positioning mirror is used to switch between the OPA and OPO laser beams. The Ti:Sa and optical parametric beams are combined by a dichroic mirror and overlapped in the microscope scan head.

##### Ytterbia OPA

The Ytterbia OPA (simplified design shown in Figure S1) uses an integrated Ytterbium fiber laser emitting at 1030 nm as pump laser, operating at fixed pulse energy. The system converts 190 fs pulses at 1030 nm into >65 fs pulses at 1650 nm by using white-light continuum generation in bulk media and two-stage optical parametric amplification. The ytterbium fiber pump laser operates using an oscillator, pulse picker and a chirped pulse amplifier to generate the 190 fs pulses with a pulse energy of >3.5 μJ and an average power of up to 20 W. The pulse-picking system is based on frequency division of the oscillator, which runs at 56 ± 2 MHz. The attainable repetition rates from 1 to 11 MHz are given by this oscillator frequency divided by an integer from 5 to 56, i.e., the value of the oscillator frequency.

The OPA is coherently seeded from white-light continuum generated in bulk media by the ytterbium fiber laser pulse train (Figure S1A). In the first stage, the pump pulse and the white-light seed are brought to a common focus within a nonlinear crystal (PPLN) amplifying the infrared signal at 1650 nm. During the second stage, the outcoming pump pulse and the signal pulse trains are again focused into another nonlinear crystal for further amplification. The power spectral bandwidth of the output signal supports <65 fs pulses and contains 500 nJ of energy at repetition rates of 1–4 MHz. Specifically, the emission wavelength of the Ytterbia OPA is fixed at 1650 nm, bandwidth 60 nm, with tunable repetition rate between 1.01 MHz and 3.98 MHz, providing an average output power of 1.8 W (452 nJ) at 3.98 MHz, 1.49 W (481 nJ) 3.09 MHz, 1 W (486 nJ) at 2.06 MHz and 0.5 W (495 nJ) at 1.01 MHz.

Pulse intensity profiles retrieved from SHG-FROG are numerically scaled by the measured pulse energies and shown in Figure S1B. The deviation from the average maximum peak power of 16.1 MW is <1.5%. The white light seed has a nearly singular input pulse for stable filamentation and spectral broadening, for the given wavelength, duration, and spot size. As the 1030 nm laser pulses are invariant with repetition rate, so is the power ratio between white-light and OPA-pumping in both optical arms. Additionally, the short duration (190 fs FWHM) and high temporal quality (0.95 temporal Strehl or 95% of the transform-limited peak power) of the 1030 nm laser pulses allow for white light generation at low energy levels, i.e., ∼1 μJ, thus, enabling efficient pumping of the OPA even at low total input energy levels, <5 μJ. Following the similarity in input, both 1030 nm pump and 1650 nm output OPA pulse trains behave similar, as shown in the retrieved SHG-FROGs of the OPA signal output post compression (Figure S1). The intensity profiles are scaled by the measured pulse energy to convert to units of MW of peak power. The small variation in maximum peak power is due to small changes in the beam pointing due to the large changes in thermal load at different repetition rates.

The compact design of the Ytterbia OPA laser is achieved by vertically coupling the ytterbium fiber pump laser and the OPA within a single housing. This eliminates the need for beam routing on the optical table, which creates an optical system that is less sensitive to environmental changes. The front panel of the Ytterbia OPA features a main output for the OPA signal, as well as a bypass output that provides full access to analyze the pump beam.

##### Microscope setup

The laser-scanning microscope used in this study is based on a commercial two-photon microscopy system (TriMScope II, LaVision BioTec, Bielefeld, Germany – now Milteniyi Biotec, Bergisch-Gladbach, Germany),^59^ in which we replaced the optics for broad spectral transmission up to 1700 nm, matching the polarization of all excitation sources. Water-immersion objective lenses (Olympus, XLPLN25XWMP2, 25x, NA 1.05; Nikon, CFI75 Apo 25XC W 1300, 25x, NA 1.1, Tokyo, Japan) are used to focus the excitation laser beams into the sample. Fluorescence, SHG and THG signals are collected through the objective lenses in the epi-direction using a dichroic mirror (900, Chroma, US) and directed toward four photomultiplier tubes (PMTs, H7422-40/-50, Hamamatsu, Japan). All PMTs are assembled in a detection system with four optical channels, where each channel is defined by individual interference filters and a set of dichroic mirrors. The assignment of optical channels depends on the excitation wavelength. The optical filters for excitation at 930 nm (Ti:Sa) are 447 ± 20 nm for SHG, 525 ± 25 nm for the GFP signal, 595 ± 25 nm for the tdRFP and tdTomato signals, at 1100 nm (OPO), 562 ± 20 nm for the SHG signal and 595 ± 25 nm for the tdRFP and tdTomato signals. At 1330 nm (OPA or OPO), following filters were used: 447 ± 20 nm for THG, 525 ± 25 nm for the GFP fluorescence, 595 ± 20 nm for the tdTomato or ER tracker fluorescence and 655 ± 20 nm for the SHG signal. At 1630 nm/1650 nm (of both OPA systems), we used the following filters: 562 ± 20 nm for THG signal, 595 ± 20 nm for the tdTomato, tdRFP or ER tracker fluorescence and 810 ± 45nm for the SHG signal. Acquisition time, pixel dwell time and z-step for the 3D z-stacks are stated in the Results and Figure legends. Time-lapse 2D image acquisition was performed at 1 Hz (every 1 s) and 2 Hz (every 0.5 s). Time-lapse 3D image acquisition was performed every 30 s over up to 1 h or every 120 s over 2 h.

#### *Ex vivo* NanoCT imaging

The explanted tibiae were placed in a hollow plastic cylinder filled with Styrofoam to prevent movement. The cylinder was glued to a glass rod and fixed in the sample holder of the nanofocus-CT system (Phoenix Nanotom M, GE Sensing & Inspection Technologies GmbH, Wunstorf, Germany). CT scans were acquired at a voltage of 100 kV, a current of 120 μA, and a 0.1 mm copper prefilter. One measurement included 1500 radiograms (3 scans per angular step for noise reduction, with 500 ms acquisition time per single scan), the total acquisition time being 54 min. The magnification was 50x, with a voxel edge length of 2.00 μm. The scans were reconstructed as 3D volumes using the phoenix datos|X-ray2 reconstruction 2.4.0-RTM software (GE Sensing & Inspection Technologies GmbH, Wunstorf, Germany).

#### Scanning electron microscopy

The explanted tibiae were fixed in 1% paraformaldehyde (PFA) and dehydrated in a series of exchanges with increasing concentrations of ethanol (99.8%) diluted in distilled water (50%, 70%, 80%, 90%, 95%) for 4 min each. Finally, they were kept in 100% concentration ethanol for 20 min, followed by 5 rinses in liquid carbon dioxide, at the critical draying point of CO_2_ (31.1°C at 73 atm). The dehydrated specimens were stored in phosphate buffer solution (PBS). The samples were fixed with conductive adhesive on sample holders and sputtered with 25 nm platinum. Imaging was performed with a Zeiss ULTRA Plus scanning electron microscope (Carl Zeiss NTS GmbH, Jena, Germany) at an electron high tension of 5.00 kV, with an aperture size of 30.00 μm and a working distance of 11.2 mm–13.2 mm. Magnification in the shown imaged was in a range of 24x to 26x.

#### Image processing and analysis

##### Similarity unmixing (SIMI) of multi-photon images

We previously developed similarity unmixing algorithm (SIMI), a numerical pixel-based algorithm, to resolve mixtures of cellular and tissue compartments labeled by different fluorophores or non-linear harmonic generation signals, such as SHG and THG, using a low number of detection channels.^59^^,^^64^ In the conventional SIMI pipeline, fingerprints of individual cellular and tissue compartments are determined from separate single-color measurements. In the multicolor 3D images acquired in the tibia of Blimp1:GFP mice after ER staining at 1330 nm, we expected overlapping signals in single elementary voxels, i.e., THG signal, GFP fluorescence and fluorescence of endoplasmic reticulum (ER) staining may appear together within one voxel. Thus, we needed to extend the initial SIMI algorithm and to identify *in situ* fingerprints in the analyzed multiplexed images, generated using four detection channels (447 ± 20 nm, 525 ± 25 nm, 595 ± 20 nm and 655 ± 20 nm). We found and assigned fingerprints to three mixed components, THG+ER (bone marrow), THG+GFP+ER (bone marrow) and THG+SHG (bone cortex), and to two individual signals, GFP and THG (Figure S9). SIMI unmixing was performed based on similarity analysis, i.e., searching for the closest match between the pixel intensities of the raw image and the signal intensity of the determined fingerprint for each detection channel.

##### Noise2Void image denoising

To improve SNR by reducing the noise level in the time-lapse 3D images of tdRFP^+^ B lineage cells in the tibial bone marrow, we used a machine-learning algorithm, Noise2Void (N2V)^61^ plug-in as a part of the open-source neural network algorithms CSBDeep^89^ package in FIJI. The trainable deep-learning scheme of N2V plug-in allows to predict the original shape of the cells without clean targets. This was used to improve cell segmentation and tracking. The fluorescence signal in each pixel is restored by reducing the estimated noise, which distribution differs in a certain neighborhood, known as the receptive field. The training of the N2V-models was performed on individual 2D images from different time points and tissue depths, in different experiments, to enhance model generalization. To account for PMT properties, we performed training on each detection channel individually. The model setting was 20 images for each run, 200 epochs, 300 steps per epoch, 128/256 batch size per step, and neighborhood radius of 5.

#### Cell and tissue segmentation, cell tracking

##### Cell segmentation and classification

3D reconstruction of z-stacks and NanoCT images was performed in Imaris (software version 9.7.2, Bitplane, an Oxford Instruments company, Belfast, UK). Cell segmentation in multi-photon 3D stacks and time-lapse 3D images was automatically performed using Imaris, after machine-learning-based noise reduction using the trained N2V algorithm in FIJI, version 1.53t as previously described. Motion correction of the time-lapse 3D images in Video S7 was performed using the Fast4Dreg plugin^90^ in FIJI, before further processing the data. For object surface detection in Imaris, we used background subtraction relying on local contrast and separated touching objects, relying on seed point diameter of 7.5 μm. Co-localization with co-registered additional signals in both 3D stacks and time-lapse 3D images was performed using the filtering function in Imaris, after cells were segmented. The same function was used to classify segmented cells by volume, e.g., to distinguish between B and plasma cells in CD19:tdRFP mice.

##### Cell tracking

Automatized cell tracking in time-lapse 3D images was performed using Imaris, after cell segmentation. Therefore, we used the autoregressive motion algorithm in Imaris, with a maximum frame-to-frame distance of 8 μm and maximum number of gaps = 3. We verified all tracking results and, in some cases, disconnected tracks of one and the same cell were manually connected.

##### Cortical thickness

The cortical thickness of the tibia was determined by applying the FIJI function *Local Thickness* on the xz- or yz-resliced 3D images of SHG and THG signals in the tibia.

#### Surgery for intravital tibia imaging

The mice were placed on a heating plate at 37°C and anesthetized by inhalation narcosis with isoflurane, corresponding to their weight. The right hind paw was fixed, by stretching the leg to the side, and prepared for further surgery. To prevent contamination, the fur was shaved clean on the upper side of the fixed paw. In the diaphysis area of the tibia, the skin layer was dissected, and the muscle fibers were pushed aside to get access to the flat, medial surface of the tibia shaft. The tibia was kept in position by fixing the crest with a surgery clamp. A wall of 1% agarose gel was built around the opened area to form a cylindrical bath filled with isotonic NaCl solution for imaging. The medial part of the tibia was imaged, as indicated in Figure 1(iv). Alternatively, tibia cortex was additionally thinned with a diamond drill, for comparative imaging at low vs. high-pulse energy (2p.m. vs. 3p.m.).

#### Explanted tibia preparation for imaging

C57/Bl6J, Blimp1:GFP or Blimp1:GFPxCD19:tdRFP mice were sacrificed by cervical dislocation. Tibia bones were explanted and muscle and connective tissues removed, tibia was glued on a Petri dish, which was filled with PBS and immediately imaged.

#### Endoplasmic reticulum and mitochondria staining in explanted tibia

The freshly explanted tibiae were placed in a Petri dish containing ER Tracker Red (BODYPI TR Glibenclamide) and/or MitoTracker Deep Red FM for cell imaging (Invitrogen, Eugene, Oregon, USA) in PBS medium, at 37°C for 30 min. For that, 5 μL stock solution of ER Tracker Red (MW 915.23 g/mol) and/or of MitoTracker Deep Red FM (MW 543,58 g/mol) in DMSO (concentration 1 mmol/L) were diluted in 5 mL PBS. The epiphyses of the bones were removed for dye penetration. The tibiae were washed with PBS to remove exceeding dye and glued on a Petri dish, containing PBS, for imaging purposes.

#### Lipopolysaccharide and rapamycin stimulation of primary B cells

Primary splenocytes were isolated from spleens of CD19 fluorescent mice in 1× PBS and erythrocytes were lysed. B cells were negatively isolated using the Miltenyi murine B cell isolation kit (Miltenyi Biotec, Bergisch Gladbach, Germany) via magnetic-assisted cell sorting (MACS), leaving B cells untouched to avoid pre-stimulation. The B cells were seeded in cell culture flasks and stimulated by lipopolysaccharide from *Escherichia coli* (LPS, Sigma) for 48 h, to generate antibody secreting LPS blasts, which were imaged by 3p.m. A portion of the LPS blasts were treated with 100 nmol/L rapamycin 12 h prior to imaging. The cell supernatant was analyzed using enzyme-linked immunosorbent assay (ELISA).

#### Tissue preparation for histology

Freshly explanted tibiae and spleens were fixed in 4% PFA for 4 h, cryoprotected in 10–30% sucrose/PBS, embedded in O.C.T. and stored at −80°C. 7-micrometer-thick serial spleen sections were cut using a cryostat and collected on positively charged slides. Fixed tibiae were covered with Kawamoto’s medium (SCEM, Section-Lab Co. Ltd., Hiroshima, Japan), frozen and cryo-sectioned using the Kawamoto’s film method.^91^ Sections were kept at −80°C until further use.

#### Immunohistochemistry analysis

For immunofluorescence, slides were dried, washed in PBS, blocked with 10% serum, and stained with antibodies in PBS, containing 1% serum and DAPI, for 1–2 h. The following antibodies were used: Ly6G (PE-conjugated), CD68 (AF647-conjugated, FA-11, Biolegend), CD45R (B220) antibody (FITC-conjugated, anti-mouse REAfinity, Miltenyi GmbH, Bergisch Gladbach, Germany), CD3 antibody (FITC-conjugated, Biolegend), HSP70 (unconjugated, 4872, Cell signaling), anti-rabbit (AF647-conjugated). Stained slides were mounted with Fluoromount mounting medium (Thermo Fisher, MA, US). TUNEL staining was performed using an ApopTag Fluorescein *in situ* apoptosis detection kit (S7110, EMD Millipore, Germany) according to the manufacturer’s protocol. Briefly, the O.C.T.-embedded sections were thawed and rehydrated, and then permeabilized in cooled acetic acid and in ethanol for 3 min at −20°C. Sections were incubated with the reaction buffer containing TdT enzyme at 37°C for 1 h. After washing with stop/wash buffer, sections were treated with anti-digoxigenin-FITC for 30 min at room temperature. The sections were counterstained with DAPI before being mounted with Fluoromount mounting medium. Alternatively, 7 μm tibia sections were stained using the Movat’s pentachrome staining.^92^ Both immunofluorescence and Movat’s pentachrome staining histological analysis was performed on a fluorescence wide-field microscope (Keyence BZ800, Keyence Deutschland GmbH, Neu Isenburg, Germany), using magnifications of 4x, 10x, and 20x.

#### Effective point-spread function measurements

The spatial resolution of our imaging setup was determined as the lateral and axial dimensions of the effective point spread function (ePSF), measured relying on the highly resolved 3D fluorescence signal of 200 nm size nanospheres (605 nm emission wavelength, F8810 FluoSpheres, Invitrogen, Eugene, Oregon, USA), embedded in agarose gel (1%). The nanosphere are used as a standard for effective PSF measurements, as their size is below the resolution limit of the imaging setup. Following the Nyquist criterion, the voxel size for the PSF measurements was 0.1 μm laterally (xy) and 0.5 μm axially (z). x/y and z fluorescence intensity profiles of the nanospheres were plotted and approximated by Gaussian functions (Origin2023, OriginLab) to determine the dimension of the ePSF in the respective direction.

### Quantification and statistical analysis

Statistical details (used statistical procedures and tests, definition and number of *n* values, statistical metrics such as *p* values, definition of significance and error metrics) can be found in the figure legends. Figure 2E displays mean and standard deviation (s.d.) values for *n* = 11 mice with intact tibia and *n* = 5 mice with mechanically thinned tibia. Figure 2F shows representative linear regression performed in Origin 2023 for the double-logarithmic representation of fluorescence signal as a function of imaging depth z, to determine the effective attenuation length of radiation *l*_*e*_ at 1100 nm, 1330 nm and 1650 nm Figure 2G shows *l*_*e*_ values in tibia cortex and marrow, in *n* = 4 mice at 1100 nm, *n* = 3 mice at 1330 nm and *n* = 8 mice at 1650 nm. Mean and s.d. values are displayed for each group and two-way ANOVA statistical analysis with Bonferroni post-test was performed. Figures 3C and 3D shows the lateral and axial dimensions of sub-resolution THG structures (or the results of the first derivative) in different depth, for 1650 nm and 1330 nm. Each data point represents the mean of *n* = 5 different structures in each tissue depth, with the s.d. values displayed. The diffraction limit was calculated using the Debye vectorial approximation. Figure 4B (left panel) shows the SNR values for each tissue depth as mean value and with s.d. for all pixels of the image (518x518 pixel) with signal count above background defined at sites outside of the tissue, for 1, 2, 3 and 4 MHz repetition rate. Figure 4B (right panel) shows the laser pulse energy at the tibia surface and the effective pulse energy in tissue, calculated considering the determined *l*_*e*_ values in bone cortex and marrow. Figure 5C displays the mean values from *n* = 6 mice with 3p.m. exposed tibia (mean value for 3 ROI each) and contralateral (unexposed) tibia from 3 of the 6 mice. s.d. values are displayed, with Student’s t test performed for statistical analysis. Figures 5I and 5J (left graphs) show the cell volume for B lineage cells, with *n* = 1278 at 1100 nm (*n* = 1121 B cells, *n* = 56 plasma cells), and *n* = 2142 (*n* = 2006 B cells, *n* = 136 plasma cells) at 1650 nm Figures 5I and 5J (right graphs) shows the displacement rates of these cells, with mean values and s.d. displayed. For statistical analysis we used Student’s t test, with *p* values shown. Figure 6E shows the displacement rate of *n* = 64 plasma cells, with mean and s.d. values, as a dot plot (left) and as a histogram (right), with double-peak Gaussian fit (Origin 2023), for a representative mouse. Figure 6F shows for *n* = 3 mice the results of the double-peak Gaussian fit, to distinguish between non-migratory and migratory plasma cells, with mean and s.d. values. Figure 6H shows the THG^+^ frequency in B compared to plasma cells, in *n* = 6 mice, with at least 500 B lineage cells per each mouse. Mean and s.d. values are displayed and statistical analysis was performed using Student’s t test, with *p* value indicated. Figure 7D shows the percentage of THG^hi^ ER^+^ cells compared to all ER^+^ and to THG^+^ cells, respectively for *n* = 10 mice, with at least 300 analyzed cells per mouse. Figure 7G shows the percentage of THG^hi^ cells among B cells and plasma cells in CD19:tdRFP mice (*n* = 6 mice, with at least 300 B lineage cells per mouse) as well as THG^hi^ and THG^hi^ ER^+^ among plasma cells in Blimp1-GFP mice (*n* = 5 mice, with at least 50 cells per mouse), with mean and s.d. values per group displayed. For statistical analysis we used two-way ANOVA with Bonferroni post-test, *p* value ranges being. indicated. Figure 7H shows the displacement rates of THG^hi^ (*n* = 45 cells) vs. THG^lo^ plasma cells (*n* = 112 cells), with mean and s.d. values indicated from one representative mouse. Statistical analysis succeeded using the student t-text, with *p* value range indicated. Figure 7I shows the same information as Figure 7H as histograms for the two plasma cell populations, to highlight the bimodal distribution of displacement rates of THG^lo^ plasma cells as compared to the monomodal distribution of the THG^hi^ plasma cells. Statistical analysis was performed using Origin 2023 (OriginLab, CA, US) and GraphPad Prism 7.0c (GraphPad Software Inc., San Diego, CA, US), for validation. If *p*-values are not shown, *p* > 0.05 non-significant is not shown, ∗ stays for *p* < 0.05, ∗∗ for *p* < 0.01, ∗∗∗ for *p* < 0.001.

## Supplemental information

Document S1. Figures S1–S10 and Tables S1–S3

Data S1. Theoretical background of three- and two-photon-excitation
