# Supplementary material for: Optimized intravital three-photon imaging of intact mouse tibia links plasma cell motility to functional states
Source: iScience. 2024 Sep 17;27(10):110985. doi: 10.1016/j.isci.2024.110985 (PMC11466647; doi:10.1016/j.isci.2024.110985)
Supplement: Document S1. Figures S1–S10 and Tables S1–S3 [file mmc1.pdf]

iScience, Volume *27*

Supplemental information

Optimized intravital three-photon imaging of intact mouse tibia links plasma cell motility to functional states

Asylkhan Rakhymzhan, Alexander F. Fiedler, Robert Günther, Scott R. Domingue, Laura Wooldridge, Ruth Leben, Yu Cao, Anne Bias, Jay Roodselaar, Ralf Köhler, Carolin Ulbricht, Judith Heidelin, Volker Andresen, Ingeborg Beckers, Astrid Haibel, Georg Duda, Anja E. Hauser, and Raluca A. Niesner

Supplemental Figures

Supplemental Figure 1 related to Figure 1

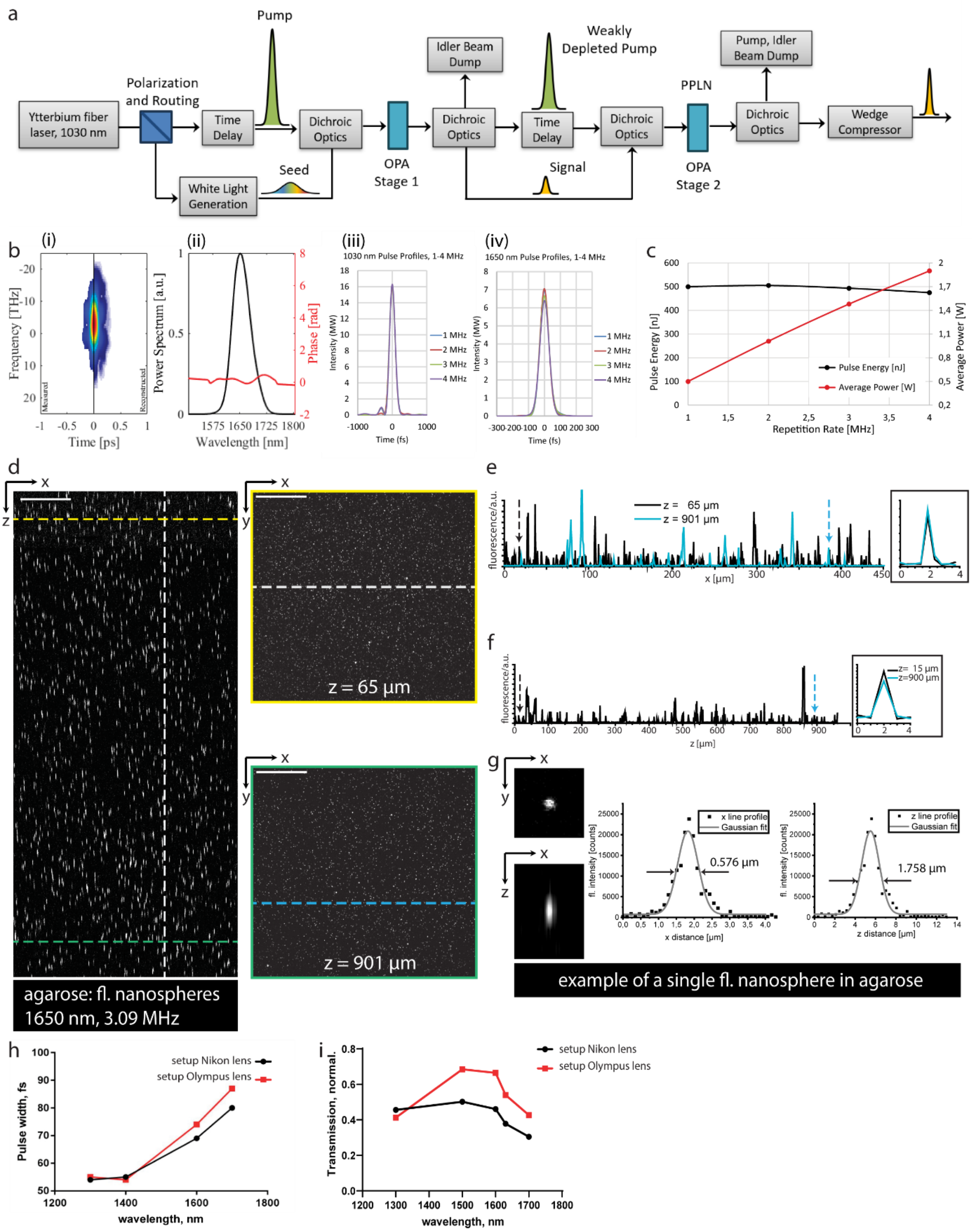

Characterization of the optical parametric amplifier with tunable repetition rate and of the multi-photon microscope setup used for three-photon imaging of intact murine tibia. a. Design of Ytterbia OPA. The laser is a two-stage, collinear OPA

with recycled pumping and seeding by bulk derived white light generation. Unshown in the block diagram are mode matching optics for both amplification stages, to optimize conversion efficiency while preserving beam quality. **b**. The second harmonic frequency resolved optical gating characterization of the Ytterbia optical parametric amplifier (OPA) pulse. (i) The Phantom-FROG trace of the 1650 nm pulse, with the measured trace on the left and the reconstructed trace on the right side of the graph. (ii) The reconstructed power spectrum (black) and phase (red). The reconstructed intensity profile, scaled to the normalized transform limited intensity profile of the power spectrum for all repetition rates (1-4 MHz) of the pump 1030 nm laser (iii) and the signal at 1650 nm (iv). **c**. The pulse energy (black) and average power (red) scaling of the Ytterbia OPA as a function the repetition rate. We ensured that the selected optical elements of the microscope setup and their anti-reflex coatings are adequate for the extended infrared wavelength range, resulting into a total transmittance of the microscope system at 1650 nm of 28.3±0.4 %. To compensate the slightly positive group-velocity dispersion (GVD) of our setup, we integrated ZnSe windows[1] of variable thickness (for Ytterbia OPA). We found that a total thickness of 13.5 mm ZnSe provides the narrowest pulse width under the objective lens. The optimum ZnSe thickness and the performance of the pulse compressor were assessed by measuring the pulse width by second-order interferometric autocorrelation (CARPE, APE, Berlin, Germany) under the objective lens. **d**. xz (442x1001 μm^2, 1036x1001 pixel) and corresponding xy (442x442 μm^2, 1036x1036 pixel) fluorescence images of 100 nm fluorescent nanospheres embedded in agarose (emission at 605 nm), upon excitation at 1650 nm OPA radiation, at 3.09 MHz repetition rate, 0.8 nJ pulse energy. The horizontal yellow line in the xz fluorescence image shows the z-position (65 µm) of the yellow-framed xy image, the horizontal green line, the z-position (901 µm) of the green-framed xy image. The vertical white line in the xz-image shows the x-position of the fluorescence signal profile in e. The horizontal white line in the yellow-framed xy image shows the black profile in d, the cyan line in the green-frame xy image, the cyan profile also in e. An example of xy and xz projection of a fluorescent nanosphere, with x- and z-line profiles, together with Gaussian fits are shown. Thus, we found a homogeneous illumination of the entire field of view (FOV), over more than 1 mm depth. Similar lateral (xy) and axial (z) effective PSF line profiles, in different regions within the full FOV and at different imaging depths indicate negligible or no wave front distortions or loss of spatial resolution, caused by the optical elements. **e**. Line profiles along x, as indicated in d, showing similar signal strength along the x-axis. The black arrow indicates the x-profile of a single sphere in 65 µm

depth, the cyan arrow the x-profile of a single sphere in 901 µm depth. These two profiles are displayed in the inset of e, showing no loss of lateral resolution with depth. **f**. Line profile along z, as indicated in d, showing also axially similar signal strength. The black and cyan arrows indicate the z-profiles of single nanospheres, in 15 and 901 µm depth, respectively, which are displayed in the inset of e, showing also no loss of axial resolution with depth. The measured effective PSF dimensions of the nanospheres are in good agreement with values calculated by the (Debye) vectorial approximation[2,3] (558.6 nm, 1.948 µm). **g**. Dependence of pulse width of the tunable OPA laser on the wavelength, measured under two different objective lenses (Olympus, XLPLN25XWMP2, 25x, NA 1.05 and Nikon, CFI75 Apo 25XC W 1300, 25x, NA 1.1), which were designed for high transmission in the infrared range. We found similar values for both lenses, increasing from 55 fs up to 90 fs between 1300 and 1700 nm, using a two-prism pulse compressor to counteract the GVD of the microscope setup. **h**. Transmission of the setup using the same two objective lenses, in the wavelength range 1300 nm – 1700 nm. Scale bar = 100 µm in all images. The higher transmission at longer wavelengths (between 1600 and 1700 nm) was decisive for the use of the XLPLN25XWMP2 objective in all further experiments. The back aperture, for both objective lenses, was overfilled by a factor of 1.5 to achieve best spatial resolution.

Supplemental Figure 2 related to Figure 2

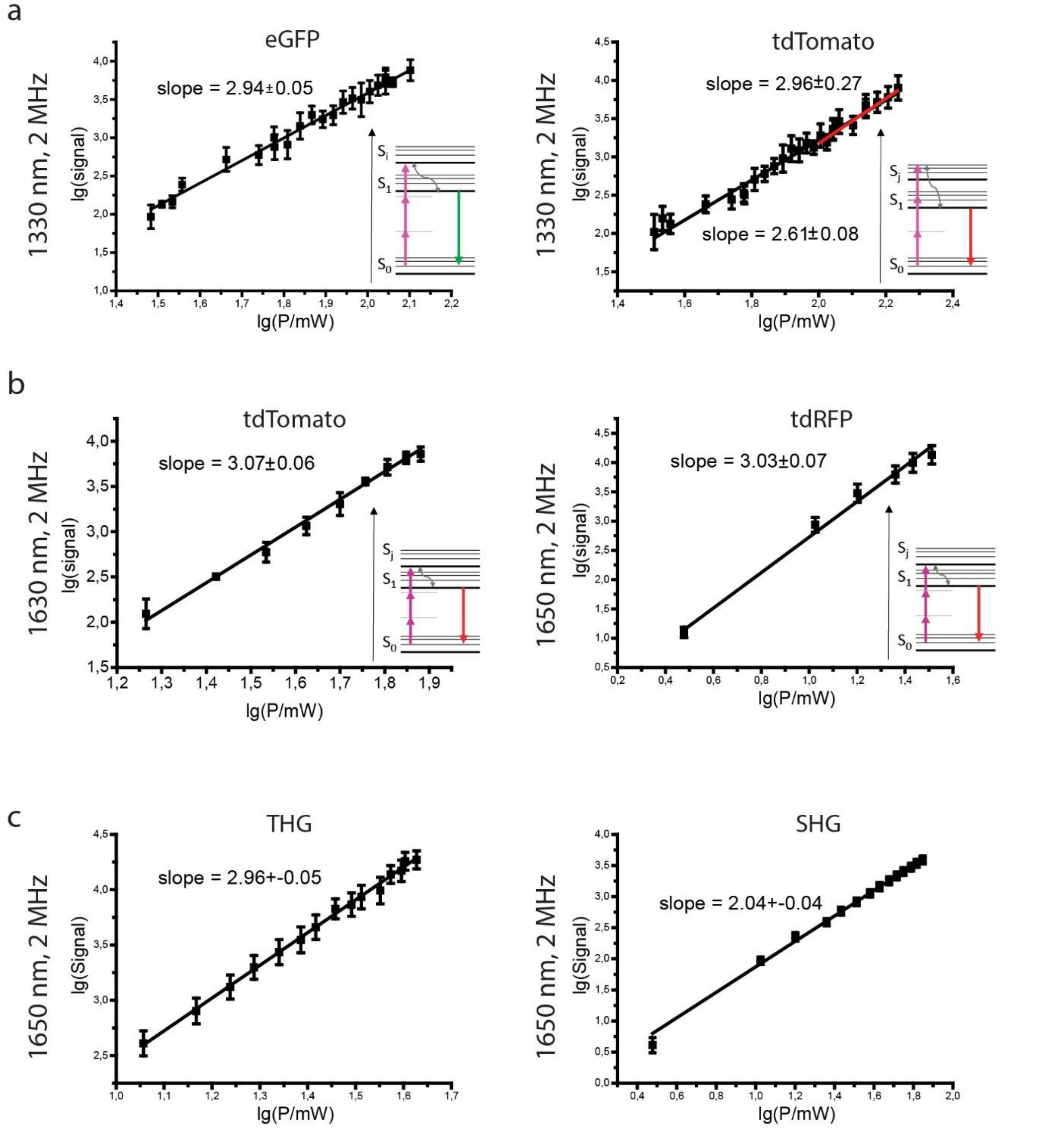

Wavelength dependence of the optically non-linear processes leading to fluorescence or higher harmonics generation. a. Dependence of GFP and tdTomato fluorescence signal, respectively, on the average power of the OPA laser tuned at 1330 nm, in double-logarithmic representation. The slope of the linear approximation indicates the type of excitation, preceding spontaneous emission: a slope of 3 hints to a non-resonant three-photon excitation, a slope below 3, approaching 3 only at high powers, hints towards a resonance-enhanced three-photon excitation. Schematics of the energetic molecular levels and transitions are displayed

as insets in graphs. At 1330 nm, we found for GFP fluorescence a slope of ≈3, complying with a non-resonant three-photon excitation, whereas for tdTomato fluorescence the dependence on the average laser power was not linear, with a slope <3 for low laser powers and ≈3 for high laser powers. This indicates a resonance-enhanced three-photon excitation, i.e. a two-step 2+1 photon excitation, in agreement with previously published work[4]. **b**. As in **a**, dependence of tdTomato and tdRFP fluorescence signals on the laser average power at 1630 nm or at 1650 nm. In both cases, the slope of 3 indicates a non-resonant three-photon excitation, as shown also by the schematics of the energy diagram, as graph insets. At 1650 nm, we found slopes of ≈3 for both tdTomato and tdRFP fluorescence, indicating non-resonant three-photon excitation processes. The three-photon absorption cross-sections $\eta_3 \cdot \sigma_3$ of tdRFP and tdTomato in B cells at 1650 nm were estimated from their known two-photon absorption cross-sections $\eta_2 \cdot \sigma_2$ at 1100 nm, i.e. for tdRFP $20.2 \cdot 10^{-50}$ $cm^4 \cdot s$ (20.2 GM)[6] and for tdTomato $80 \cdot 10^{-50}$ $cm^4 \cdot s$ (80 GM)[7], to be $15.85 \pm 0.2 \cdot 10^{-83}$ $cm^6 \cdot s^2$ and $21.64 \pm 1.35 \cdot 10^{-83}$ $cm^6 \cdot s^2$, respectively. For tdTomato, the $\eta_3 \cdot \sigma_3$ value has the same order of magnitude as previously reported σ_3[4]. **c**. Dependence of second harmonics generation (SHG) and third harmonics generation (THG) on the average laser power, at 1650 nm, 2.06 MHz (Ytterbia OPA). As expected, we found a slope of ≈2 for the SHG signal (in the collagen fibers of bone cortex) and of ≈3 for the THG signal (in lacunae and canaliculi of the bone cortex), at 1650 nm.

Supplemental Figure 3 related to Figure 2

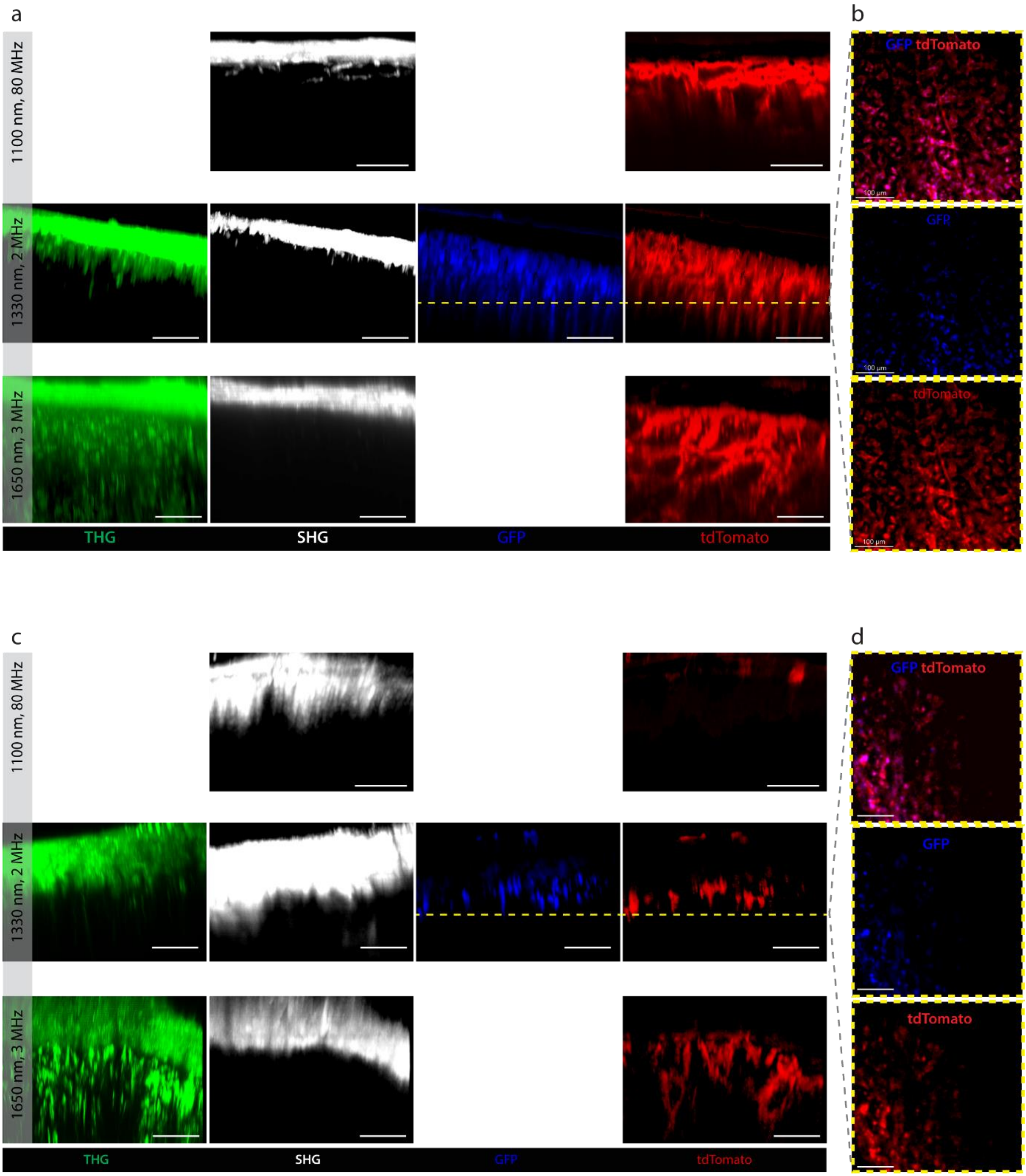

Single channel images in the tibia of adult mice, either with mechanically thinned or intact bone cortex, corresponding to the merged images in Figure 2. a. Single channel z projections of 3D images acquired by 3PM at 1650 nm, 3 MHz or at 1330 nm, 2 MHz and by 2PM at 1100 nm, 80 MHz in the tibia of Cdh5:tdTomato x Histone:GFP mice, with mechanically thinned cortical bone. SHG signal is shown gray, THG signal in green, tdTomato fluorescence in the endothelial cell membranes in red and GFP fluorescence in the endothelial cell nuclei in blue. **b**. Single channel and

merged xy fluorescence images acquired at 1330 nm, 2 MHz in 200 µm in the tibia marrow. The z-position in tissue is indicated by the yellow dashed line in a. Single channel images show GFP fluorescence of endothelial cell nuclei in blue and tdTomato fluorescence of endothelial cell membranes in red. **c**. Single channel z projections of 3D images acquired by 3PM at 1650 nm, 3 MHz or at 1330 nm, 2 MHz and by 2PM at 1100 nm, 80 MHz in the intact tibia of Cdh5:tdTomato x Histone:GFP mice. Notable, the maximum laser repetition rate in tissue may also be limited by the saturation energy of tdTomato (4.03 nJ) or tdRFP (4.47 nJ), values calculated at 1650 nm from the three-photon absorption cross-section $\eta_3 \cdot \sigma_3$ values, and by that, the maximum average power at the surface of the specimen[9,10]. Assuming 150 µm cortical thickness and 350 µm imaging depth in the bone marrow, the maximum repetition rate supported by an average power of 500 mW at the surface of the bone cortex is 7.83 MHz for tdRFP and 18.74 MHz for tdTomato, both higher than 3.98 MHz of Ytterbia OPA. **d.** Single channel xy GFP and tdTomato fluorescence images of endothelial cells acquired at 1330 nm, 2 MHz in 180 µm in the tibia marrow, indicated by the dashed yellow line in c. Color coding as in **a** and **b**. Scale bar =100 µm.

Supplemental Figure 4 related to Figure 2

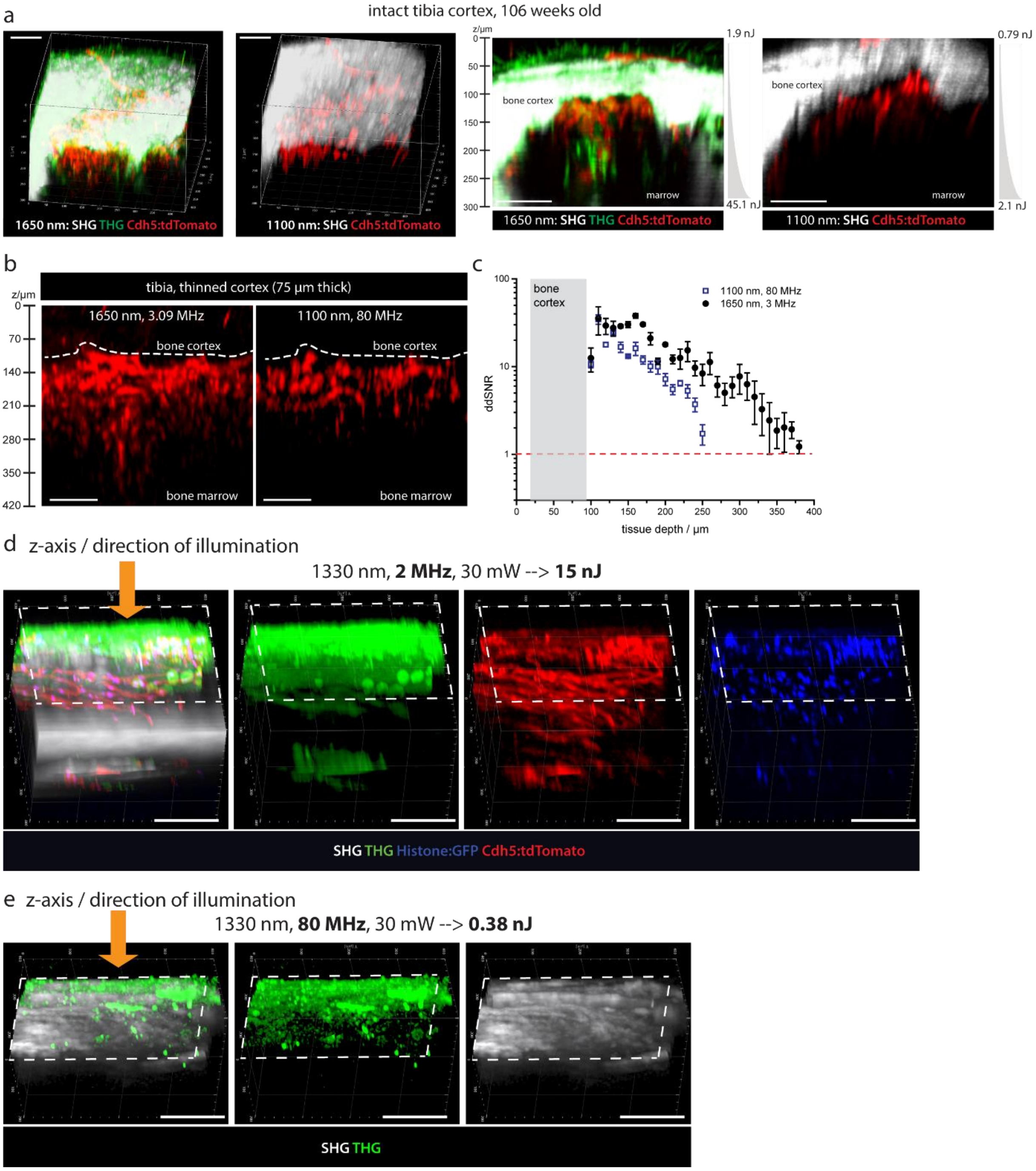

Both high-pulse energy and long wavelength excitation are required to maximize imaging depth in murine tibia. a. 3D reconstructions (left side) and xz projections over 50 µm along the y axis (right side) of SHG (gray), THG (green) and tdTomato fluorescence (red) in blood vessels of a Cdh5:tdTomato x Histone:GFP mouse (106 weeks old, with locally thin bone cortex), upon excitation at 1650 nm, 3.09 MHz, and at 1100 nm, 80 MHz, respectively. For both excitation schemes exponential z-adaptation of laser power was used as indicated. We succeeded to image tdTomato

fluorescence in blood vessels, in the marrow tissue underneath the intact bone cortex at both 1650 nm and 1100 nm, as the cortical bone of frail female mice is locally thinner than in adult mice[8]. These observation was confirmed in the explanted tibia bones of two further 127 weeks old Cdh5:tdTom mice. **b**. xz projections over 50 µm in y-direction (400x400x420 µm³, 518x518x160 voxel) of tdTomato fluorescence in blood vessels in the tibia of a Cdh5:tdTomato x Histone:GFP mouse (12 weeks), with mechanically thinned cortex (75 µm thick), upon excitation at 1650 nm, 3.09 MHz and 1100 nm, 80 MHz, without z-adaptation of power. **c**. Depth dependence of SNR values calculated for the tdTomato fluorescence, in images shown in b. The ddSNR measured upon 1100 nm excitation declines to a value of 1 in ≈160 µm depth in the tibia marrow, measured from the interface between bone cortex and bone marrow. In contrast, upon three-photon excitation at 1650 nm (3 MHz, Ytterbia OPA) the ddSNR reaches the value 1 in ≈300 µm depth in the tibia marrow, being by a factor of ≈1.9 larger. For comparability reasons, we adjusted the average laser power of Ytterbia OPA and OPO to achieve the same SNR of tdTomato fluorescence using both excitation schemes at the interface between bone cortex and bone marrow. We performed imaging without z-adaptation of power, at the same pixel dwell time (1.98 µs, 2x frame acquisition. Thus, we achieved a lower penetration depth in the tibia as compared to the case in which we used z-adaptation of laser power, in the intact tibia (Fig. 4a). We could not compare the ddSNR using the two excitation schemes in the intact tibia of adult mice, because imaging through the thick intact bone cortex is not possible at low pulse energy. **d**. 3D rendered images (400x400x300 µm³, 518x518x150 voxel) of SHG (gray), THG (blue), GFP (green) and tdTomato (red) fluorescence in the intact tibia of a Cdh5:tdTomato x Histone:GFP mouse (12 weeks old), acquired at 1330 nm, 2 MHz (tunable OPA) or at 1330 nm, 80 MHz (OPO) in **e**. Both excitation sources (OPA and OPO) were adjusted at 30 mW, corresponding to pulse energies of 15 nJ for the OPA and 0.38 nJ for the OPO, (without adaptation in z-direction) and the pixel dwell time was kept the same, namely 2.46 µs (2x frame acquisition). The orange arrows in both d and e indicate the direction of illumination (the optical axis of the microscope). Imaging of SHG, THG, tdTomato and GFP fluorescence signals in the tibia (cortex and marrow) was achieved with the high, but not with the low pulse energy. This indicates that high pulse energy is key for achieving large imaging depth by three-photon excitation. Scale bar = 100 µm for all images.

Supplemental Figure 5 related to Figure 5

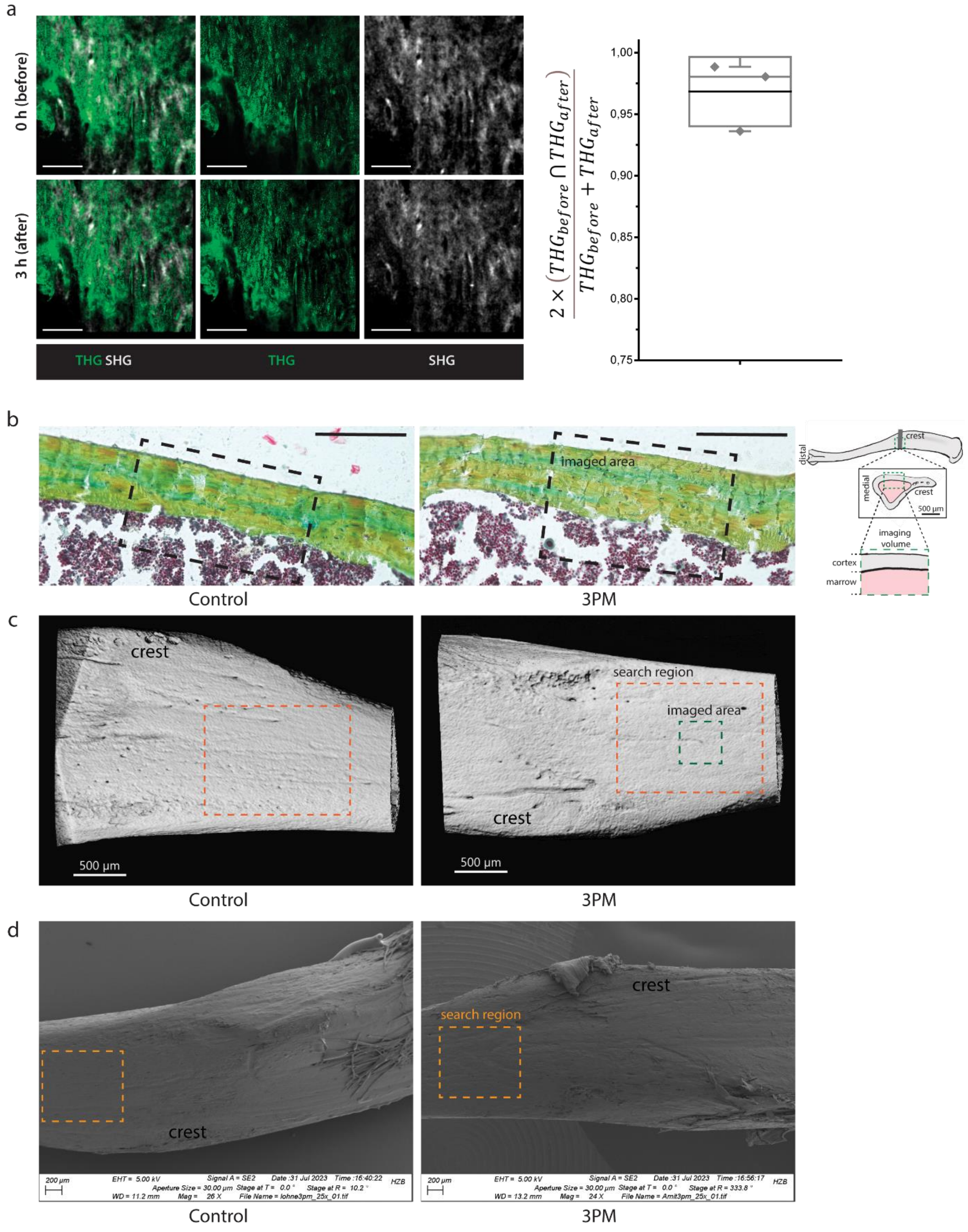

Analysis of the bone surface integrity after time-lapse intravital tibia imaging upon excitation with high pulse energy at 1650 nm. a. Representative maximum intensity projections (30 µm thick) of THG and SHG signal at the tibia surface before and after intravital imaging at 1650 nm (3 MHz, 80 mW). The intravital imaging was

performed in a time-lapse manner over 3 hours. Scale bar = 100 µm. The difference between the THG signal before and after intravital time-lapse imaging over 3 hours calculated as $2\left(THG_{before} \cap THG_{after}\right)/\left(THG_{before} + THG_{after}\right)$ is displayed for 3 CD19:tdRFP mice. **b**. Representative Movat's pentachrom staining of the bone cortex of a murine tibia exposed to 1650 nm, 3 MHz (80 mW) over 3 hours, during intravital time-lapse imaging. A non-exposed tibia was used as control. Scale bar = 200 µm. **c**. Representative NanoCT 3D reconstructions of a tibia exposed for 2 hours to 1650 nm laser radiation (3 MHz, 110 mW, 35.6 nJ) during intravital imaging in comparison to a non-exposed tibia. Search region (orange dashed rectangle) is region, from which the area to be imaged was chosen. Dark green dashed square is representative for the imaged area. Scale bar = 500 µm. **d**. Representative SEM images of the surface of a tibia exposed for 2 hours to 1650 nm laser radiation (3 MHz, 115 mW, 37.2 nJ) during intravital imaging (3PM) in comparison to a non-exposed tibia (Control). Similar to **c**, the orange dashed rectangle represents the search region. Scale bar = 200 µm.

Supplemental Figure 6 related to Figure 5

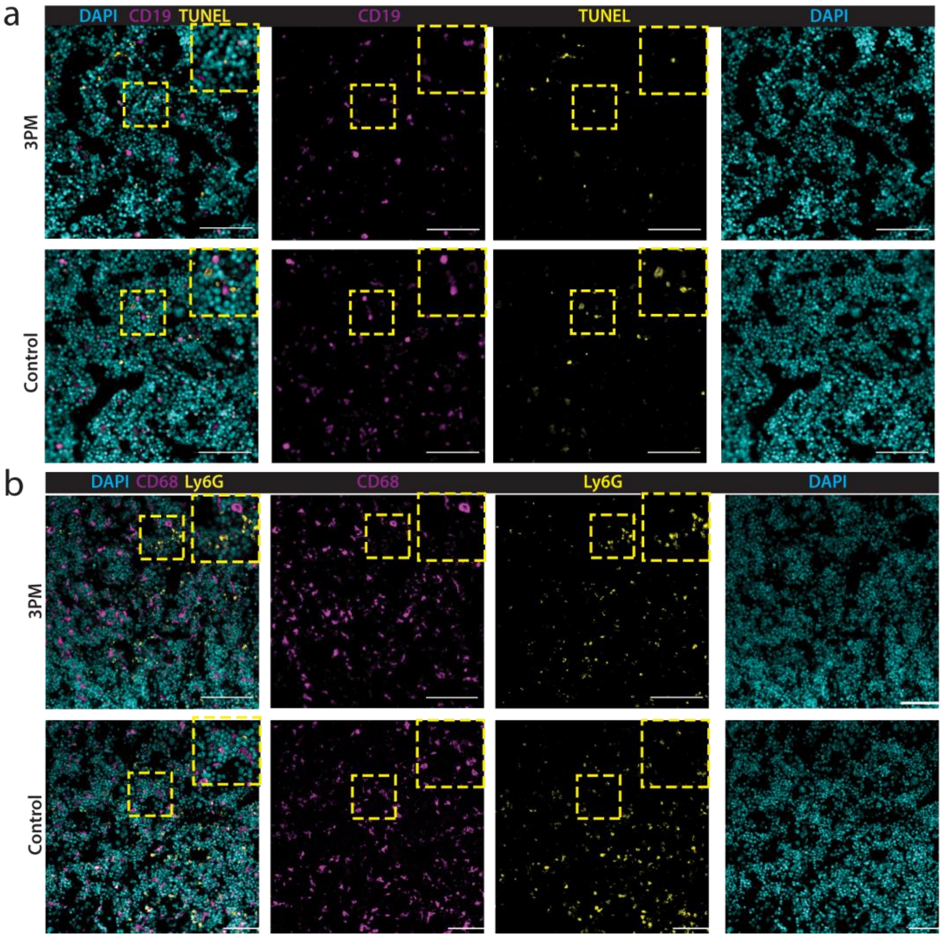

Immunofluorescence analysis of tissue photodamage after time-lapse imaging upon excitation with high pulse energy at 1650 nm (120 mW at the bone surface) in tibia marrow a. Representative immunofluorescence overlays of TUNEL (apoptosis, yellow), nuclear (DAPI, cyan) and CD19 (B lineage cells, magenta) and **b**. overlays of macrophages (CD68, magenta), neutrophil granulocytes (Ly6G, yellow), and nuclear (DAPI, cyan) staining, in tibia marrow tissue irradiated at high pulse energy 1650 nm (3.09 MHz, 120 mW), and not irradiated (control). Scale bar = 200 µm.

Supplemental Figure 7 related to Figure 5

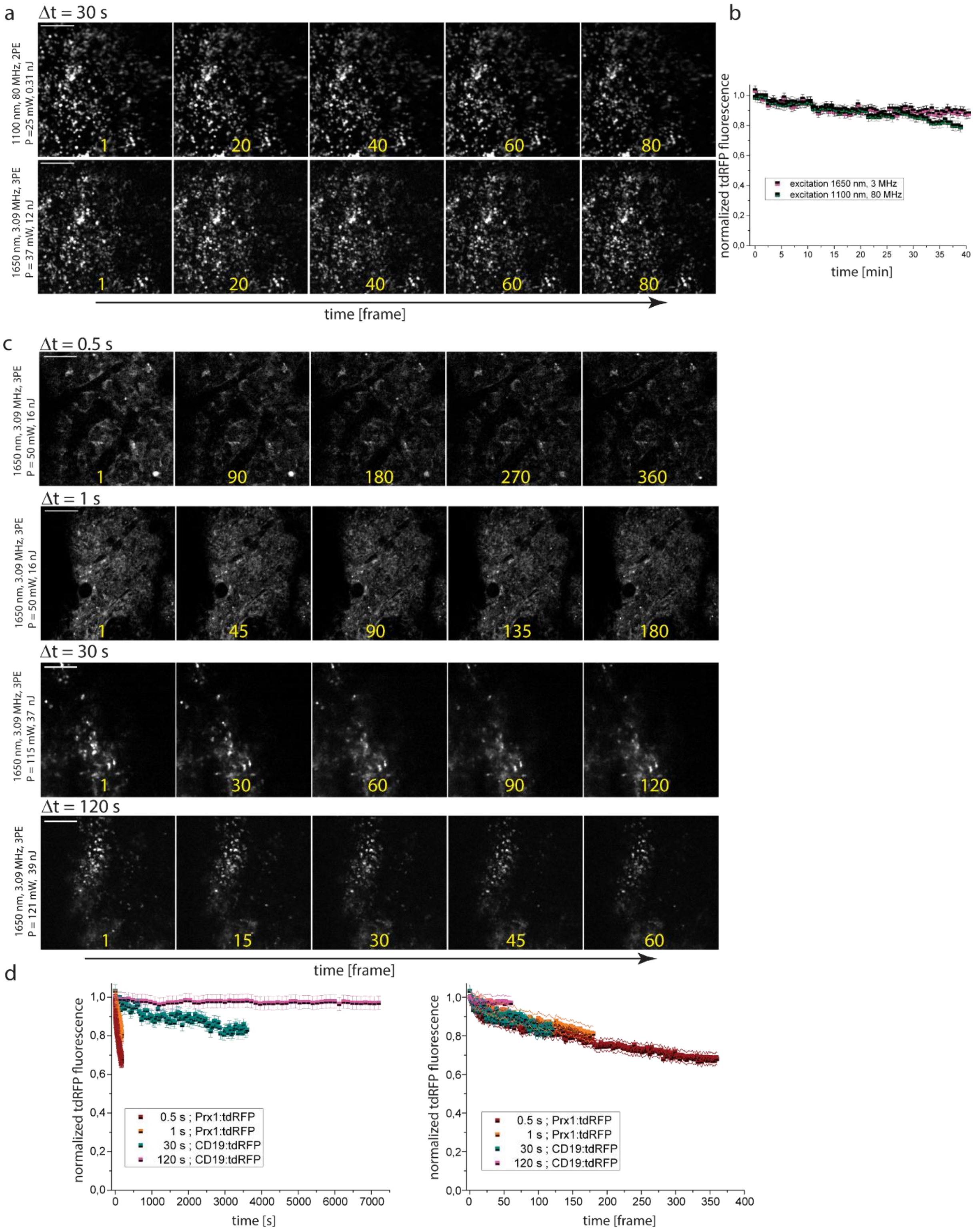

Photobleaching analysis during intravital time-lapse tibia imaging upon excitation with high-pulse energy 1650 nm radiation. a. Intravital time-series of selected tdRFP fluorescence images in the tibia of a CD19:tdRFP mouse upon excitation at 1100 nm, 80 MHz (2PM, low pulse energy) and at 1650 nm, 3.09 MHz

(3PM, high pulse energy). Time-lapse imaging was performed every 30 s, over 60 minutes in 2PM regime, followed by 60 minutes in 3PM regime. Scale bar 100 µm. **b**. Graphs represent the decay of the average tdRFP fluorescence signal during intravital imaging every 30 s, over 40 min, upon excitation at 1100 nm, 80 MHz and at 1650 nm, 3 MHz, respectively, at the indicated average powers and pulse energies. Fluorophore (tdRFP) photobleaching, expected to scale highly non-linearly with the laser power[6,11], may limit the suitability of 1650 nm excitation for time-lapse *in vivo* applications. Our measurements show slightly lower photobleaching rates at 1650 nm as compared to 1100 nm, i.e. $k_{photobl} = 1.3\cdot10^{-3}$ min^{-1} at 1650 nm and $k_{photobl} = 2.4\cdot10^{-3}$ min^{-1} at 1100 nm. **c**. Intravital time-series of selected tdRFP fluorescence images in the tibia of Prx1:tdRFP (time step 0.5 s and 1 s) or CD19:tdRFP (time step 30 s and 120 s) mice upon excitation at 1650 nm, 3 MHz (high pulse energy). Scale bar 100 µm. **d**. Graphs represent the decay of the average tdRFP fluorescence signal in the two indicated mouse strains during intravital tibia imaging for various time steps, i.e. 0.5 s, 1 s, 30 s, and 120 s, respectively. In the left graph, the time is given in minutes, whereas in the right graph, the time is given in number of acquired frames. We determined by single-exponential fitting $k_{photobl} = 9.85\cdot10^{-2}$ min^{-1} for a time step of 0.5 s, $k_{photobl} = 6.92\cdot10^{-2}$ min^{-1} for 1 s, $k_{photobl} = 1.3\cdot10^{-3}$ min^{-1} for 30 s, and $k_{photobl} = 0.56\cdot10^{-3}$ min^{-1} for 120 s. However, if considering the number of acquired images (frame number), the photobleaching rates are similar, independent of the acquisition rate: $k_{photobl} = 9.13\cdot10^{-4}$ frame^{-1} (0.5 s), $k_{photobl} = 10.9\cdot10^{-4}$ frame^{-1} (1 s), $k_{photobl} = 13.3\cdot10^{-4}$ frame^{-1} (30 s) and $2.43\cdot10^{-4}$ frame^{-1} (120 s). Thus, the photobleaching depends on the number of acquired frames, rather than on the time step between consecutive acquisitions.

Supplemental Figure 8 related to Figure 6

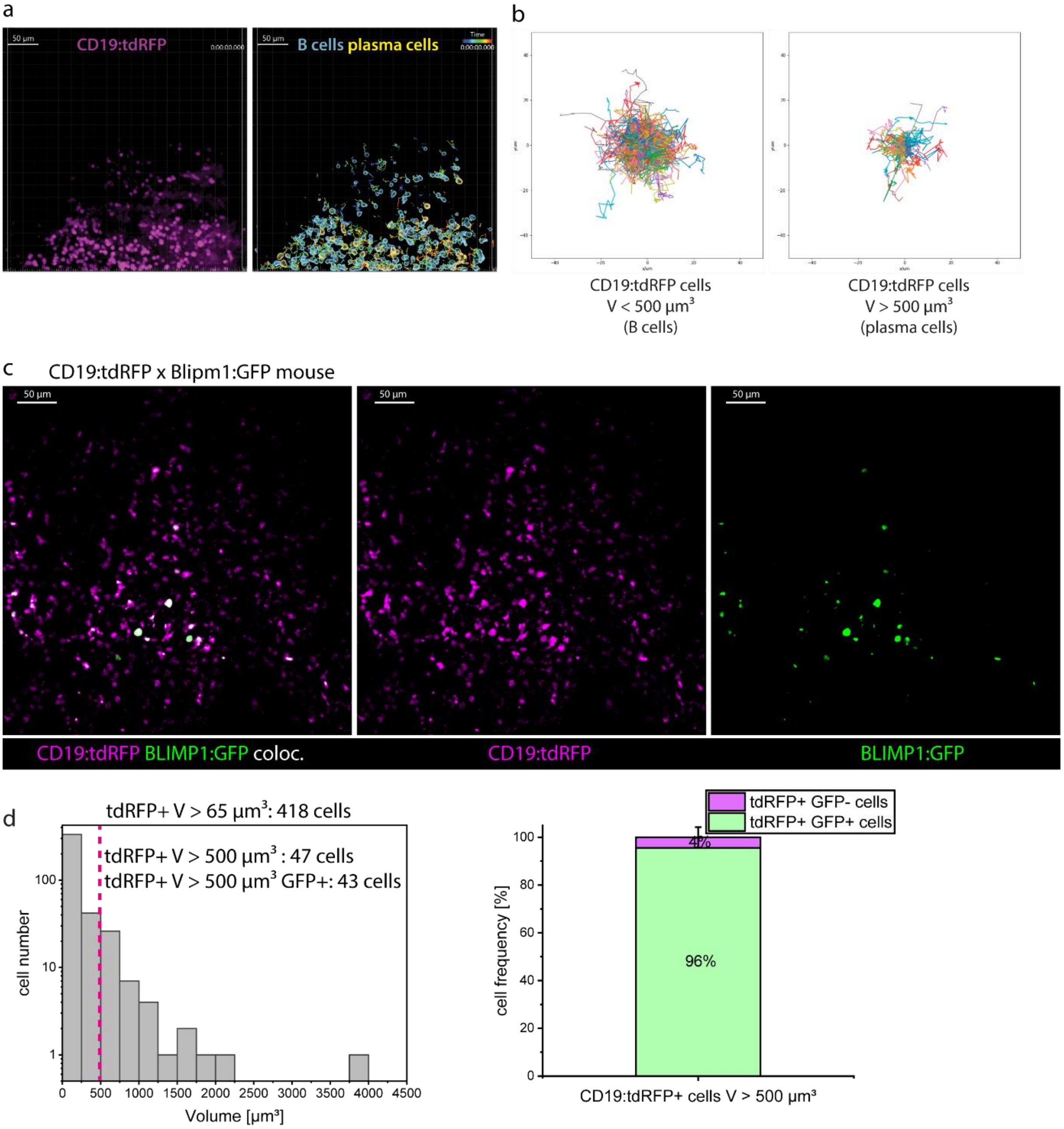

Three-photon imaging of bone marrow B lineage cells in the tibia of CD19:tdRFP and CD19:tdRFP x Blimp1:GFP mice. a. Time-lapse intravital imaging in the intact tibia of a CD19:tdRFP mouse upon excitation at 1650 nm, 4 MHz, over a time window of 2 hours, with a time step of 120 s. Representative 3D reconstructions of tdRFP fluorescence signal (magenta) of B lineage cells (400x400x60 µm^3, 518x518x11 voxels) and segmented cells, differentiating between B cells (volume < 500 µm^3, cyan outlines) and plasma cells (volume > 500 µm^3, yellow outlines). Scale bar = 50 µm. **b**. Corresponding rose plots displaying the trajectories of B cells and plasma cells, respectively. **c.** Representative single channel and merged fluorescence images of B

lymphocytes in the tibia marrow of a CD19:tdRFP x Blimp1:GFP mouse, showing colocalization of GFP and tdRFP fluorescence in cells with a volume larger than 500 μm^3. Scale bar = 100 µm. **d.** Left graph shows the volume distribution of marrow CD19: tdRFP+ cells shown in **c**. In this example, we found 45 tdRFP+ cells having a volume above 500 μm^3, among which 40 cells are GFP+, i.e. Blimp1+ plasma cells. Right graph shows the analysis of 11 murine tibia CD19:tdRFP x Blimp1:GFP marrow regions, showing that 96% of tdRFP+ cells with a volume larger than 500 μm^3 are GFP positive.

Supplemental Figure 9 related to Figure 7

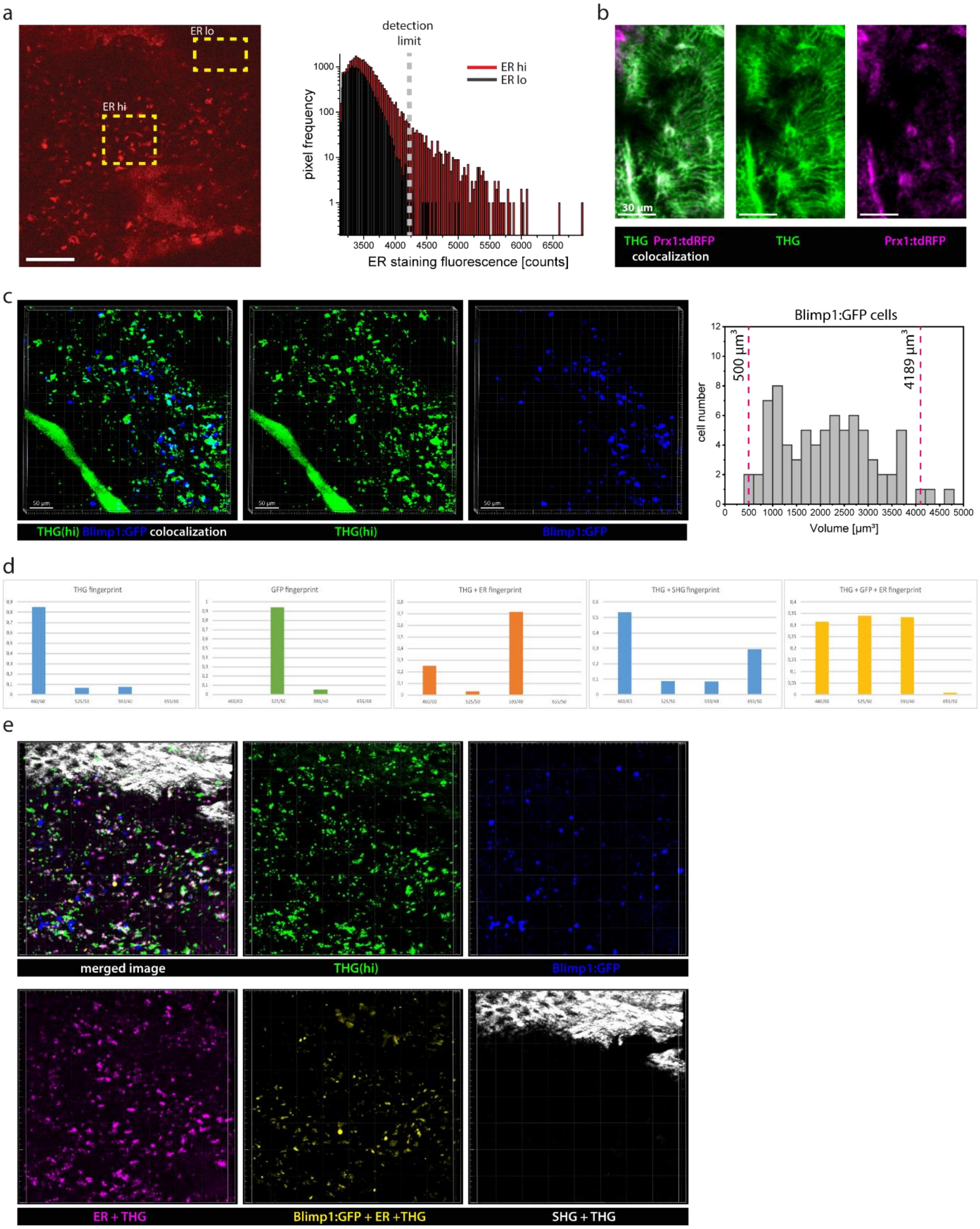

Analysis of third harmonics generation signal and endoplasmic reticulum (ER) staining in the tibia cortex and marrow. a. Representative fluorescence image of ER staining in the bone marrow of a murine tibia. Scale bar = 100 µm. The yellow rectangles indicate a background region and a region with cells positively stained for ER. Histogram distributions of background and ER staining fluorescence signal,

respectively (graph in the right panel). The upper limit of the background is defined as the limit for positive (abundant) ER staining, i.e. ER detection limit. **b.** Image of lacunae and canaliculi (THG signal, green) and osteocytes hosted therein ($Prx1^+$ tdRFP fluorescent cells, magenta) in the bone cortex of tibia of a Prx1:tdRFP mouse, acquired upon three-photon excitation at 1650 nm, 3 MHz. Scale bar = 30 µm. Graph shows the cell volume distribution of Blimp1:GFP cells in the tibia marrow, demonstrating that marrow plasma cells have a cell volume of minimum 500 μm^3, not exceeding 4200 μm^3. **c**. 3D image (442x442x100 μm^3, 1010x1010x51 voxel) of THG (green) and GFP (blue) in the bone marrow of an explanted Blimp1:GFP mouse tibia (3PM at 1330 nm). Scale bar = 50 µm. **d**. *In situ* finger prints for SIMI spectral unmixing in the tibia marrow of Blimp1:GFP mice, detected upon excitation at 1330 nm in the channels 460/60, 525/50, 593/40 and 655/50. We could identify pixels displaying only THG signal, THG and SHG signals, only GFP signal, THG and ER tracker signals and GFP, ER tracker and THG signals. **e**. Merged 3D image (400x400x60 μm^3, 518x518x31 voxel, upper left) and spectrally (using SIMI) unmixed tissue components in an explanted Blimp1:GFP mouse tibia (3PM at 1330 nm). Scale bar = 50 µm.

Supplemental Figure 10 related to Figure 7

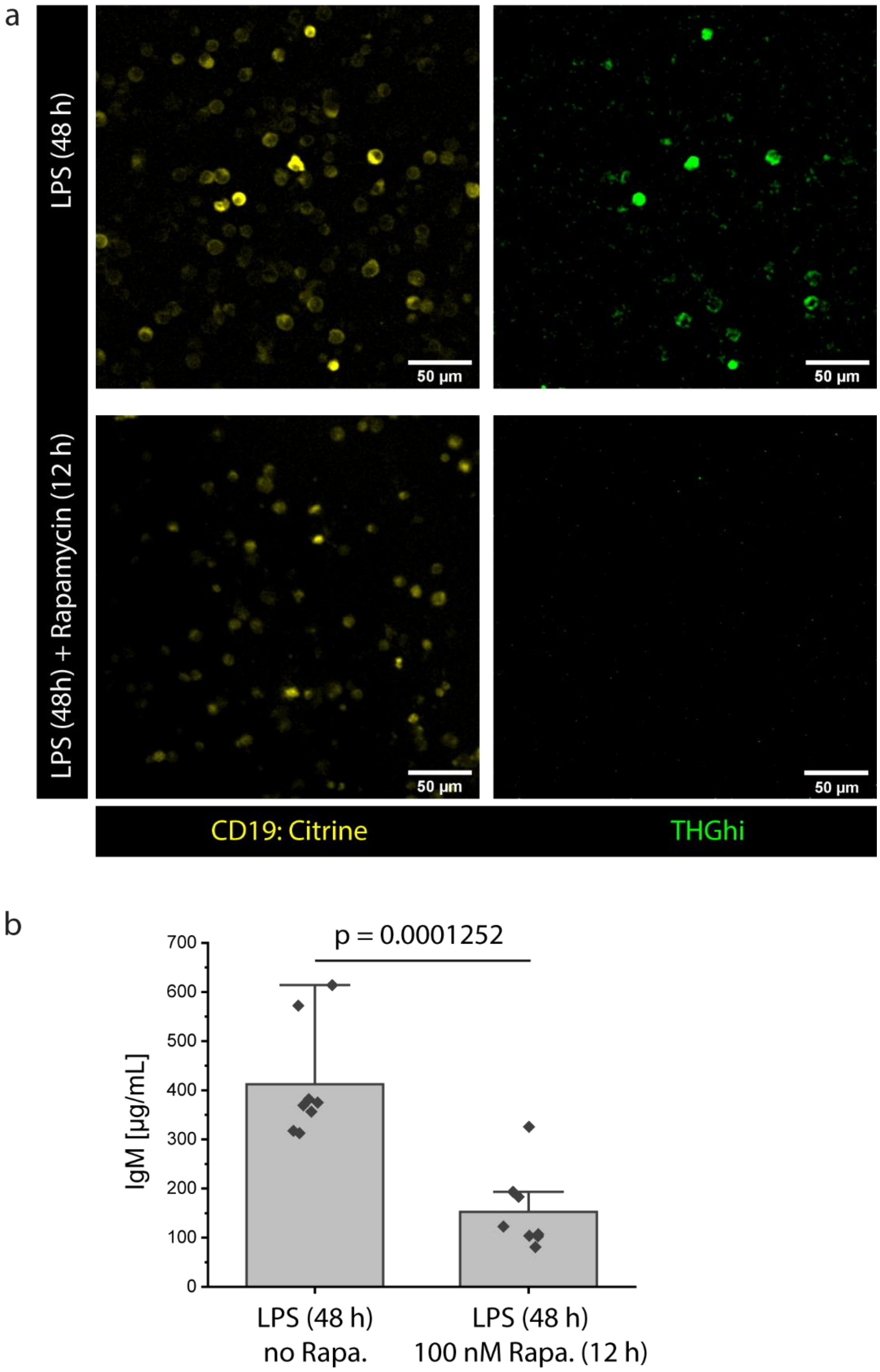

Third harmonics generation (THG^{hi}) signal in LPS blasts in response to rapamycin treatment. a. Representative Citrine fluorescence and THG^{hi} signal images of marrow CD19 fate mapping cells (B cells) upon treatment with lipopolysaccharide (LPS) for 48 hours, with and without 12 hours exposure to rapamycin (100 nmol/L). Citrine yellow fluorescence (left column, detection at 525±25

nm) shows the activated B cells (LPS blasts), which have similar antibody secreting properties as plasma cells, THG^{hi} signal is shown in green (right column, detection at 447±20 nm). Excitation was performed at 1300 nm. Scale bar = 50 µm. **b.** IgM secretion (in µg/mL) of CD19:Citrine+ cells upon LPS stimulation over 48 h, with and without rapamycin (100 nmol/L) treatment over 12 h, measured by Enzyme-Linked Immunosorbent Assay (ELISA) in cell supernatant. Upon treatment with rapamycin antibody producing LPS blasts reduce their antibody secretion, as previously shown[13]. Statistical test: one-way ANOVA, p-value indicated in graph.

Supplemental Tables

Suppl. Table I related to Fig. 1: Main parameters characterizing the microscope system: Maximum average power, maximum pulse energy, lateral (xy) and axial (z) dimensions of the point-spread function (PSF), i.e. dimensions of 100 nm fluorescent nanospheres embedded in agarose, measured using the Olympus lens XLPLN25XWMP2, NA 1.05.

laser/wavelength		rep. rate (MHz)	max. av. P/mW	max. pulse energy /nJ	pulse width / fs -laser output	pulse width / fs -under the lens	PSF xy / nm	PSF z / nm	Δλ / nm
Ti:Sa	930 nm	80	720	9.0	140	247	368±27	1322±89	7
OPO	1100 nm	80	230	2.875	200	166	407±42	1503±158	12
	1330 nm	80	66	0.825	200	166	-	-	16
tunable OPA	1330 nm	2	145	72.5	50	55	533±48	1537±167	-
	1630 nm	2	75	37.5	60	75	635±39	1700±175	-
Ytterbia OPA	1650 nm	1.01	144	142.6	65	72	587±109	1893±210	60
	1650 nm	2.06	286	138.8	65	71	691±31	1806±152	60
	1650 nm	3.09	376	130.7	65	73	684±72	1877±174	60
	1650 nm	3.98	568	142.7	65	75	669±55	1882±160	60

Suppl. Table II related to Fig. 4: Parameter overview for *in vivo* time-lapse 2D imaging experiments in the tibia of Prx1:tdRFP mice (n = 2).

exc. scheme	time step [s]	acq. time [min]	cortex thick-ness [µm]	l_e [µm]	marrow thick-ness [µm]	l_e [µm]	pulse energy at surface [nJ]	eff. pulse energy [nJ]	power at surface [mW]	pixel dwell time [µs]	
1650 nm, 3 MHz	1	3	96	89	30	259	16	4.9	50	1.98	Fig. 4; Video 3
1650 nm, 4 MHz	1	3	80	100	40	291	14	5.5	57	1.98	Fig. 4; Video 4

Suppl. Table III related to Fig. 5, 6 and 7: Parameter overview for *in vivo* time-lapse 3D imaging experiments in the tibia of CD19:tdRFP mice (n = 6).

excitation scheme	time step [s]	acq. time [min]	cortex thick-ness [µm]	l_e [µm]	marrow thick-ness [µm]	l_e [µm]	pulse energy at surface [nJ]	eff. pulse energy [nJ]	mean power at surface [mW]	B lineage cells displ. rate [µm/min]	
1100 nm, 80 MHz	30	60	60	76	50	165	0.23	0.17	18	0.57±0.20	
1650 nm, 3 MHz	30	30	60	106	50	333	8	3.9	25	0.54±0.23	
1100 nm, 80 MHz	30	30	80	58	70	91	0.38	0.07	30	0.49±0.20	Fig. 5; Video 6
1650 nm, 3 MHz	30	60	80	120	70	345	13	5.4	42	0.53±0.19	Fig. 5; Fig. 7; Video 6
1100 nm, 80 MHz	30	40	70	60	60	156	0.31	0.07	25	0.64±0.15	Suppl. Fig. 7
1650 nm, 3 MHz	30	60	70	107	60	183	12	4.5	37	0.61±0.31	Suppl. Fig. 7
1650 nm, 3 MHz	30	30	170	129	120	233	37	5.9	115	0.87±0.37	
1650 nm, 3 MHz	120	120	170	129	120	233	37	5.9	115	0.30±0.16	Suppl. Fig. 7
1650 nm, 3 MHz	30	60	180	118	110	182	39	4.6	121	0.72±0.65	Fig. 6; Suppl. Fig. 7;Video 7
1650 nm, 3 MHz	120	120	180	118	110	182	39	4.6	121	0.40±0.15	Fig. 6; Video 7
1650 nm, 4 MHz	120	120	150	112	70	238	22	4.3	87	0.45±0.20	Suppl. Fig. 8; Video 8, 9

Supplemental References

1. Horton, N. G. & Xu, C. Dispersion compensation in three-photon fluorescence microscopy at 1,700 nm. *Biomed Opt Express* **6**, 1392-1397 (2015). https://doi.org:10.1364/BOE.6.001392
2. Richards, B. W., E. Electromagnetic diffraction in optical systems, II. Structure of the image field in an aplanatic system. *Proc. Royal Soc. A* **253** (1959). https://doi.org:https://doi.org/10.1098/rspa.1959.0200
3. Gu, M. *Advanced Optical Imaging Theory*. (Springer, 2000).
4. Hontani, Y., Xia, F. & Xu, C. Multicolor three-photon fluorescence imaging with single-wavelength excitation deep in mouse brain. *Sci Adv* **7** (2021). https://doi.org:10.1126/sciadv.abf3531
5. Loudon, R. *The quantum theory of light, 2nd edition* (Claredon, 1983).
6. Herz, J. *et al.* Expanding two-photon intravital microscopy to the infrared by means of optical parametric oscillator. *Biophys J* **98**, 715-723 (2010). https://doi.org:10.1016/j.bpj.2009.10.035
7. Drobizhev, M., Tillo, S., Makarov, N. S., Hughes, T. E. & Rebane, A. Absolute two-photon absorption spectra and two-photon brightness of orange and red fluorescent proteins. *J Phys Chem B* **113**, 855-859 (2009). https://doi.org:10.1021/jp8087379
8. Shim, J., Iwaya, C., Ambrose, C. G., Suzuki, A. & Iwata, J. Micro-computed tomography assessment of bone structure in aging mice. *Sci Rep* **12**, 8117 (2022). https://doi.org:10.1038/s41598-022-11965-4
9. Choe, K. *et al.* Intravital three-photon microscopy allows visualization over the entire depth of mouse lymph nodes. *Nat Immunol* (2022). https://doi.org:10.1038/s41590-021-01101-1
10. Charan, K., Li, B., Wang, M., Lin, C. P. & Xu, C. Fiber-based tunable repetition rate source for deep tissue two-photon fluorescence microscopy. *Biomed Opt Express* **9**, 2304-2311 (2018). https://doi.org:10.1364/BOE.9.002304
11. Hopt, A. & Neher, E. Highly nonlinear photodamage in two-photon fluorescence microscopy. *Biophys J* **80**, 2029-2036 (2001). https://doi.org:10.1016/S0006-3495(01)76173-5
12. Logan, M. *et al.* Expression of Cre Recombinase in the developing mouse limb bud driven by a Prxl enhancer. *Genesis* **33**, 77-80 (2002). https://doi.org:10.1002/gene.10092
13. Jones, R. B. & Walker, B. D. HIV-specific CD8(+) T cells and HIV eradication. *J Clin Invest* **126**, 455-463 (2016). https://doi.org:10.1172/JCI80566
