# Supplementary material for: Optimized intravital three-photon imaging of intact mouse tibia links plasma cell motility to functional states
Source: iScience. 2024 Sep 17;27(10):110985. doi: 10.1016/j.isci.2024.110985 (PMC11466647; doi:10.1016/j.isci.2024.110985)
Supplement: Data S1. Theoretical background of three- and two-photon-excitation [file mmc2.pdf]

Supplemental Information

1.1. Theoretical background of fluorescence signal upon non-resonant or resonance-enhanced two- and three-photon excitation

We consider that a fluorophore molecule transits only virtual energetic states during a non-resonant excitation, either two- or three-photon excitation. Only the initial and final energetic states of the fluorophore molecule are real energetic states. During a resonance-enhanced three-photon excitation, the molecule also transits real energetic states, not only virtual states, which increases the excitation probability and, by that, leads to an increased excitation rate as compared to a non-resonant three-photon excitation process.

The experimentally measured fluorescence signal is given by the fluorescence signal per laser pulse *F,* integrated over the pixel dwell time, i.e. the number of excitation pulses within the pixel dwell time.

The fluorescence photon flux density per laser pulse *F*, originating from a sample containing molecules of a single fluorophore type, is given by:

$$F_{2P} \;= \frac{1}{2} {N_{abs}}^{2P} N_{abs}^{2P} \;\int_0^{\infty} \varphi(\lambda_{em}) \cdot \zeta(\lambda_{em}) d\lambda_{em} \tag{1}$$

for a non-resonant two-photon excitation process and

$$F_{3P} \;= \frac{1}{3} {N_{abs}}^{3P} \int_0^{\infty} \varphi(\lambda_{em}) \cdot \zeta(\lambda_{em}) d\lambda_{em} \tag{2}$$

for a non-resonant three-photon excitation process. In Eq. (1) and (2) N_{abs} represents the number of absorbed photons, φ the fluorescence quantum yield and ζ the detector quantum efficiency of the experimental setup.

As fluorescence quantum yield – the spontaneous emission of photons by an excited molecule – is considered to be independent of the excitation process and as fluorescence detection in all our experiments does not change, we simplify the integrals in Eq. (1) and Eq. (2) by $\bar{\varphi}$.

$$\bar{\varphi} = \int_0^{\infty} \varphi(\lambda_{em}) \cdot \zeta(\lambda_{em}) d\lambda_{em} \tag{3}$$

The number of absorbed photons per molecule N_{abs} depends on the excitation rate, i.e. either non-resonant two- or three-photon excitation. The excitation rate depends on the capacity of the molecule to simultaneously be excited by two and three photons,

respectively. The measure of this capacity is given by the active two-photon absorption cross-section $\sigma_{2P} \cdot \eta_{2P}$ for a non-resonant two-photon excitation process and the active three-photon absorption cross-section $\sigma_{2P} \cdot \eta_{3P}$ for a non-resonant three-photon excitation process. Further, the excitation rate depends on the excitation photon flux density of the laser under the objective lens, i.e. time-dependent intensity of the excitation source. N_{abs} is also dependent on the number of molecules within the excitation volume, given by the concentration *C*. Thus, according to, the number of absorbed photons is for a two-photon excitation:

$$N_{abs}{}^{2P} = C \cdot \sigma_{2P} \cdot \eta_{2P} \cdot \int_t \int_{V\to\infty} S^2(\boldsymbol{r}) \cdot I_0{}^2(t)dtdV \quad (4)$$

and for a three-photon excitation:

$$N_{abs}{}^{3P} = C \cdot \sigma_{3P} \cdot \eta_{3P} \cdot \int_t \int_{V\to\infty} S^3(\boldsymbol{r}) \cdot I_0{}^3(t)dtdV \quad (5)$$

with $I_0(t)$ the time dependent laser photon flux and $S(\boldsymbol{r})$ its unitless three-dimensional spatial distribution function.

As temporal and spatial distributions of the excitation photon flux can be considered to be independent from each other, Eq. (4) turns into:

$$N_{abs}{}^{2P} = C \cdot \sigma_{2P} \cdot \eta_{2P} \cdot \int_t I_0{}^2(t)dt \int_{V\to\infty} S^2(r) \cdot dV \quad (6).$$

If $g^{(2)}$ is the degree of second-order coherence of the excitation source being defined as $\frac{\langle I_0{}^2(t)\rangle}{\langle I_0(t)\rangle^2}$ [5], Eq. (6) becomes:

$$N_{abs}{}^{2P} = C \cdot \sigma_{2P} \cdot \eta_{2P} \cdot g^{(2)}\langle I_0\rangle^2 \int_{V\to\infty} S^2(r) \cdot dV \quad (7),$$

with ⟨ ⟩ indicating the average over time.

Similarly, Eq. (5) turns into:

$$N_{abs}{}^{3P} = C \cdot \sigma_{3P} \cdot \eta_{3P} \cdot \int_t I_0{}^3(t)dt \int_{V\to\infty} S^3(r) \cdot dV \quad (8).$$

If $g^{(3)}$ is now the degree of third-order coherence of the excitation source being defined as $\frac{\langle I_0{}^3(t)\rangle}{\langle I_0(t)\rangle^3}$, Eq. (8) becomes:

$$N_{abs}{}^{3P} = C \cdot \sigma_{3P} \cdot \eta_{3P} \cdot g^{(3)}\langle I_0\rangle^3 \int_{V\to\infty} S^3(r) \cdot dV \quad (9).$$

For solving the temporal integrals $\int_t I_0{}^2(t)dt$ and $\int_t I_0{}^3(t)dt$, we assume, based on our experimental data, Gaussian laser pulses for both optical parametric oscillator as a

two-photon excitation source (OPO) and both optical parametric amplifiers as two and three-photon excitation sources (OPA). According to, we define the origin of the time axis at the maximum of the laser pulse, with the pulse width ${\tau_p}^{2P}$ and ${\tau_p}^{3P}$ as well as the repetition rates RR_{2P} and RR_{3P} for the OPO and OPA lasers, respectively. Hence, the temporal integral in Eq(6) for a two-photon excitation becomes:

$$\int_{-1/(2\cdot RR_{2P})}^{1/(2\cdot RR_{2P})} {I_0}^2(t)dt = \frac{{g_p}^{2P}}{{\tau_p}^{2P}} \cdot \left[\int_{-1/(2\cdot RR_{2P})}^{1/(2\cdot RR_{2P})} I_0(t)dt\right]^2 \quad (10)$$

with ${g_p}^{2P}$=0.664 for a Gaussian beam profile of a single-mode laser pulse (TEM_{00}). Thus, the degree of second-order coherence of the excitation source is $g^{(2)} = \frac{{g_P}^{2P}}{RR_{2P}\cdot {\tau_p}^{2P}}$.

Similarly, the temporal integral in Eq. (8) for a three-photon excitation becomes:

$$\int_{-1/(2\cdot RR_{3P})}^{1/(2\cdot RR_{3P})} {I_0}^3(t)dt = \frac{{g_p}^{3P}}{\left({\tau_p}^{3P}\right)^2} \cdot \left[\int_{-1/(2\cdot RR_{3P})}^{1/(2\cdot RR_{3P})} I_0(t)dt\right]^3 \quad (11)$$

with ${g_p}^{3P}$=0.509 for a Gaussian laser pulse. Thus, the degree of third-order coherence of the excitation source is $g^{(3)} = \frac{{g_P}^{3P}}{{RR_{3P}}^2\cdot \left({\tau_p}^{3P}\right)^2}$.

Assuming the laser beam is diffraction limited, with *NA* the numerical aperture, i.e. *NA* = $n\cdot\sin(\alpha)$ with *n* being the refractive index of the medium and 2α the maximal angle of the light cone of the objective lens), the relation between the time-dependent average intensity $\langle I_0\rangle$ at the focal plane and average laser power $\langle P\rangle$ under the objective lens is defined as:

$$\langle I_0\rangle = \frac{\pi\cdot NA^2}{{\lambda_{exc}}^2} \cdot \langle P\rangle \quad (12),$$

with λ_{exc} the excitation wavelength.

From Eq. (7), (10) and (12) for a two-photon excitation process, it follows:

$${N_{abs}}^{2P} = C \cdot \sigma_{2P} \cdot \eta_{2P} \cdot \frac{{g_P}^{2P}}{RR_{2P}\cdot {\tau_p}^{2P}} \frac{\pi^2\cdot NA^4}{{\lambda_{exc}}^4} \cdot \langle P\rangle^2 \int_{V\to\infty} S^2(r) \cdot dV \quad (13).$$

Similarly, from Eq. (9), (11) and (12) for a three-photon excitation process, it follows:

$${N_{abs}}^{3P} = C \cdot \sigma_{3P} \cdot \eta_{3P} \cdot \frac{{g_P}^{3P}}{{RR_{3P}}^2\cdot \left({\tau_p}^{3P}\right)^2} \frac{\pi^3\cdot NA^6}{{\lambda_{exc}}^6} \cdot \langle P\rangle^3 \int_{V\to\infty} S^3(r) \cdot dV \quad (14).$$

In the following we use the previously defined[3] unitless coordinate ν in planes perpendicular to the optical axis, defined as $\nu = \frac{2\pi \cdot NA \cdot \rho}{\lambda_{exc}}$, and the unitless coordinate *u* along the optical axis of the microscope, defined as $u = \frac{2\pi \cdot NA^2 \cdot z}{n \cdot \lambda_{exc}}$, with ρ and z the coordinates in the metric unit system. The spatial distribution of the excitation photon flux under the objective lens is defined as:

$$S(\boldsymbol{r}) = h^2(u, \nu) \qquad (15)$$

with *h(u, ν)* the point-spread function (PSF), which we consider to be Gaussian for both u and ν coordinates based on our experimental observations. Consequently, the spatial integrals $\int_{V\to\infty} S^2(r) \cdot dV$ in Eq. (13) and $\int_{V\to\infty} S^3(r) \cdot dV$ in Eq(14) are estimated to be:

$$\int_{V\to\infty} S^2(r) \cdot dV = \int\int h^4(u,\nu) \cdot dud\nu \approx \frac{n \cdot {\lambda_{exc}}^3}{\pi \cdot NA^4} \qquad (16)$$

for two-photon excitation, in accordance with, and

$$\int_{V\to\infty} S^3(r) \cdot dV = \int\int h^6(u,\nu) \cdot dud\nu \approx \frac{2\sqrt{2}}{3\sqrt{3}} \cdot \frac{n \cdot {\lambda_{exc}}^3}{\pi \cdot NA^4} = 0.544 \cdot \frac{n \cdot {\lambda_{exc}}^3}{\pi \cdot NA^4} \qquad (17)$$

for three-photon excitation.

Replacing the integral in Eq. (13) with the result of Eq. (16) and that in Eq. (14) with the result from Eq. (17), we obtain the number of absorbed photons per laser pulse for a two- and three-photon excitation process, respectively:

$$N_{abs}{}^{2P} = C \cdot \sigma_{2P} \cdot \eta_{2P} \cdot \frac{{g_P}^{2P}}{RR_{2P} \cdot {\tau_p}^{2P}} \frac{\pi \cdot n}{\lambda_{exc}} \cdot \langle P \rangle^2 \qquad (18)$$

$$N_{abs}{}^{3P} = C \cdot \sigma_{3P} \cdot \eta_{3P} \cdot \frac{{g_P}^{3P}}{{RR_{3P}}^2 \cdot \left({\tau_p}^{3P}\right)^2} \cdot 0.544 \cdot \frac{\pi^2 \cdot n \cdot NA^2}{{\lambda_{exc}}^3} \cdot \langle P \rangle^3 \qquad (19)$$

Hence, the fluorescence signal per laser pulse for the two excitation schemes is given by:

$$F_{2P}(t) = \frac{1}{2} \bar{\varphi} \cdot \sigma_{2P} \cdot \eta_{2P} \cdot C \cdot \frac{{g_P}^{2P}}{RR_{2P} \cdot {\tau_p}^{2P}} \frac{\pi \cdot n}{\lambda_{exc}} \cdot \langle P \rangle^2 \qquad (20)$$

and

$$F_{3P}(t) = \frac{1}{3} \bar{\varphi} \cdot \sigma_{3P} \cdot \eta_{3P} \cdot C \cdot \frac{{g_P}^{3P}}{{RR_{3P}}^2 \cdot \left({\tau_p}^{3P}\right)^2} \cdot 0.544 \cdot \frac{\pi^2 \cdot n \cdot NA^2}{{\lambda_{exc}}^3} \cdot \langle P \rangle^3 \qquad (21).$$

In order to estimate the active three-photon excitation cross-section from the known value of the active two-photon excitation cross-section for the same fluorophore, at the same site of the sample, we use the ratio between the fluorescence signal per laser pulse upon two- and three-photon excitation:

$$\frac{F_{2P}}{F_{3P}} = \frac{\frac{1}{2}\cdot\sigma_{2P}\cdot\eta_{2P}\cdot\frac{0.664}{RR_{2P}\cdot\tau_p{}^{2P}}\frac{1}{\lambda_{exc}{}^{2P}}\cdot\langle P^{2P}\rangle^2}{\frac{1}{3}\cdot\sigma_{3P}\cdot\eta_{3P}\cdot\frac{0.509}{RR_{3P}{}^2\cdot(\tau_p{}^{3P})^2}\cdot 0.544\cdot\frac{\pi\cdot NA^2}{(\lambda_{exc}{}^{3P})^3}\cdot\langle P^{3P}\rangle^3} \quad (22).$$

By substitution, the active three-photon excitation cross-section $\eta_{3P}\sigma_{3P}$ with the unit $cm^6\cdot s^2$ is given by:

$$\sigma_{3P}\cdot\eta_{3P} = \frac{3}{2}\cdot\frac{F_{3P}}{F_{2P}}\cdot\sigma_{2P}\cdot\eta_{2P}\cdot\frac{(RR_{3P}\cdot\tau_p{}^{3P})^2}{RR_{2P}\cdot\tau_p{}^{2P}}\cdot\frac{(\lambda_{exc}{}^{3P})^3}{\pi\cdot NA^2\cdot\lambda_{exc}{}^{2P}}\cdot 2.398\cdot\frac{\langle P^{2P}\rangle^2}{\langle P^{3P}\rangle^3} \quad (23).$$

For Eq. (22) and (23) we assumed that the concentration within each measured cell during the consecutive two- and three-photon excitation measurements at 1100 nm, 80 MHz and 1650 nm, 3 MHz remains the same.

1.4. Dependence of fluorescence signal and higher harmonics generation signals on the laser power.

The Eq. (20) for the fluorescence signal upon non-resonant two-photon excitation in logarithmic representation is given by:

$$\lg(F_{2P}) = \lg\left(\frac{1}{2}\bar{\varphi}\cdot\sigma_{2P}\cdot\eta_{2P}\cdot C\cdot\frac{g_P{}^{2P}}{RR_{2P}\cdot\tau_p{}^{2P}}\frac{\pi\cdot n}{\lambda_{exc}}\right) + 2\lg(\langle P^{2P}\rangle) \quad (24).$$

In analogy, the second harmonics generation (SHG) signal in logarithmic representation is given by Eq. (25). The difference between Eq. (24) and (25) is due to the fact that the SHG signal depends on the real part of the complex second order susceptibility χ_2, whereas the fluorescence signal upon a two-photon excitation depends on its imaginary part.

$$\lg(SHG) = \lg\left(\frac{1}{2}RE(\chi_2)\cdot\frac{g_P{}^{2P}}{RR_{2P}\cdot\tau_p{}^{2P}}\frac{\pi\cdot n}{\lambda_{exc}}\right) + 2\lg(\langle P^{2P}\rangle) \quad (25)$$

$RE(\chi_2)$ is the real part of the second order susceptibility.

In the same line, the fluorescence signal upon non-resonant three-photon excitation given by Eq. (21) in logarithmic representation turns into:

$$\lg\left(F_{3P}\right) = \lg\left(\frac{1}{3}\bar{\varphi}\cdot\sigma_{3P}\cdot\eta_{3P}\cdot C\cdot\frac{g_P{}^{3P}}{RR_{3P}{}^2\cdot\left(\tau_p{}^{3P}\right)^2}\cdot 0.544\cdot\frac{\pi^2\cdot n\cdot NA^2}{\lambda_{exc}{}^3}\right) + 3\lg\left(\langle P\rangle\right) \quad (26).$$

The third harmonics generation (THG) signal in logarithmic representation is given by:

$$\lg\left(THG\right) = \lg\left(\frac{1}{3}RE(\chi_3)\cdot\frac{g_P{}^{3P}}{RR_{3P}{}^2\cdot\left(\tau_p{}^{3P}\right)^2}\cdot 0.544\cdot\frac{\pi^2\cdot n\cdot NA^2}{\lambda_{exc}{}^3}\right) + 3\lg\left(\langle P\rangle\right) \quad (27)$$

with $RE(\chi_3)$ the real part of the third-order susceptibility.

For a resonance-enhanced three-photon excitation (RE3P), we expect the fluorescence signal to be a linear combination of a two-photon excitation process given by Eq(20) and a three-photon excitation process given by Eq(21), with the pre-factors a_{2P} and a_{3P}, respectively, the two pre-factors summing up to unity. In logarithmic representation, the fluorescence signal upon resonance-enhanced three-photon excitation is:

$$\lg\left(F_{RE3P}\right) = \lg\left[a_{2P}\cdot\left(\frac{1}{2}\bar{\varphi}\cdot\sigma_{2P}\cdot\eta_{2P}\cdot C\cdot\frac{g_P{}^{2P}}{RR_{2P}\cdot\tau_p{}^{2P}}\frac{\pi\cdot n}{\lambda_{exc}}\cdot\langle P\rangle^2\right) + a_{3P}\cdot\left(\frac{1}{3}\bar{\varphi}\cdot\sigma_{3P}\cdot\eta_{3P}\cdot C\cdot\frac{g_P{}^{3P}}{RR_{3P}{}^2\cdot\left(\tau_p{}^{3P}\right)^2}\cdot 0.544\cdot\frac{\pi^2\cdot n\cdot NA^2}{\lambda_{exc}{}^3}\cdot\langle P\rangle^3\right)\right] \quad (28).$$

With increasing laser excitation power, a_{3P} increases, turning Eq. (28) into Eq. (26) for non-resonant three-photon excitation, dependent only on the cubic power of the average laser power.

1.2. Laser power attenuation with tissue imaging depth (z)

As previously described, we can assume that the average laser power in a multi-photon setup exponentially decreases with tissue imaging depth z, mainly due to absorption and scattering of radiation:

$$\langle P\rangle(z) = \langle P\rangle(z=0)\cdot e^{-z/l_e} \quad (29)$$

with $\langle P\rangle(z=0)$ the average laser power at the surface of the specimen and l_e the effective attenuation length.

When combining Eq. (20) and (29), the depth-dependent fluorescence signal upon two-photon excitation, in natural logarithmic representation, is given by:

$$\ln\left(F_{2P}(z)\right) = \ln\left(\frac{1}{2}\bar{\varphi}\cdot\sigma_{2P}\cdot\eta_{2P}\cdot C\cdot\frac{g_P{}^{2P}}{RR_{2P}\cdot\tau_p{}^{2P}}\frac{\pi\cdot n}{\lambda_{exc}}\right) + 2\ln\left(\langle P(z=0)\rangle\right) - \frac{2z}{l_e} \quad (30).$$

In analogy, the depth-dependent SHG signal is given by:

$$\ln(SHG) = \ln\left(\frac{1}{2}RE(\chi_2)\cdot\frac{g_P{}^{2P}}{RR_{2P}\cdot\tau_p{}^{2P}}\frac{\pi\cdot n}{\lambda_{exc}}\right) + 2\ln(\langle P(z=0)\rangle) - \frac{2z}{l_e} \qquad (31).$$

The depth-dependent fluorescence signal upon three-photon excitation, also in natural logarithmic representation, is obtained when replacing Eq. (29) in Eq(. 21):

$$\ln(F_{3P}) = \ln\left(\frac{1}{3}\bar{\varphi}\cdot\sigma_{3P}\cdot\eta_{3P}\cdot C\cdot\frac{g_P{}^{3P}}{RR_{3P}{}^2\cdot(\tau_p{}^{3P})^2}\cdot 0.544\cdot\frac{\pi^2\cdot n\cdot NA^2}{\lambda_{exc}{}^3}\right) + 3\ln(\langle P(z=0)\rangle) - \frac{3z}{l_e} \qquad (32).$$

For the THG signal, the equation reads:

$$\ln(THG) = \ln\left(\frac{1}{3}RE(\chi_3)\cdot\frac{g_P{}^{3P}}{RR_{3P}{}^2\cdot(\tau_p{}^{3P})^2}\cdot 0.544\cdot\frac{\pi^2\cdot n\cdot NA^2}{\lambda_{exc}{}^3}\right) + 3\ln(\langle P(z=0)\rangle) - \frac{3z}{l_e} \qquad (33).$$

As we used laser power adaptation with depth (z) in our measurements, the power at surface *P(z=0)* is also a function of z. Both fluorescence and higher-harmonics generation signals were normalized for the corresponding *P(z=0)* at the respective imaging depth.

2.3. Fluorophore saturation limits the maximum imaging depth in tissues for a given pulsed excitation source.

To determine the supported repetition rate for a non-resonant (multi-photon) excitation, in a certain imaging depth in tissue, for a given average laser power at the tissue surface (z = 0), we rely on the equation:

$$RR_{3P} = \frac{\langle P(z=0)\rangle}{E_{focus}}\cdot e^{-z/l_e} \qquad (34),$$

with E_{focus} the pulse energy at the focus.

If E_{focus} exceeds the pulse energy E_{sat}, which leads to the saturation of the fluorophore, the fluorescence signal will not further increase. Experimentally, we avoid reaching the saturation regime, as shown by the double-logarithmic representations in Suppl. Fig. 2. In this way, we avoid a disproportionate increase of highly non-linear photobleaching, without increase of fluorescence signal.

For a three-photon excitation process[9], the saturation energy is given by: $E_{sat} = \frac{h\cdot c\cdot\lambda_{exc}}{\pi\cdot NA^2}\cdot\sqrt[3]{\frac{(\tau_p{}^{3P})^2}{g_P{}^{3P}\cdot\sigma_{3P}\cdot\eta_{3P}}}$. Hence, the maximum repetition rate is given by:

$$RR_{3P} = \frac{\langle P(z=0) \rangle}{\frac{h \cdot c \cdot \lambda_{exc}}{\pi \cdot NA^2} \cdot \sqrt[3]{\frac{(\tau_P{}^{3P})^2}{g_P{}^{3P} \cdot \sigma_{3P} \cdot \eta_{3P}}}} \cdot e^{-z/l_e} \quad (35).$$

2.4. Signal-to-noise ratio

The signal-to-noise ratio (SNR) in all cases, i.e. fluorescence upon non-resonant two-, non-resonant three-photon, or resonance enhanced three-photon excitation, as well as second and third harmonics generation, is defined as:

$$SNR = \frac{detected\ gray\ value - \langle background \rangle}{\sigma(background)} \quad (36).$$

We thereby assume that the background (recorded in regions with no tissue structure) is Gaussian distributed, with $\langle background \rangle$ the mean background value and $\sigma(background)$ the width of the Gaussian function. $\sigma(background)$ represents the background noise.

References

1 Horton, N. G. & Xu, C. Dispersion compensation in three-photon fluorescence microscopy at 1,700 nm. *Biomed Opt Express* **6**, 1392-1397 (2015). https://doi.org:10.1364/BOE.6.001392
2 Richards, B. W., E. Electromagnetic diffraction in optical systems, II. Structure of the image field in an aplanatic system. *Proc. Royal Soc. A* **253** (1959). https://doi.org:https://doi.org/10.1098/rspa.1959.0200
3 Gu, M. *Advanced Optical Imaging Theory*. (Springer, 2000).
4 Hontani, Y., Xia, F. & Xu, C. Multicolor three-photon fluorescence imaging with single-wavelength excitation deep in mouse brain. *Sci Adv* **7** (2021). https://doi.org:10.1126/sciadv.abf3531
5 Loudon, R. *The quantum theory of light, 2nd edition* (Claredon, 1983).
6 Herz, J. *et al.* Expanding two-photon intravital microscopy to the infrared by means of optical parametric oscillator. *Biophys J* **98**, 715-723 (2010). https://doi.org:10.1016/j.bpj.2009.10.035
7 Drobizhev, M., Tillo, S., Makarov, N. S., Hughes, T. E. & Rebane, A. Absolute two-photon absorption spectra and two-photon brightness of orange and red fluorescent proteins. *J Phys Chem B* **113**, 855-859 (2009). https://doi.org:10.1021/jp8087379
8 Shim, J., Iwaya, C., Ambrose, C. G., Suzuki, A. & Iwata, J. Micro-computed tomography assessment of bone structure in aging mice. *Sci Rep* **12**, 8117 (2022). https://doi.org:10.1038/s41598-022-11965-4
9 Choe, K. *et al.* Intravital three-photon microscopy allows visualization over the entire depth of mouse lymph nodes. *Nat Immunol* (2022). https://doi.org:10.1038/s41590-021-01101-1
10 Charan, K., Li, B., Wang, M., Lin, C. P. & Xu, C. Fiber-based tunable repetition rate source for deep tissue two-photon fluorescence microscopy. *Biomed Opt Express* **9**, 2304-2311 (2018). https://doi.org:10.1364/BOE.9.002304
11 Hopt, A. & Neher, E. Highly nonlinear photodamage in two-photon fluorescence microscopy. *Biophys J* **80**, 2029-2036 (2001). https://doi.org:10.1016/S0006-3495(01)76173-5

12 Logan, M. *et al.* Expression of Cre Recombinase in the developing mouse limb bud driven by a Prxl enhancer. *Genesis* **33**, 77-80 (2002). https://doi.org:10.1002/gene.10092
13 Jones, R. B. & Walker, B. D. HIV-specific CD8(+) T cells and HIV eradication. *J Clin Invest* **126**, 455-463 (2016). https://doi.org:10.1172/JCI80566
